# Comprehensive Characterization of a Next-Generation Antiviral T-Cell Product and Feasibility for Application in Immunosuppressed Transplant Patients

**DOI:** 10.3389/fimmu.2019.01148

**Published:** 2019-05-28

**Authors:** Leila Amini, Tino Vollmer, Desiree J. Wendering, Anke Jurisch, Sybille Landwehr-Kenzel, Natalie Maureen Otto, Karsten Jürchott, Hans-Dieter Volk, Petra Reinke, Michael Schmueck-Henneresse

**Affiliations:** ^1^Institute for Medical Immunology, Charité University Medicine Berlin, Berlin, Germany; ^2^Renal and Transplant Research Unit, Department of Nephrology and Internal Intensive Care, Charité University Medicine Berlin, Berlin, Germany; ^3^Berlin Institute of Health Center for Regenerative Therapies (BCRT), Charité University Medicine Berlin, Berlin, Germany; ^4^Berlin-Brandenburg School for Regenerative Therapies, Charité University Medicine Berlin, Berlin, Germany; ^5^Berlin Center for Advanced Therapies, Charité University Medicine Berlin, Berlin, Germany; ^6^Department for Pediatric Pulmonology, Immunology and Intensive Care Medicine, Charité University Medicine Berlin, Berlin, Germany

**Keywords:** cytomegalovirus, adoptive T-cell therapy, solid organ transplantation, Rapamycin, mTOR, immune regeneration

## Abstract

Viral infections have a major impact on morbidity and mortality of immunosuppressed solid organ transplant (SOT) patients because of missing or failure of adequate pharmacologic antiviral treatment. Adoptive antiviral T-cell therapy (AVTT), regenerating disturbed endogenous T-cell immunity, emerged as an attractive alternative approach to combat severe viral complications in immunocompromised patients. AVTT is successful in patients after hematopoietic stem cell transplantation where T-cell products (TCPs) are manufactured from healthy donors. In contrast, in the SOT setting TCPs are derived from/applied back to immunosuppressed patients. We and others demonstrated feasibility of TCP generation from SOT patients and first clinical proof-of-concept trials revealing promising data. However, the initial efficacy is frequently lost long-term, because of limited survival of transferred short-lived T-cells indicating a need for next-generation TCPs. Our recent data suggest that Rapamycin treatment during TCP manufacture, conferring partial inhibition of mTOR, might improve its composition. The aim of this study was to confirm these promising observations in a setting closer to clinical challenges and to deeply characterize the next-generation TCPs. Using cytomegalovirus (CMV) as model, our next-generation Rapamycin-treated (Rapa-)TCP showed consistently increased proportions of CD4^+^ T-cells as well as CD4^+^ and CD8^+^ central-memory T-cells (T_CM_). In addition, Rapamycin sustained T-cell function despite withdrawal of Rapamycin, showed superior T-cell viability and resistance to apoptosis, stable metabolism upon activation, preferential expansion of T_CM_, partial conversion of other memory T-cell subsets to T_CM_ and increased clonal diversity. On transcriptome level, we observed a gene expression profile denoting long-lived early memory T-cells with potent effector functions. Furthermore, we successfully applied the novel protocol for the generation of Rapa-TCPs to 19/19 SOT patients in a comparative study, irrespective of their history of CMV reactivation. Moreover, comparison of paired TCPs generated before/after transplantation did not reveal inferiority of the latter despite exposition to maintenance immunosuppression *post*-SOT. Our data imply that the Rapa-TCPs, exhibiting longevity and sustained T-cell memory, are a reasonable treatment option for SOT patients. Based on our success to manufacture Rapa-TCPs from SOT patients under maintenance immunosuppression, now, we seek ultimate clinical proof of efficacy in a clinical study.

## Introduction

Severe viral infections have a major impact on the clinical course of immunocompromised patients. Despite availability of powerful antiviral medication, cytomegalovirus (CMV) still accounts for significant morbidity and mortality in solid organ transplant (SOT) recipients (1). CMV can trigger direct and indirect morbidities such as chronic allograft rejection or in the case of kidney transplantation (KTx) chronic nephropathy (2, 3). Therapeutic control of CMV may be hampered by the development of anti-viral drug resistance (4). Moreover, after discontinuation of anti-viral prophylaxis, late-onset CMV disease frequently occurs and overall mortality is significantly higher in CMV-infected compared to uninfected KTx patients (1). Of note, T-cell-mediated anti-CMV immunity was reported to be predictive for the development of late-onset disease (5) and anti-CMV_IE−1_-specific CD8^+^ T-cell responses stratify risk of CMV disease in heart and lung transplant as well as KTx patients (6, 7). In addition, the magnitude of the CMV_pp65_- and CMV_IE−1_-specific T-cell responses turned out to be protective against complications with CMV in hematopoietic stem cell transplantation (HSCT) (8, 9). Consequently, regeneration of the endogenous T-cell response against these antigens, as aspired by AVTT, may prevent and reduce virus-associated morbidities/mortality in the SOT setting. Other viruses with impact on SOT outcomes are Epstein-Barr-virus and BK-virus, for which less efficient or no antiviral drugs are available. T-cells play a key role in protection from severe viral infections (7, 10, 11). Thus, adoptive T-cell therapy (AVTT) is a potent novel treatment strategy to tackle fatal viral complications in immunosuppressed transplant patients. Mechanisms of success or failure of new AVTT approaches need to be thoroughly understood and specific characteristics of patient cohorts have to be considered for successful translation of AVTT.

For clinical application of AVTT, *ex vivo* enrichment and expansion of virus-specific T-cells under GMP conditions are crucial and thus various protocols have been developed for CMV-specific AVTT after HSCT (12–17). However, the success of these approaches is limited in SOT patients due to the T-cell products (TCPs) being derived from patients instead of healthy HSCT donors, the lack of lymphodepletive preconditioning and the need for concomitant immunosuppression. Nevertheless, we and other groups demonstrated not only safety of AVTT, but also significant reduction of viral load and control of clinical symptoms of CMV disease in SOT recipients under maintenance immunosuppression in proof-of-concept studies (18–21). These observations are in line with positive results of AVTT for treatment of patients with EBV-related *post*-transplant lymphoproliferative disease (22–24). Yet, long-term efficacy failed in some patients, who experienced recurrence of CMV or EBV load and symptoms (18–25). To adapt AVTT to combat these clinical challenges, it is crucial to consider the respective patient cohort and the TCPs' characteristics. Specifically, our aim was to increase longevity of transferred T-cells to improve sustainability of clinical efficacy of AVTT in SOT patients. Failure of long-term control of CMV/EBV infections may be due to limited persistence of adoptively transferred T-cells *in vivo*, which might occur due to the late differentiation state implying limited longevity of infused T-cells. Therefore, advancing the quality of adoptively transferred TCPs with defined compositions by the enrichment for distinct T-cell memory subsets may improve therapeutic outcome. In particular, central-memory T-cells (T_CM_; CCR7^+^ CD62L^+^ CD45RO^+^ CD45RA^−^) and memory-stem T-cells (T_SCM_; CCR7^+^ CD62L^+^ CD45RO^−^ CD45RA^+^ CD95^+^) have high proliferative potential, self-renewal capacity and are reported to show superior engraftment, persistence, and survival compared to more differentiated memory T-cells (26–33). Conversely, late-differentiated short-lived effector-memory T-cells (T_EM_; CCR7^−^ CD62L^−^ CD45RA^−^CD45RO^+^) and terminally-differentiated effector T-cells (T_EMRA_; CCR7^−^ CD62L^−^ CD45RA^+^CD45RO^−^) exert immediate effector function, but fail to establish long-lasting protective memory, because of poor proliferative potential and limited survival following antigenic rechallenge (26, 27, 34). Remarkably, these observations match clinical data demonstrating T-cell reconstitution after HSCT and prevention of CMV disease related to T_CM_ proportions in peripheral blood (35). Direct sorting strategies to isolate only CMV-specific long-lived T-cells are barely feasible under GMP conditions and would yield very small cell numbers likely not sufficient for successful AVTT in immunosuppressed SOT recipients. To ensure applicability in a clinical setting, we recently optimized our GMP-conform manufacturing process for autologous virus-specific TCPs and succeeded in attenuating T-cell differentiation by treatment with low doses of Rapamycin (inhibits the *mechanistic-target-of-rapamycin-complex-1*: mTOR-C1, favorable results with 20 nM) during expansion cultures (18, 36, 37). This next-generation antiviral TCP comprises enriched proportions of early-differentiated T_CM_ being superior for AVTT (37–39). Furthermore, next-generation Rapamycin-treated (Rapa-)TCPs contain higher proportions of CD4^+^ T-cells (37) reported to improve clinical efficacy (29, 40, 41).

Detailed knowledge regarding the characteristics of Rapa-TCPs is a prerequisite for realization of clinical translation. Thus, we closely investigated the molecular properties of this Rapa-TCP regarding dependence on cytokine supplementation regiments during *in vitro* expansion, long-term stability, survival/sensitivity to apoptosis, metabolism, transcriptome, clonal composition, the role of the different memory T-cell subsets and applicability to SOT patient samples. Our data reveal a beneficial early differentiated phenotype, profound function, elevated clonal diversity, and superior survival of Rapa-TCPs compared to first-generation TCPs, which is further underlined and confirmed by a distinct gene expression signature revealed by mRNA sequencing.

We used *in vitro* models to mimic the situation of TCPs once injected into a patient coping with CMV disease, *i.e*., massive antigen exposure and withdrawal of Rapamycin. Here, we observed a preserved capacity for CMV-specific production of effector cytokines. Moreover, we tested manufacture of CMV-specific TCPs from material of patients with chronic end-stage renal disease (ESRD) before and after KTx to investigate the impact of chronic immunosuppression, showing no benefits of TCP generation before KTx. We further investigated the influence of CMV-reactivation history after KTx on the differentiation of virus-reactive memory T-cells and the resulting composition of untreated and Rapa-TCPs, implying feasibility of TCP generation from all groups investigated. This next-generation AVTT approach may also be applied to other viral specificities, such as EBV and BKV or even cancer-specific T-cells. Prospectively, implementation of next-generation AVTT may allow for reduction or complete ablation of toxic anti-viral medication and minimize the risk for virus-associated complications in the SOT setting.

## Methods

### Patients' and Healthy Donors' Blood Samples

Venous blood samples were collected from 19 healthy donors (HDs) (10 m/9 f; 25–81 years) and 19 KTx patients (11 m/8 f; 34–78 years; Table S2) of the Kidney Transplant Ambulance, Charité Virchow Klinikum, Berlin. We worked with buffy coats from 3 of the 19 different HDs to have sufficient cells for different cell sorting steps. Peripheral blood mononuclear cells (PBMC) were isolated by Biocoll Separating Solution density gradient centrifugation (Biochrom). The Charité Ethics Committee (IRB) approved the study protocol and all blood donors provided written informed consent. Detailed characteristics of each patient are presented in Table S2.

### Enrichment and Expansion of CMV-Specific T-Cells

CMV-specific TCPs were generated using a previously described technique (18, 36, 37, 42). Briefly, PBMCs were stimulated for 6 h with overlapping CMV_pp65/IE−1_ peptide pools (JPT Peptide Technologies; 0.5 μg/ml each). IFNγ-producing cells underwent positive selection using the IFNγ Secretion Assay—Cell Enrichment and Detection Kit according to the manufacturer's instructions (Miltenyi Biotec). Enriched IFNγ^+^ cells were cultured for 21 days in 96- or 24-well-plates with irradiated (30 Gy using a GSR D1 [Gamma-Service Medical GmbH]) autologous feeder cells (derived from 1/5 of the capture assays' negative fraction) added only at d0 in complete media (VLE RPMI 1640 supplemented with penicillin (100 IU/ml) and streptomycin [all from Biochrom] and 10% fetal calf serum [FCS, PAA]), supplemented with 10 ng/ml recombinant human (rh) IL-7 and rh IL-15 (CellGenix) or 50 U/ml IL-2 in humidified incubators at 37°C and 5% CO_2_. Cells were split 1:1 when 100% confluence was reached. For Rapa-TCPs, 20 nM of Rapamycin (Rapamune, Pfizer Pharma GmbH) were added every 2nd day or upon splitting starting from day 1 (37).

**Figure 1:**
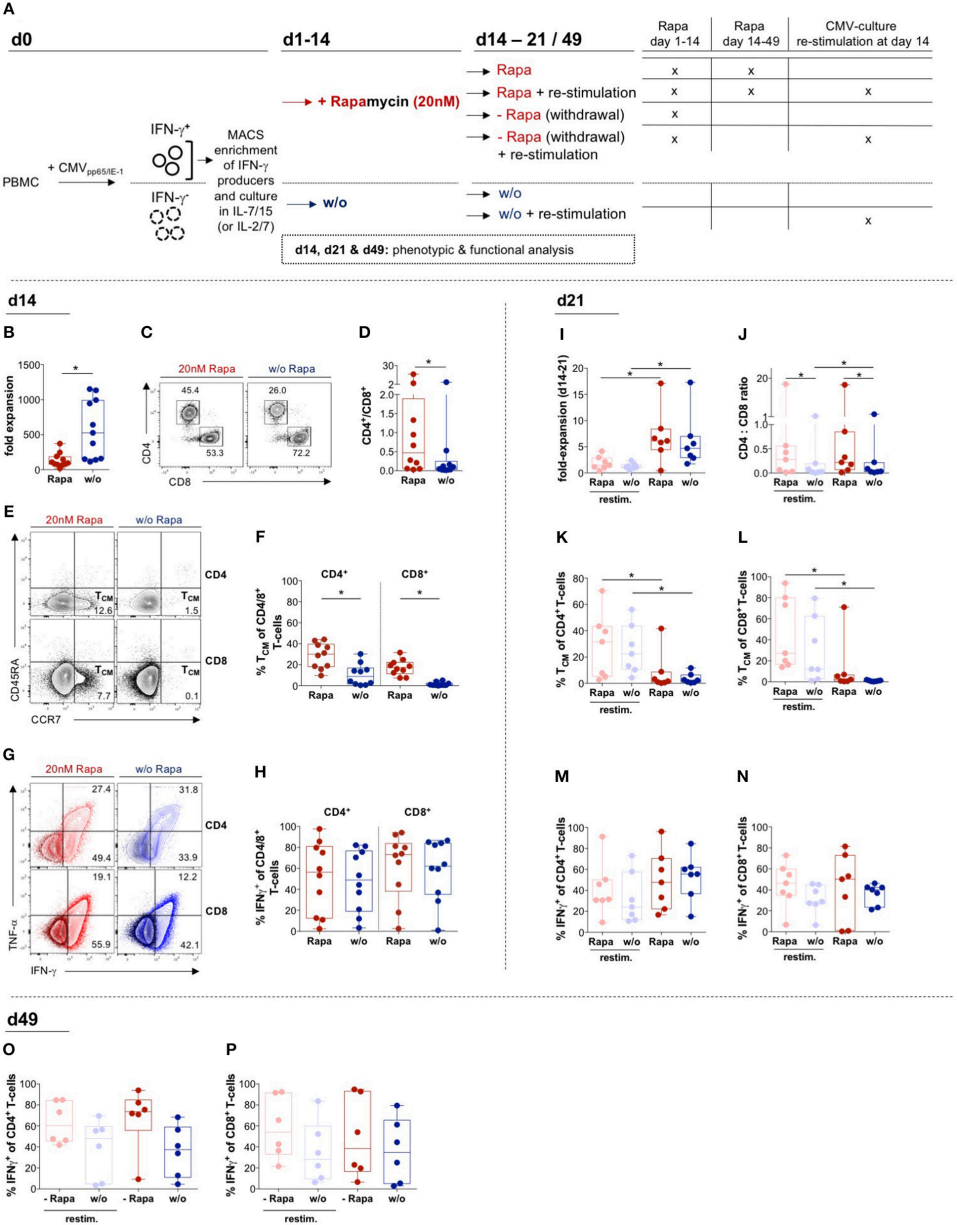
Effects of Rapamycin on T-cell products: Expansion, phenotype and function. **(A)** Schematic overview of experiments: T-cell products (TCPs) were generated from PBMCs isolated from venous blood of healthy donors (HDs) by magnetically activated cell sorting (MACS) of T-cells producing IFNγ in response to stimulation with CMV_IE−1/pp65_ peptide pools and expanded in the presence of either IL-2/IL-7 (Figure S1) or IL-7/IL-15 without (w/o; blue) or with addition of 20 nMof Rapamycin (Rapa; red) (**B–P**). Parts of the culture were re-stimulated using thawed CD3^−^ PBMCs loaded with CMV_IE−1/pp65_ peptide pools, deprived of Rapamycin or a combination of both on d14. **(B)** Expansion rates of IL-7/15-expanded Rapa-treated (Rapa-)TCPs (red) and untreated TCPs (blue) of *n* = 10 healthy donors (HDs) calculated from yield at d14 divided by the number of seeded cells at d0. We gated flow cytometric data on lymphocytes singlets living CD3^+^ T-cells. **(C)** Exemplary flow cytometry plots of CD4^+^ and CD8^+^ populations among living CD3^+^ T-cells in the Rapa-TCP (left plot) and untreated TCP (w/o, right plot) of one HD. **(D)** CD4/CD8 ratios in Rapa- (red) and untreated TCPs (blue) of *n* = 10 HDs calculated from flow cytometry data as presented in **(C)**. **(E)** Gating strategy for CD45RA^−^ CCR7^+^ central memory T-cells (T_CM_) among CD4^+^ (upper panel) and CD8^+^ (lower panel) in Rapa- (left panel) and untreated TCPs (right panel) of one exemplary HD. **(F)** Proportions of CD4^+^ and CD8^+^ T_CM_ among Rapa- (red) and untreated TCPs (blue) of *n* = 10 HDs determined from flow cytometric data as shown in **(E)** at d14. **(G,H)** To detect CMV-specific cytokine producers, TCPs were stimulated with CMV_IE−1/pp65_ peptide-loaded autologous lymphoblastic cell lines (LCLs) at a ratio of 1:10 for 6 h and Brefeldin A (BFA) was added after 1 h. **(G)** Representative flow cytometric plots of IFNγ- and TNFα-producers in Rapa- (left panel, red) and untreated TCPs (right panel, blue) of one HD. The dark population represents unstimulated and the light population illustrates CMV_IE−1/pp65_-stimulated CD4^+^ (upper panel) and CD8^+^ T-cells (lower panel). **(H)** Proportions of CMV-specific IFNγ-producers among CD4^+^ and CD8^+^ T-cells in Rapa- (red) and untreated TCPs (blue) of *n* = 10 HDs determined from flow cytometric data as shown in **(G)** at d14. **(I–N)**: For re-stimulation on d14 of culture, thawed CD3^−^ autologous PBMCs were loaded with CMV_IE−1/pp65_ peptide pools and added at 1:5 ratio to T-cells. **(I)** Expansion rates of IL-7/15-expanded re-stimulated (pastel colors) or non-re-stimulated (dark colors) Rapa- (red) and untreated TCPs (blue) of *n* = 7 HDs calculated from yield at d21 divided by the number of cells at d14. **(J)** CD4/CD8 ratios in Rapa- (red) and untreated TCPs (blue) of *n* = 7 HDs calculated from flow cytometric data as presented in **(C)** at d21. **(K**,**L)**: Proportions of CD4^+^ **(K)** and CD8^+^ T_CM_ **(L)** among Rapa- (red) and untreated TCPs (blue) of *n* = 7 HDs determined from flow cytometric data as shown in **(E)** at d21. **(M–P)** To detect CMV-specific cytokine producers, TCPs were stimulated with CMV_IE−1/pp65_ peptide-loaded autologous LCLs for 6 h and BFA was added after 1 h. **(M–N)** Proportions of CMV-specific IFNγ-producers among CD4^+^ **(M)** and CD8^+^ T-cells **(N)** in Rapa- (red) and untreated TCPs (blue) of *n* = 7 HDs determined from flow cytometric data as shown in **(G)** at d21. **(O,P)** To mimic the situation after infusion, Rapa was withdrawn and TCPs were cultivated long-term until d49. Proportions of CMV-specific IFNγ-producers among CD4^+^ **(O)** and CD8^+^ T-cells **(P)** in TCPs withdrawn from Rapa (red) and untreated TCPs (blue) of *n* = 6 HDs determined from flow cytometric data as shown in **(G)** at d49. For all graphs normal distribution of data points was tested with Kolmogorov-Smirnov test and paired *t*-test was used to determine significance in normally distributed samples or Wilcoxon's matched-pairs signed rank test in not normally distributed samples, respectively. *P*-values below 0.05 are indicated by * and defined to be significant.

For restimulation during culture (only where indicated, Figure 1, Figure S1), we depleted the donor's PBMCs of CD3^+^ cells using magnetically activated cell sorting (MACS) with anti-CD3 beads (Miltenyi Biotec) following the manufacturer's instructions. These cells were frozen in FCS with 10% cell culture grade dimethyl-sulfoxide (Sigma-Aldrich) until restimulation at d14. Thawed autologous CD3^−^ PBMCs were washed twice and pulsed with overlapping CMV_pp65/IE−1_ peptide pools at concentrations of 2 μg/ml each peptide for 2 h and added at a 1:5 ratio to the T-cells in culture.

### Functional Tests, Phenotyping, Flow Cytometry, and Sorting

Expanded T-cells were analyzed for effector functions by their ability to recognize antigen-loaded target cells, which consisted of autologous lymphoblastoid B-cell lines (LCLs), transformed with B95-8 EBV and by specific production of cytokines. LCLs were generated as described previously (43).

For CMV-specific stimulation of expanded TCPs for detecting intracellular effector cytokine production, CMV_pp65/IE−1_ peptide pool-loaded LCLs were added to cultured T-cells at a ratio of 1:10 and incubated for 6 h. Un-pulsed LCLs served as unstimulated control. For effector cytokine detection *ex vivo*, PBMCs were stimulated with 1 μg/ml overlapping CMV_pp65/IE1_ peptide pools *ex vivo* for 14 h. After 1 h, 2 μg/ml Brefeldin A (BFA, Sigma-Aldrich) were added to the stimulation to allow for intracellular capture of cytokines.

To induce apoptosis, 1 μg/ml of LEAF-purified Fas-activating antibody (EOS9.1; BioLegend) was added to cultures for 16 h. To determine survival, LIVE/DEAD® Fixable Blue Dead Cell Stain (Invitrogen) and Annexin V (BioLegend) were added.

For determination of killing capacity, autologous LCLs were pulsed with 2 μg/ml CMV_pp65/IE−1_ peptide pools, whereas unpulsed allogenic LCLs were used as non-target controls. Targets were labeled with 10 mM Carboxyfluorescein-diacetate-succinimidyl-ester (Sigma-Aldrich) and non-target controls with 5 mM CellTrace™ Far Red (Invitrogen). Cells were co-cultured at a T-cell/target-cell ratio of 10:1 for 14 h. Samples were analyzed using a LSR II Fortessa flow cytometer. Samples without T-cells, containing only LCLs, served as an internal control and reference for calculation of the killing capacity. For analysis, we gated on LIVE/DEAD® Fixable Blue Dead Cell Stain-negative cells and calculated ratios of target to non-target cells as described previously (44, 45).

To define memory subsets, T-cells were stained extracellularly for surface markers CCR7 (G043H7), CD45RA (HI100), CD45RO (UCHL1), CD62L (DREG-56; eBioscience), and CD95 (DX2). Subsequently, cells were permeabilized and fixed with Foxp3/Transcription Factor Staining Buffer Set (eBioscience) and stained intracellularly for CD3 (OKT3), CD4 (SK3), and CD8 (RPA-T8), IFNγ (4S.B3, eBioscience), TNFα (MAb11), and Granzyme B (GZB) (GB11, BD Pharmingen). Cells were analyzed on a LSR II Fortessa flow cytometer using FlowJo Version 10 software (Tree Star). Lymphocytes were gated based on the FSC *vs*. SSC profile and subsequently gated on FSC (height) *vs*. FSC to exclude doublets.

For evaluating of T-cell subsets on transcriptome level, T-cell subsets were sorted from PBMCs from *n* = 3 HDs' buffy coats (DRK) at d0 or derived TCPs at d18 based on the expression of CD3, CD45RA, and CCR7 by the Core Facility Flow Cytometry of the BCRT using a FACS Aria II Calliope (BD).

All antibodies were purchased from BioLegend, unless indicated otherwise.

### Metabolic Analysis

Extracellular acidification rate (ECAR) and oxygen consumption rate (OCR) were analyzed using a Seahorse-XFe96-Analyzer following the manufacturer's instructions for non-adherent cells including immobilization of cells with Cell-Tak (Corning). Assay medium consisted of Dulbecco's Modified Eagle's Medium D5030 (Sigma) supplemented with 3 g/l D-glucose (Roth) and 300 mg/ml L-glutamine (Gibco) and was sterile-filtered. For T-cell activation, 0.5 μg/ml of CMV _pp65/IE−1_ peptide pools were added to the microwells relying on reciprocal antigen-presentation of T-cells 0.5 h before the measurement.

### RNA Sequencing and Bioinformatics Analysis

RNA was isolated using an All-Prep DNA/RNA Kit (Qiagen) following the manufacturer's instructions. RNA samples were sent to the Deep Sequencing Core Facility in Göttingen, where samples were prepared using TrueSeq Kits (Illumina) and HiSeq_4000 performing 50 million reads/sample.

Fastq-files were quality checked with FastQC (Babraham Bioinformatics) and trimmed for residual adapter sequences. Reads were aligned to the GRCh38 human genome using TopHat^R^ (2.1.0–Johns Hopkins University, Center for Computational Biology) and Bowtie2 (46). Counts per gene were determined as sum of all reads mapped within a gene region. Principal component (PC) analysis was performed in R (47) using the 1,000 top-variable genes within the data set. Differentially expressed genes were identified using negative binomial distributions as implemented in the DESeq2 package (48) in R. False discovery rates (FDR) were calculated to adjust *p*-values for multiple testing and FDR-values below 0.05 were considered as significant. Expression data for differentially expressed genes were variance-stabilized transformed and scaled prior to visualization in heat maps. RNA sequencing data are available at the GEO platform with the accession number GSE129196.

### T-Cell Receptor Sequencing

For sequencing of T-cell receptors (TCRs) to determine the clonality of TCPs, DNA was isolated using an All-Prep DNA/RNA Kit (Qiagen) following the manufacturer's instructions. TCRβ sequencing was performed using a hsTCRb Kit (Adaptive Biotechnologies) following the manufacturer's instructions and analyzed with the corresponding ImmunoSEQ-Analyzer 3.0 software. Briefly, the most variable complementary-determining region 3 (CDR3), spanning the recombination site of V-D-J recombinations of TCR β-chains was sequenced. Productive rearrangements were regarded as unique in-frame nucleotide sequences without stop codon, leading to a functional TCR. Productive frequency means the individual frequency of a specific productive rearrangement (clone) among all productive rearrangements. Clonality was calculated based on productive entropy normalized to the total number of productive rearrangements. Sample overlap was investigated using the Morisita index considering unique clones, individual frequencies of clones and the probability of a common origin of two samples. TCR sequencing data is accessible at the ImmuneACCESS platform http://adaptivebiotech.com/pub/amini-2019-frontimmunol (Adaptive Biotechnologies).

### Statistical Analysis and Calculations

Graph Pad Prism version 7 was used for graph generation. To test for normal Gaussian distribution Kolmogorov-Smirnov test was performed. If data were normally distributed, Student's paired or unpaired *t*-test were employed for analysis. If data were not normally distributed, Wilcoxon's matched pairs test was applied to paired samples and Man-Whitney's test to unpaired samples. All tests were two-tailed. Probability (p) values of ≤0.05 were considered statistically significant and significance is denoted as follows: ^*^ = *p* < 0.05. Correlation analysis was assessed by Pearson's correlation coefficients for normally distributed data or non-parametric Spearman's rank correlation. Fold expansion expresses the manually counted cell count (Neubauer's counting chamber) excluding dead cells by Trypan blue staining (Sigma-Aldrich) at the day indicated divided by the initially seeded cell amount from the positive fraction of the IFNγ Secretion Cell Enrichment Assay.

All datasets are available upon reasonable request.

## Results

In order to prepare our approach for clinical translation, we deeply characterized functionality, stability and distinct molecular, metabolic and transcriptional properties of our next-generation Rapa-TCP, for which we applied mTOR inhibition by Rapamycin to enrich for CD4^+^ T-cells and CD4^+^/CD8^+^ T_CM_ (37). First, we addressed the question whether we can reproduce our findings and properties published for supplementation of a certain cytokine regiment, IL-2/IL-7, with a regiment commonly used for GMP applications by many groups, IL-7/IL-15, which was previously shown to support generation of T_CM_ (49).

### Supplementation of IL-7/IL-15 Does Not Alter Rapamycin-Mediated Effects in TCPs

To investigate potential differences in the effects of Rapamycin administration dependent on the cytokine regiment supplemented, we expanded CMV-specific T-cells in the presence of different cytokine combinations, namely IL-7/IL-15 (Figure 1A) and IL-2/IL-7 (37) (Figure S1A). The expansion rates of antigen-reactive T-cells were sufficient considering cell numbers used in a pilot study (18), although Rapamycin significantly reduced expansion in both IL-7/IL-15- (Figure 1B) and IL-2/IL-7-expanded TCPs (Figure S1B). Overall, different cytokines did not alter the beneficial effects of Rapamycin treatment (37): Rapamycin significantly increased CD4/CD8 ratio in both IL-7/IL-15- (Figures 1C,D) and IL-2/IL-7-expanded TCPs (Figure S1C) and significantly increased proportions of CD4^+^ and CD8^+^ T_CM_ in both IL-7/IL-15- (Figures 1E,F) and IL-2/IL-7-expanded TCPs (Figures S1D,E). Furthermore, Rapamycin increased proportions of Interferon-γ (IFNγ)-producing CD4^+^ and CD8^+^ T-cells upon exposure to CMV-specific peptides loaded onto autologous lymphoblastic cell lines (LCLs) in both IL-7/IL-15- (Figures 1G,H) and IL-2/IL-7-expanded TCPs (Figures S1F,G). These data confirm the robustness of beneficial effects of mTOR inhibition using Rapamycin for TCP composition in the case of supplementing commonly used IL-7/IL-15 for expansion of TCPs.

### Expansion Rates of Rapa-TCPs Recover Later During Culture

SOT patients often suffer from lymphopenia, which reduces the amount of PBMC, *i.e*., the starting material, for TCP generation and their medication can impact the functionality of T-cells (50). Thus, TCP manufacture from patient material may require longer *in vitro* expansion periods of up to 21 days to achieve sufficient cell numbers for successful AVTT. To assess the stability of TCPs after a longer period of expansion, we determined phenotype and functionality of TCPs after extended expansion on d21 in IL-7/IL-15- (Figures 1I,N) and IL-2/IL-7-expanded TCPs (Figures S1H,M). Interestingly, Rapa-TCPs recovered, yet even exceeded expansion of untreated TCPs in the 3rd week of expansion (d14–d21) (Figure 1I), which was significant in IL-2/IL-7-expanded TCPs (Figure S1H). CD4/CD8 ratios remained significantly higher in Rapa-TCPs at d21 (Figure 1J), but IL-2/IL-7-expanded TCPs showed significantly higher CD4/CD8 ratios than IL-7/IL-15-expanded TCPs at d21 (Figure 1J *vs*. Figure S1I). During expansion, T_CM_ differentiated and the enrichment of T_CM_ proportions upon Rapamycin-treatment lost significance in both IL-7/IL-15- (Figures 1K,L) and IL-2/IL-7-expanded TCPs (Figures S1J,K).

### Antigen Encounter Decreases Expansion Rates, but Promotes Less Differentiated Cells

We further mimicked the scenario happening once the TCPs are injected into a patient coping with CMV viremia in an *in vitro* model. Therefore, we modeled the situation of high antigen load by re-stimulation with CD3-depleted PBMCs pulsed with CMV-specific peptides: CMV-specific re-stimulation significantly reduced expansion rates in both IL-7/IL-15- (Figure 1I) and IL-2/IL-7-expanded TCPs (Figure S1H). Re-stimulation did not influence CD4/CD8 ratios in IL-7/IL-15- (Figure 1J) neither IL-2/IL-7-expanded Rapa-TCPs (Figure S1I), but significantly decreased CD4/CD8 ratios in IL-7/IL-15-expanded untreated TCPs (Figure 1J). Remarkably, re-stimulation significantly augmented the proportions of CD4^+^ and CD8^+^ T_CM_ in both IL-7/IL-15- (Figures 1K,L) and IL-2/IL-7-expanded TCPs (Figures S1J,K). However, re-stimulation decreased the proportion of CD4^+^ and CD8^+^ IFNγ-producers (Figures 1M,N), which was statistically significant in IL-2/IL-7-expanded Rapa-TCPs (Figures S1L–M).

### Rapamycin Preserves Superior Capacity for IFNγ Production

Importantly, IL-2/IL-7-expanded Rapa-TCPs showed significantly higher proportions of IFNγ-producers among CD4^+^ T-cells at d14 and d21 (Figures S1F,L) and CD8^+^ T-cells at d21 (Figure S1M) compared to untreated TCPs illustrating improved functionality. Because TCPs are deprived of Rapamycin and exposed to antigen once injected, we analyzed samples in which we withdrew Rapamycin and re-stimulated with CMV peptide-loaded CD3-depleted PBMCs on d14 (Figure 1A, Figure S1A). Interestingly, once treated with Rapamycin during the first 2 weeks of culture, both IL-7/IL-15- (Figures 1O,P) and IL-2/IL-7-expanded TCPs (Figures S1N,O) comprised more CD4^+^ and CD8^+^ IFNγ-producers continuously until d49 of culture.

### Rapamycin Enhances Survival of T-Cells

**Figure 2:**
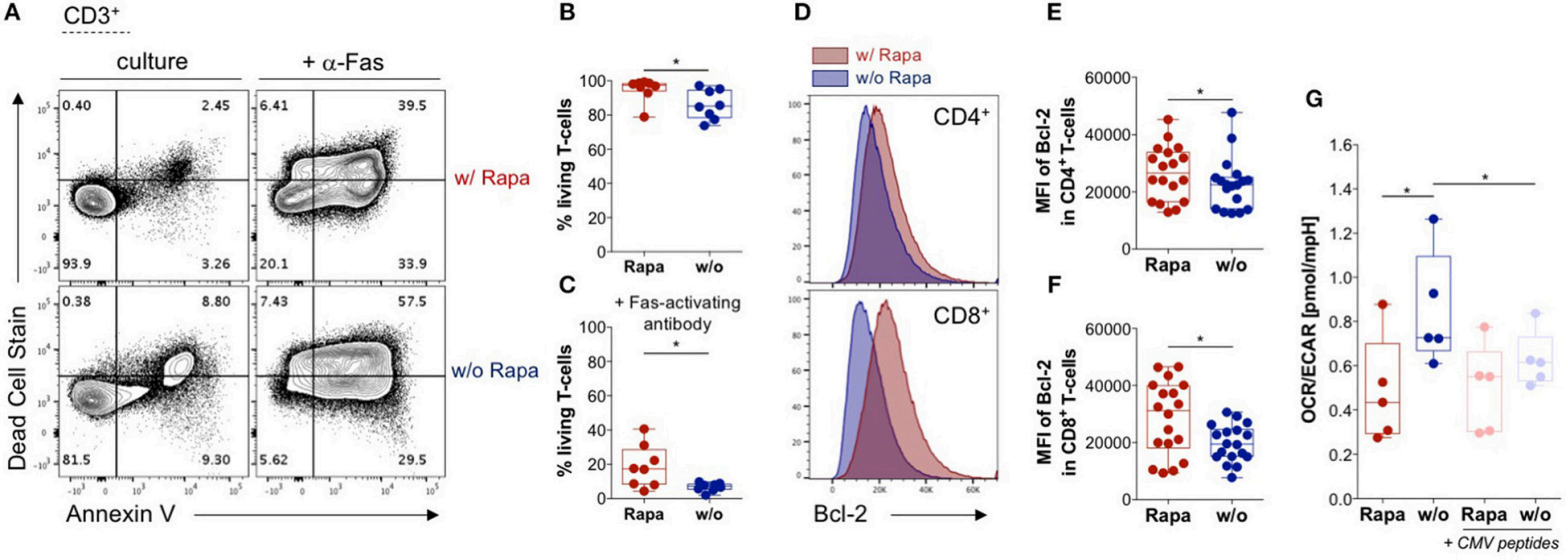
Rapamycin promotes survival of T-cells and stabilizes their metabolism. **(A)** Exemplary dot-plots of flow cytometry data regarding live/dead stain and Annexin V stain (apoptosis) gated on lymphocytes singlets CD3^+^ T-cells. Living T-cells are defined by double negative staining for Annexin V and live/dead stain in Rapa- (upper panel) and untreated TCPs (lower panel). Samples in the right panel were treated with 1 μg/ml activating antibody against Fas (CD95) to induce apoptosis. **(B)** Proportions of living T-cells in Rapa- (red) and untreated TCPs (blue) of *n* = 8 HDs identified as shown in **(A)** at d21. **(C)** Proportions of living T-cells in Rapa- (red) and untreated TCPs (blue) of n = 8 HDs incubated with Fas-activating antibody identified as shown in **(A)** at d21. **(D)** Exemplary histograms of fluorescence intensity of Bcl-2 in CD4^+^ (upper panel) and CD8^+^ T-cells (lower panel) of untreated (blue) and Rapa-TCPs (red) acquired by flow cytometry. **(E,F)** MFIs of Bcl-2 in CD4^+^ **(E)** and CD8^+^ T-cells **(F)** in untreated (blue) and Rapa-TCPs (red) of *n* = 18 HDs. **(G)** Oxygen consumption rare (OCR)/extracellular acidification rate (ECAR) ratio of Rapa- (red) and untreated TCPs (blue) of *n* = 5 HDs determined in a Seahorse assay. For stimulation (pastel colors) CMV_IE−1/pp65_ peptide pools were added to TCPs relying on mutual presentation of peptides by T-cells from the TCP. For all graphs normal distribution of data points was tested with Kolmogorov-Smirnov test and paired *t*-test was used to determine significance. *P*-values below 0.05 are indicated by * and defined to be significant.

Longevity is a crucial prerequisite for long-term efficacy of adoptively transferred TCPs in patients. Based on findings in B-cell lymphoma cell lines, we hypothesized that Rapamycin treatment may increase viability of T-cells (51). Thus, we analyzed overall survival of T-cells in TCPs. Strikingly, we found significantly higher proportions of living T-cells in TCPs treated with Rapamycin compared to untreated TCPs (Figures 2A,B).

Immunosuppressant regiments including Tacrolimus, which are commonly used in SOT, are reported to sensitize T-cells to programmed cell death (52). Hence, we investigated the TCPs' sensitivity to apoptosis employing induction of the death receptor pathway by agonistic Fas-specific antibody to identify differences between untreated and Rapa-TCPs. We recorded partial resistance to Fas-induced apoptosis in Rapa-TCPs, while untreated TCPs were more sensitive to Fas-induced apoptosis (Figures 2A,C). The anti-apoptotic effect of Rapamycin observed in B-cell lymphoma lines is reported to depend on upregulation of Bcl-2 on protein level (51). Hence, we assessed (Figure 2D) the mean fluorescence intensity (MFI) of Bcl-2 in TCPs and found significantly higher MFIs in CD4^+^ and CD8^+^ T-cells of Rapa-TCPs compared to untreated TCPs (Figures 2E,F). The findings regarding viability and resistance to apoptosis suggest an increased fitness of T-cells in Rapa-TCPs implying improved long-term survival *in vivo*.

### Rapamycin Stabilizes T-Cell Metabolism Upon Activation

Characteristically, memory and effector T-cells are distinguished by differences in metabolic activities (53). Thus, we investigated the ratio of fatty acid oxidation to glycolysis defined by the ratio of oxygen consumption to ECAR (OCR/ECAR). In fact, we detected significantly lower OCR/ECAR ratios in Rapa-TCPs compared to untreated TCPs (Figure 2G). Moreover, upon activation with CMV-specific peptides, the metabolism of Rapa-TCPs proofed to be more stable, whereas the OCR/ECAR ratio was significantly decreased in untreated TCPs (Figure 2G).

### Distinct Effects of Rapamycin on Isolated T-Cell Memory Subsets

**Figure 3:**
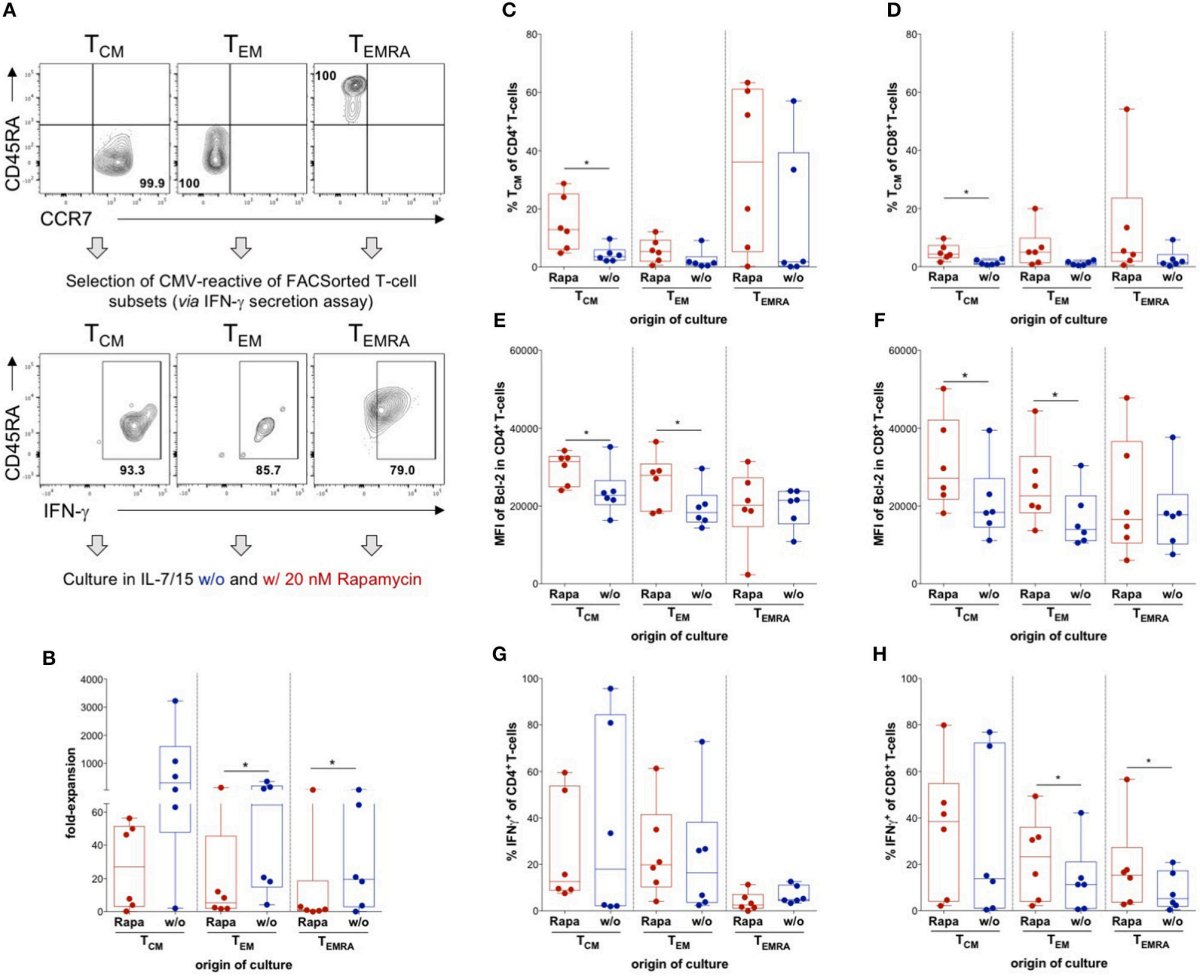
Influence of Rapamycin on different T-cell memory subsets. **(A)** Schematic experimental setup: T_CM_, T_EM_, and T_EMRA_ were sorted out of lymphocytes singlets CD3^+^ T-cells according to expression of CCR7 and CD45RA, CMV-reactive T-cells were isolated from each subset using an IFNγ secretion assay and CMV-reactive T-cells from each subset were cultured with (Rapa) and without Rapamycin (w/o). Exemplary dot plots of flow cytometry data of sorted subsets of one HD and respective positive fractions of the IFNγ secretion assay are shown. **(B)** Expansion rates of the indicated subsets in the presence of (red, Rapa) and absence of Rapamycin (blue, w/o) calculated from total cell numbers at d21 divided by the seeded cell number. **(C,D)** Proportions of CD4^+^ **(C)** and CD8^+^ CD45RA^−^ CCR7^+^ T_CM_-like cells **(D)** among Rapa-treated (red) and untreated (blue) cultures of indicated subsets determined from flow cytometric data at d21. **(E,F)** MFIs of Bcl-2 in CD4^+^ **(E)** and CD8^+^ T-cells **(F)** in untreated (blue) and Rapa-treated cultures (red) of isolated T-cell subsets determined in flow cytometry. **(G,H)** To detect CMV-specific cytokine production, cultures were stimulated with CMV_IE−1/pp65_ peptide-loaded autologous LCLs at a ratio of 1:10 for 6 h and BFA was added after 1 h. Proportions of CMV-specific IFNγ-producers among CD4^+^ **(G)** and CD8^+^ T-cells **(H)** in Rapa-treated (red) and untreated (blue) cultures of isolated T-cell subsets determined from flow cytometric data. All graphs contain data from *n* = 6 HDs, normal distribution of data points was tested with Kolmogorov-Smirnov test and paired *t*-test was used to determine significance in normally distributed samples or Wilcoxon's matched-pairs signed rank test in not normally distributed samples, respectively. *P*-values below 0.05 are indicated by * and defined to be significant.

Distinct memory T-cell subsets were reported to have defined properties and are not equally suited for long-term regeneration of T-cell immunity (26–33). To assess the effect of Rapamycin on distinct CMV-specific memory T-cell subsets, we performed fluorescently activated cell sorting (FACS) for CCR7^+^CD45RA^−^ T_CM_, CCR7^−^CD45RA^−^ T_EM_, and CCR7^−^CD45RA^+^ T_EMRA_ based on their differential expression of CD45RA and CCR7 *ex vivo* and subsequently performed IFNγ-secretion assays of sorted subsets to isolate CMV-specific T-cells of these particular subsets (Figure 3A). The CMV-reactive T-cells of each memory T-cell subset were expanded in the presence or absence of Rapamycin and analyzed after 3 weeks of culture. Interestingly, Rapamycin significantly reduced expansion in T_EM_- and T_EMRA_-derived cultures, which was not significant in T_CM_-derived cultures (Figure 3B), indicating preferential expansion of T_CM_ and implying reduced sensitivity to anti-proliferative effects of Rapamycin.

Moreover, Rapamycin prevented a significant proportion of CD4^+^ and CD8^+^ T_CM_ from differentiation into late-stage memory/effector T-cells compared to control cultures (Figures 3C,D). Remarkably, Rapamycin treatment even induced some cells with a T_CM_-like phenotype in cultures derived from T_EM_ and T_EMRA_ subsets suggesting some “rejuvenation” of late-stage memory cells (Figures 3C,D).

Notably, Rapamycin increased the MFI of Bcl-2 in CD4^+^ and CD8^+^ T-cells from T_CM_- and T_EM_-, but not T_EMRA_-derived cultures (Figures 3E,F). Interestingly, Rapamycin-treated T_EM_- and T_EMRA_-derived cultures contained significantly higher proportions of CD8^+^ IFNγ-producers than untreated cultures upon CMV-specific re-stimulation (Figure 3H), whereas there were no significant differences in CD4^+^ IFNγ-producers (Figure 3G). Overall, Rapamycin conferred distinct effects on different T-cell memory subsets, sustaining T_CM_ features and counteracting differentiation into late-stage memory/effector T-cells.

### Rapamycin-Treated T-Cell Products Have a Unique Transcriptome Resembling T_CM_

**Figure 4:**
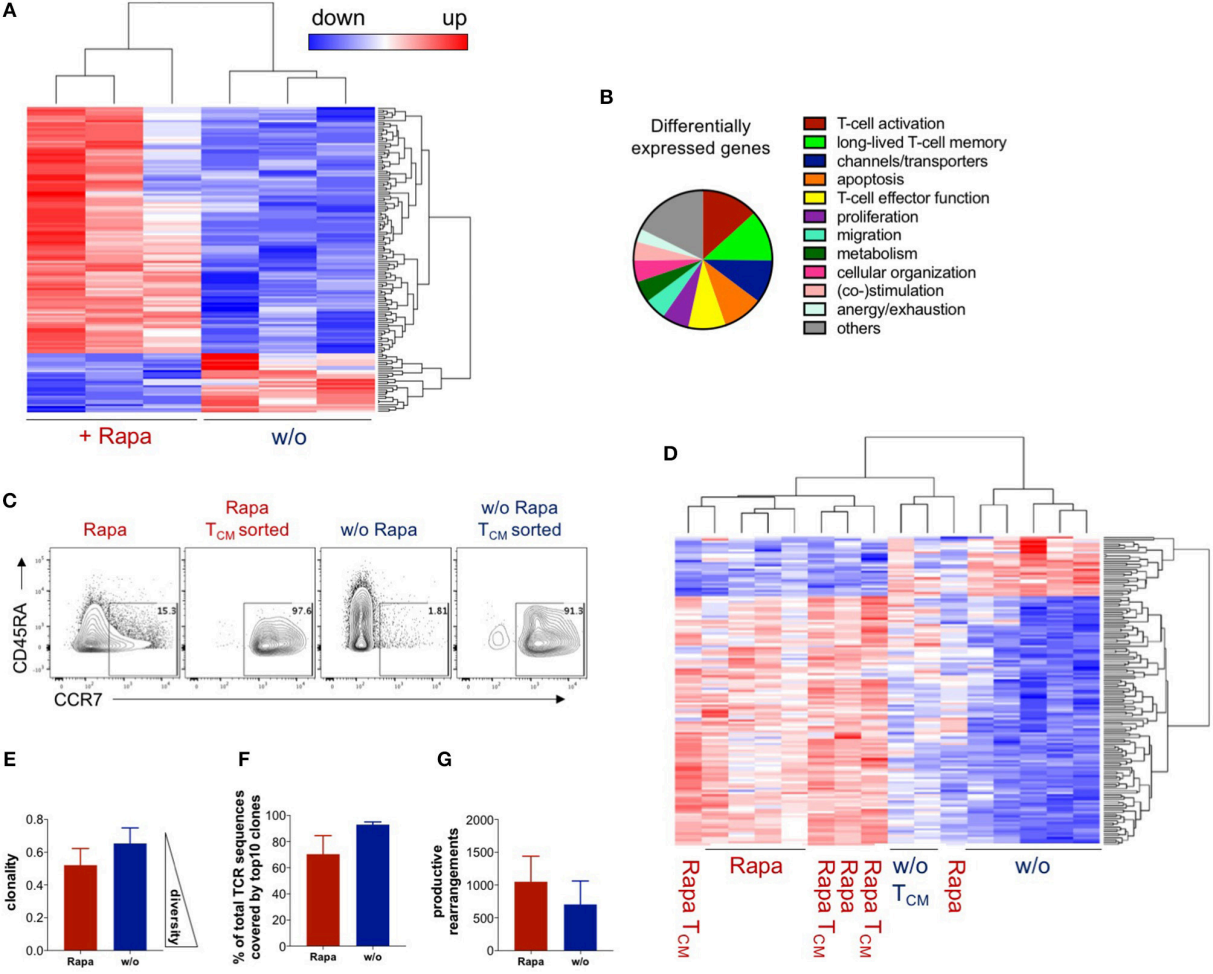
Rapamycin-treated TCPs have a unique transcriptome resembling T_CM_ and are clonally more diverse. RNA expression data were acquired by RNA sequencing and samples had to pass a quality control to be included in the analysis. **(A)** Expression heat map of differentially expressed genes between untreated and Rapa-TCPs generated from fresh blood of n = 3 HDs at d21. **(B)** Processes allocated to the differentially expressed genes based on the literature (see Table S1 for details). **(C)** Exemplary dot plots of CCR7/CD45RA expression of untreated and Rapa-TCPs and the respective T_CM_-like cells sorted on d18 of culture. **(D)** Expression of differentially expressed genes between Rapa- and untreated TCPs from fresh blood of *n* = 3 HDs at d21, buffy coats of *n* = 3 HDs at d18 and T_CM_ sorted from Rapa- and untreated TCPs generated from buffy coats of the same *n* = 3 HDs at d18 including clustering. Samples not included in the graph were discarded due to failure at the quality threshold. **(E)** Clonality of Rapa- (red) and untreated TCPs (blue) *n* = 3. **(F)** Proportions of represented sequences covered by the top 10 most represented clones in Rapa- (red) and untreated TCPs (blue), *n* = 3 HDs. **(G)** Numbers of productive rearrangements included in in Rapa- (red) and untreated TCPs (blue), *n* = 3 HDs. Data in **(E,F)** were calculated with ImmunoSEQ-Analyzer3.0 software based on TCRβ sequencing.

To confirm that expansion of antigen-reactive T-cells under Rapamycin treatment “freezes” an early memory T-cell stage, we tried to extend our analysis on transcriptome level by RNA-sequencing using next-generation sequencing (NGS) of untreated and Rapa-TCPs at d21 (Figure 4A). The RNA expression data revealed a total of 146 differentially expressed genes between Rapa-TCPs and untreated TCPs (Figure 4A). Many of these relate to TCP performance (Figure 4B, Table S1). We reviewed the literature and various databases to identify T-cell associated processes (Figure 4B) and to estimate the relevance of the differentially regulated genes (Table S1). With reference to previously published data, 84% of the genes identified as potentially relevant for TCP potency and longevity *in vivo* were regulated in a beneficial manner in Rapa-TCPs. Among these differentially expressed genes, we identified increased expression of T_CM_ markers such as *CCR7* and *PIM2* (54), increased expression of *TERT*, which induces self-renewal capacity and increases the proliferative potential of human T-cells (55), and *IL7R*, which is reported to be a marker for persisting and protective CD8^+^ memory T-cells (56) in Rapa-TCPs (Table S1). Furthermore, our observation of sustained IFNγ production in Rapa-TCPs is in line with the findings of increased expression of *IL-13* regulating IFNγ synthesis (57), *DRD2* inducing IFNγ production (58) and *TNFRSF11A*, which increases IFNγ secretion upon binding its ligand (59). In addition, these data are underlined by increased expression of activation enhancing genes, including *e.g., KLF7* (60)*, RGMB* (61), and *TNFRSF19* (62) in Rapa-TCPs. Moreover, the fact that anti-apoptotic Bcl-2 is upregulated on protein level may be supported by increased expression of *MYB*, which exerts its anti-apoptotic activity *via* Bcl-2 (63). However, also many other genes inhibiting apoptosis, such as *e.g., BEX2* (64) and *SIX1* (65), show significantly higher expression in Rapa-TCPs compared to untreated TCPs. The metabolic data of increased glycolysis in Rapa-TCPs are in line with increased expression of *EPAS1* (66), however, also *CHDH*, a gene involved in fatty acid oxidation, (67) is higher expressed in Rapa-TCPs than in untreated TCPs. See Table S1 for a complete view and annotation of the genes differentially expressed in untreated and Rapa-TCPs and their functions potentially relevant for TCP efficacy *in vivo*. Of note, the T_CM_-like cells sorted from untreated TCPs on day 18 of culture (Figure 4C) clustered with Rapa-TCPs (Figure 4D) regarding the differentially expressed genes identified in Figure 4A. Interestingly, principle component (PC) analysis revealed some components of the transcriptome of Rapa-TCPs to be more similar to *ex vivo*-sorted T_CM_ than T_EM_ (dimension PC2; Figure S2).

### Rapamycin-Treated T-Cell Products Show Less Clonal and More Diverse TCR Repertoires

To estimate the TCR repertoire of our TCPs, we performed TCRβ NGS. Notably, TCRβ sequencing showed a more diverse clonal composition of Rapa-TCPs compared to untreated TCPs (Figure 4E). Venn diagrams of the total numbers of clones and overlap between Rapa- and untreated TCPs are shown in Figure S3A and the distribution of the top 100 clones is shown in Figure S3B. Correspondingly, the top 10 clones covered around 70 and 90% of the whole TCRβ repertoire of Rapa-TCPs and untreated TCPs, respectively (Figure 4F) and Rapa-TCPs contained more different clones than untreated TCPs (Figure 4G). The top 10 shared clones and their respective frequencies in Rapa- and untreated TCPs are shown in Figure S3C. Comparison of unique nucleotide sequences revealed a high clonal overlap between the distinct Rapa- and untreated TCPs generated from the same donor (Figure S3D). In contrast, comparison of clonal repertoires between different individuals showed no overlap, confirming the specificity of the findings (Figure S3D).

### Onset of Immunosuppression in Patients Does Not Influence the Starting Material for TCPs Regarding T-Cell Differentiation and CMV-Specificity

As a prerequisite for clinical translation, we aimed at confirming feasibility of Rapa-TCP generation from patient blood and therefore collected samples from end stage kidney disease (ESRD) patients before and after kidney transplantation (KTx). To investigate the influence of immunosuppression on the starting material for TCPs, 7 paired samples from ESRD patients before/after KTx were analyzed *ex vivo*. All KTx recipients received standard immunosuppression (characteristics in Table S2, *pre/post-*Tx paired samples highlighted in gray). T-cells were divided into five differentiation subsets: CCR7^+^CD45RA^+^CD95^−^ T_N_ (naïve T-cells), CCR7^+^CD45RA^+^CD62L^+^CD45RO^−^CD95^+^ T_SCM_, CCR7^+^CD45RA^−^ T_CM_, CCR7^−^CD45RA^−^ T_EM_, and CCR7^−^CD45RA^+^ T_EMRA_ (Figures S4A,B) revealing no substantial differences between CD4^+^ and CD8^+^ memory T-cell subset distributions of paired patient samples before/after KTx (Figures S4C,D). To assess the phenotypic and functional characteristics of CMV-specific T-cells, PBMCs were stimulated with CMV_pp65/IE1_ peptides showing markedly higher frequencies of CMV-responsive T-cells among CD8^+^ compared to CD4^+^ T-cells (Figures S4E,F). However, frequencies of CMV-responsive T-cells were similar before and after KTx (Figures S4G,H) and T-cell memory subsets were comparable among CMV-responsive T-cells before and after KTx (Figures S4I,J).

### Manufacturing Rapa-TCPs Is Feasible Before and After Transplantation

**Figure 5:**
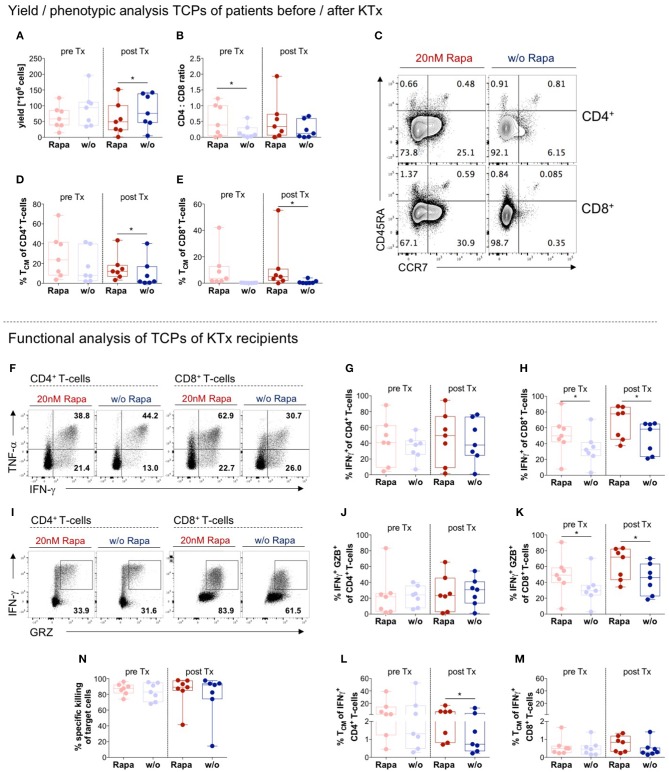
Manufacture of Rapa-TCPs is feasible before/after transplantation. *N* = 7 paired samples of untreated (w/o, blue) and Rapa-TCPs (red) from the same patients before (pre; pastel colors) and a few weeks after KTx (*post*). **(A)** Yield of TCPs = total cell number derived from 20 ml of patient blood on d21. **(B)** CD4/CD8 ratio of TCPs determined by multicolor flow cytometry on d14. **(C)** Exemplary dot plots of one patient's untreated (right) and Rapa-TCPs (left) comparing subset distributions of CD3^+^CD4^+^ (upper panel) and CD3^+^CD8^+^ (lower panel) T-cells according to CCR7 and CD45RA expression on d14. **(D,E)** Proportions of CD45RA^−^ CCR7 ^+^ T_CM_ among CD4^+^ **(D)** and CD8^+^ T-cells **(E)** in TCPs on d14 as determined per gating strategy shown in **(C)**. **(F)** Exemplary dot plots of one patient comparing CD3^+^CD4^+^ (left panel) and CD3^+^CD8^+^(right panel) IFNγ- and TNFα-producers in Rapa- (red) and untreated TCPs (blue) detected by intracellular staining in multicolor flow cytometry after 6 h stimulation with autologous LCLs loaded with CMV_IE−1/pp65_ peptide pools (gray) or incubation with unloaded autologous LCLs as control (black) and addition of BFA after 1 h on d21. **(G,H)** Summary of background subtracted proportions of CD4^+^ **(G)** and CD8^+^ **(H)** CMV-reactive IFNγ-producing T-cells in Rapa- (red) and untreated TCPs (blue) gated as illustrated in **(F)**. **(I)** Exemplary dot plots of one donor comparing CD4^+^ (left panel) and CD8^+^ (right panel) CMV-reactive IFNγ- and GZB-producers in Rapa- (red) and untreated TCPs (blue) detected by intracellular staining in multicolor flow cytometry after 6 h stimulation with autologous LCLs loaded CMV_IE−1_ and CMV_pp65_ peptide pools (gray), incubation with unloaded autologous LCLs as control (black) and addition of BFA after 1 h on d21. **(J,K)** Summary of background subtracted proportions of CD4^+^ **(J)** and CD8^+^ **(K)** CMV-reactive IFNγ/GZB-double-producers in Rapa- (red) and untreated TCPs (blue) gated as illustrated in **(I)**. **(L,M)** Proportions of CD45RA^−^ CCR7 ^+^ T_CM_ among CMV-reactive IFNγ-producing CD4^+^ **(L)** and CD8^+^ T-cells **(M)**. Gates were applied from gates set for global T-cell subset distribution (see Figure S4). **(N)** Specific killing of CMV_IE−1/pp65_ peptide pool loaded autologous LCLs determined by ratio with unloaded allogenic LCLs at a 1:10 ratio with TCPs after incubation overnight. All data tested for normal distribution of data points with Kolmogorov-Smirnov test; significance determined with paired *t*-test if normally distributed or Wilcoxon's matched-pairs signed rank test for not normally distributed samples. *P*-values below 0.05 are indicated by * and defined to be significant.

We assessed feasibility of TCP generation from patient material collected before and after KTx. Manufacture of untreated and Rapa-TCPs was successful with respect to yield (Figure 5A), although untreated CMV-specific TCPs resulted in higher yields, which was statistically significant in TCPs generated after KTx (Figure 5A). Rapa-TCPs showed beneficial, higher CD4^+^ T-cell proportions compared to untreated TCPs, which was statistically significant in TCPs generated before KTx (Figure 5B). Compared to untreated TCPs, Rapa-TCPs showed higher proportions of CD4^+^ and CD8^+^ T_CM_ in KTx recipient-derived TCPs, which was significant in TCPs generated after KTx (Figures 5C–E). Upon re-stimulation with CMV-peptide-loaded autologous LCLs, we found enhanced IFNγ-producers (Figure 5F) and IFNγ/granzyme B (GZB)-double-producers (Figure 5I) among Rapamycin-treated CD8^+^ T-cells (Figures 5H,K), whereas their proportions among CD4^+^ T-cells remained stable (Figures 5G,J). We recorded comparable frequencies of IFNγ-producers and IFNγ/GZB-double-producers among CD4^+^ T-cells in TCPs generated before and after KTx (Figures 5G,J), whereas both increased among CD8^+^ T-cells in TCPs generated after KTx (Figures 5H,K). Remarkably, Rapamycin increased the frequency of T_CM_ among IFNγ-producers (Figures 5L,M), which was statistically significant among CD4^+^ IFNγ-producers in TCPs generated after KTx (Figure 5l). Regarding CMV-specific cytotoxic effects, Rapa-TCPs were as effective as untreated TCPs and TCPs generated *pre*- and *post*-KTx neither showed any differences (Figure 5N).

### CMV History Affects the Composition of Starting Material From *Post*-KTx Patients

To determine the influence of the CMV infection status on the T-cell subset composition and function of starting material for TCP generation, 19 CMV seropositive KTx patients (Table S2) and 13 CMV seropositive healthy donors (HDs) were analyzed in parallel. Patients were categorized according to their CMV reactivation status: No recorded (*n* = 9; 5 m/4f), history of (*n* = 6; 2 m/4f) or very recent CMV-DNAemia within 2 weeks before blood collection (*n* = 4; 4 m/0f) (Table S2). The CMV reactivation status had almost no effect on the global CD4^+^/CD8^+^ T-cell memory subset distribution (Figures S5A–C,E,F,H–J), except for an increase in proportions of CD8^+^ T_SCM_ and a decrease in CD4^+^ T_EM_ in the blood of patients with no record of CMV viremia compared to HDs (Figures S5D,G).

We found CMV-reactive T-cells in all KTx patients and HDs, with markedly higher frequencies among CD8^+^ vs. CD4^+^ T-cells (Figures S6A,F). We did neither observe major differences in the magnitude of the CMV-response between KTx patients and HDs nor between the different groups of KTx patients (Figures S6A,F). Of note, proportions of below 0.2% of CMV-responsive T-cells among CD8^+^ T-cells occurred in 38.5% of HDs and only 10.5% of patients without recorded CMV-DNAemia (Figure S6F).

The majority of KTx patients showed T_SCM_ frequencies < 10% among CMV-reactive CD4^+^ T-cells. However, patients with a CMV history or recent CMV-DNAemia presented with significantly elevated CMV-reactive CD4^+^ T_SCM_ compared to KTx patients with no recorded CMV-DNAemia and HDs (Figure S6B). T_CM_ proportions among CD4^+^ CMV-responsive T-cells showed high inter-individual differences among the patients and HDs (Figure S6C). T_EM_ proportions among CD4^+^ CMV-responsive T-cells were significantly lower in the cohort of KTx patients with a record of CMV-DNAemia compared to patients with no recorded CMV-DNAemia and HDs (Figure S6D). The proportions of T_EMRA_ among the CD4^+^ CMV-responsive T-cells were below 5%, except for three patients with recent or previous CMV-DNAemia, who all received virostatic medication (Figure S6E, Table S2). We found no significant differences in the memory subset distribution among CMV-reactive CD8^+^ T-cells between the different patient groups and HDs (Figures S6G–J). Notably, we could not detect CMV-responsive CD8^+^ T_CM_ in the majority of samples (Figure S6H).

### Impact of CMV Reactivation State on Manufacture of Untreated and Rapa-TCPs

**Figure 6:**
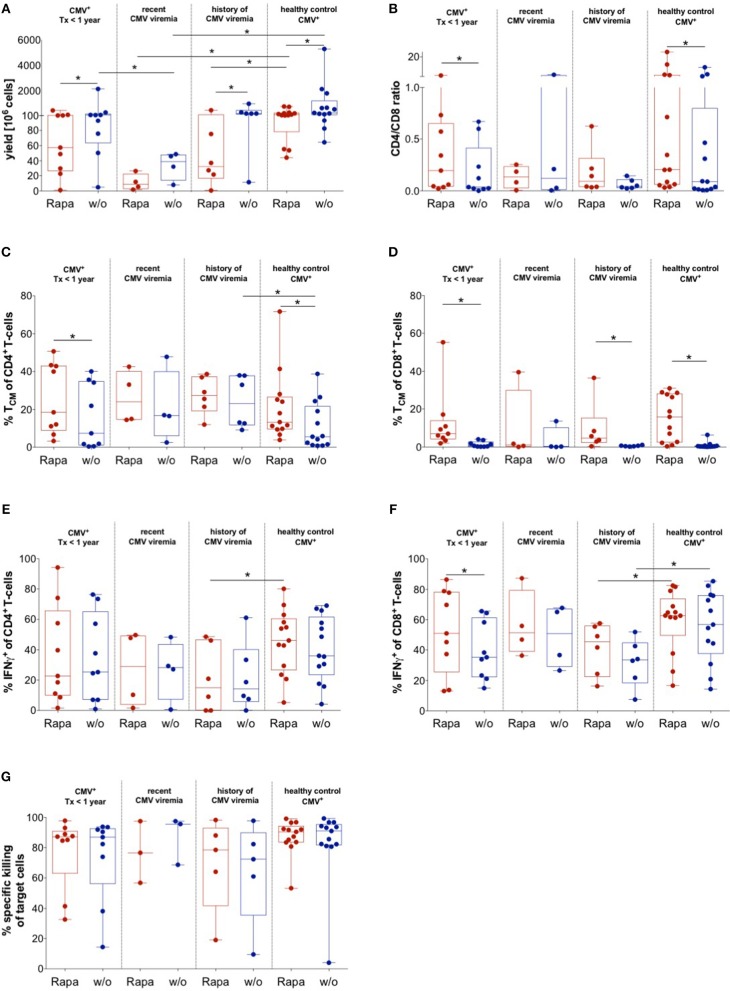
Impact of CMV history on manufacture of untreated and Rapa-TCPs. Untreated (w/o, blue) and Rapa-TCPs (red) of *n* = 19 patients (9 with so far no recorded CMV viremia; 4 with recent CMV viremia and 6 with a history of CMV viremia)/13 HDs. **(A)** Yield of TCPs = total cell number derived from 20 ml of patient blood on d21. **(B)** CD4/CD8 ratio of TCPs on d14. **(C,D)** Proportions of CD45RA^−^ CCR7 ^+^ T_CM_ among CD4^+^ **(C)** and CD8^+^ T-cells **(D)** in TCPs as determined per gating strategy shown in Figure 5C on d14. **(E,F)** Proportions of CMV-reactive CD4^+^ **(E)** and CD8^+^ **(F)** IFNγ-producers detected by intracellular staining in multicolor flow cytometry after 6 h stimulation with autologous LCLs loaded CMV_IE−1/pp65_ peptide pools at a ratio of 1:10 and addition of BFA after 1 h on d21. Gating strategy is shown in Figure 5F. **(G)** Specific killing of CMV_IE−1/pp65_ peptide pool loaded autologous LCLs determined by ratio with unloaded allogenic LCLs at 1:10 ratio with TCPs after incubation overnight. All data were tested for normality with Kolmogorov-Smirnov test; significant differences for paired samples determined with paired *t*-test if normally distributed or Wilcoxon's matched-pairs signed rank test and for unpaired samples with unpaired *t*-test if normally distributed or Man Whitney's test. *P*-values below 0.05 are indicated by * and defined to be significant.

To evaluate the quality of untreated and Rapa-TCPs generated from KTx patients under maintenance immunosuppression with distinct CMV reactivation states, CMV_pp65/IE1_-specific T-cells were expanded with or without Rapamycin (37). We successfully manufactured untreated and Rapa-TCPs from all KTx patients and HDs analyzed, although Rapamycin substantially reduced yields in TCPs from all patients and HDs (Figure 6A). Recent CMV reactivation further significantly reduced yields of untreated and Rapa-TCPs compared to HDs and history of CMV reactivation reduced yields of Rapa-TCPs compared to HDs (Figure 6A). Interestingly, there was an inverse correlation between expansion rate/yield and age in untreated and Rapa-TCPs (Figures S7A,B). Furthermore, the number of records with CMV-DNAemia correlated inversely with the yield of Rapa-TCPs (Figure S7C). We found that Rapamycin significantly increased the CD4^+^/CD8^+^ T-cell ratio in TCPs of KTx patients without recorded CMV-DNAemia and HDs, which was less pronounced in TCPs of KTx patients with a record of CMV-DNAemia (Figure 6B). Rapa-TCPs showed significantly higher proportions of CD8^+^ T_CM_ in all groups except the KTx patients with recent CMV DNAemia (*n* = 4) (Figure 6D), while CD4^+^ T_CM_ were only significantly enriched in TCPs of KTx patients with no recorded CMV-DNAemia and HDs, being less pronounced in TCPs from the other groups (Figure 6C).

Upon CMV-specific re-stimulation, we found increased frequencies of CD8^+^ IFNγ-producers in Rapa-TCPs, which was statistically significant in the group of KTx patients without record of CMV DNAemia (Figure 6F). However, Rapa-TCPs of KTx patients with a history of CMV DNAemia contained significantly lower frequencies of IFNγ-producers compared to Rapa-TCPs from HDs (Figures 6E,F). Remarkably, the frequency of IFNγ-producers among CD8^+^ T-cells, but not CD4^+^ T-cells (Figure S8A), inversely correlated with the time from the last CMV-DNAemia in untreated, but not Rapa-TCPs (Figure S8B). Notably, Rapa-TCPs included higher proportions of T_CM_-like among IFNγ-producing CD4^+^ and CD8^+^ T-cells compared to the corresponding untreated TCPs (Figures S8C,D). This was significant in IFNγ-producing CD4^+^ T-cells in TCPs of KTx patients without record of CMV DNAemia and IFNγ-producing CD8^+^ T-cells in TCPs of KTx patients without record and history of CMV DNAemia (Figures S8C,D). The T_CM_-like phenotype among IFNγ-producing CD4^+^ T-cells was significantly more frequent in Rapa-TCPs of KTx patients compared to HDs (Figure S8C). IFNγ-producing CD8^+^ T-cells were significantly more frequent in Rapa-TCPs of KTx patients with history of CMV viremia compared to HDs (Figure S8D).

In order to characterize functionality, we co-cultured TCPs with CMV-antigen-loaded LCLs for 14 h and killing was analyzed. Untreated and Rapa-TCPs achieved similar target cell lysis (Figure 6G). CMV-specific re-stimulation further characterized up to 65.6% of CD4^+^ T-cells to be cytotoxic as defined by GZB/IFNγ-double-production (Figure S8E). Interestingly, Rapa-TCPs of KTx patients with no record of CMV viremia contained significantly more GZB/IFNγ double producers among CD8^+^ T-cells than their untreated counterparts (Figure S8F). Rapa-TCPs of KTx recipients with history of CMV viremia contained significantly less GZB/IFNγ double producers among CD8^+^ T-cells than those of HDs (Figure S8F).

### Rapa-TCPs From Patients Exhibit Superior Viability After Thawing

**Figure 7:**
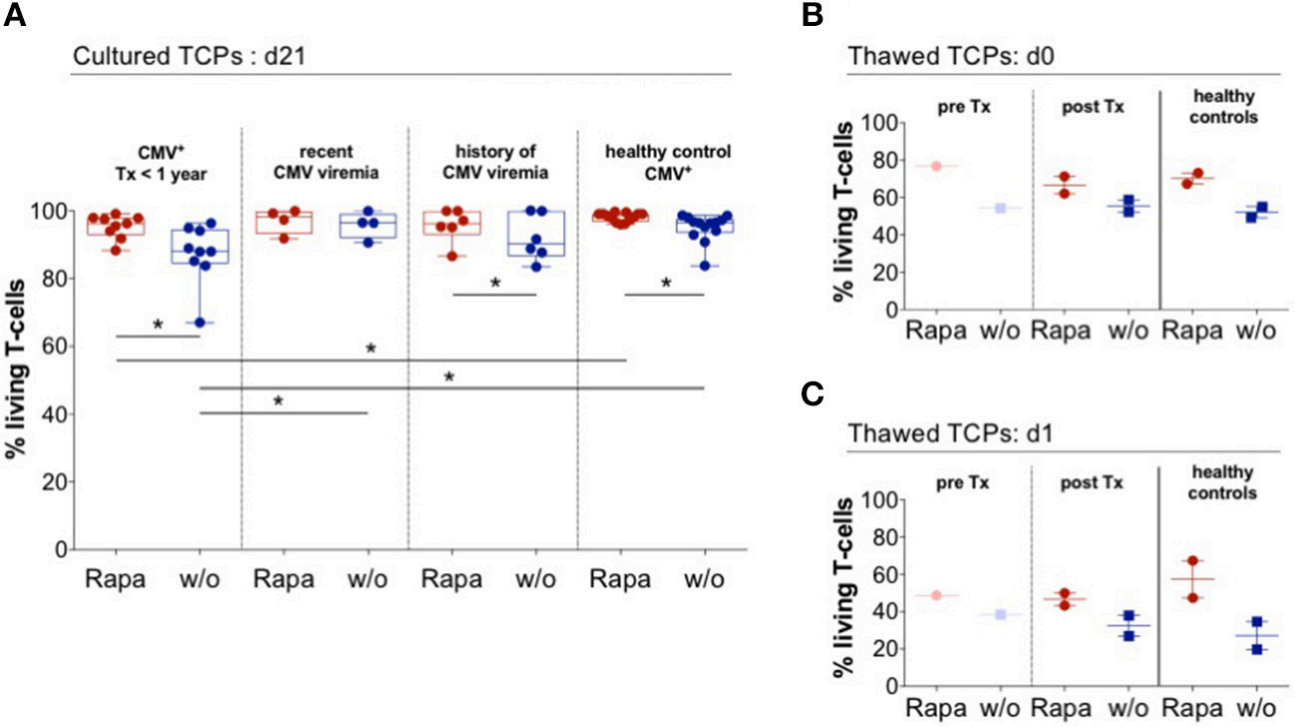
Rapa-TCPs from patients exhibit superior viability before and after thawing. Untreated (w/o, blue) and Rapa-TCPs (red) of *n* = 19 patients (9 with so far no recorded CMV viremia; 4 with recent CMV viremia and 6 with a history of CMV viremia)/13 HDs. **(A)** Proportions of living T-cells determined by positive staining for CD3 and negative staining for live/dead stain and Annexin V. **(B,C)** Untreated (w/o, blue) and Rapa-TCPs (red) from *n* = 4 (paired *pre*- and *post*-KTx samples of patient no. 11; *post*-KTx TCPs of patient no. 9 and two HDs). TCPs were frozen in fetal calf serum substituted with 10 % dimethylsulfoxide and stored in liquid nitrogen. After thawing and two washing steps, proportions of living T-cells defined by positive staining for CD3 and negative staining for live/dead stain and Annexin V were determined immediately (d0, **B**) and after 24 h rest in complete medium in a humidified incubator at 37°C and 5% CO_2_ (d1, **C**). All data were tested for normality with Kolmogorov-Smirnov test; significant differences for paired samples determined with paired *t* test if normally distributed or Wilcoxon's matched-pairs signed rank test and for unpaired samples with unpaired *t* test if normally distributed or Man Whitney's test. *P*-values below 0.05 are indicated by * and defined to be significant.

Strikingly, every single Rapa-TCP consistently comprised higher proportions of living T-cells compared to its untreated counterpart (Figure 7A). By convention, TCPs have to be frozen until GMP-compliant quality controls are accomplished and then are thawed directly before infusion into patients. This procedure is a major stress for the TCPs. We froze and thawed TCPs from ESRD/KTx patients and HDs and observed an increased frequency of living T-cells in the Rapa-TCPs, being detectable immediately and even 1 day after thawing and culture (Figures 7B,C). Consistent with the findings from HDs, also Rapa-TCPs of KTx patients showed elevated MFIs of Bcl-2 compared to the untreated TCPs (Figure S9).

In summary, we demonstrate that CMV-specific Rapa-TCPs can be generated irrespective of the employed cytokine regiment, show better viability even after thawing, a stable metabolism, beneficial gene expression and increased clonal diversity. We likewise demonstrate the possibility to generate TCPs from patients in ESRD and *post*-KTx despite maintenance immunosuppression containing similar attributes as from HDs. Further, we illustrate that functional CMV-specific T-cells, the prerequisite for manufacture of CMV-specific TCPs, could be identified in all KTx patients investigated and present successful manufacture of untreated and Rapa-TCPs irrespective of the viral replication history.

## Discussion

The aim of our study was to demonstrate the benefit of the Rapamycin treatment during manufacture of TCPs with improved properties. We demonstrate stability of Rapa-TCPs irrespective of cytokine combinations administered during expansion, sustained IFNγ production despite withdrawal of Rapamycin and re-stimulation with viral antigen. Furthermore, we found superior viability and partial resistance to death receptor-induced apoptosis, stable metabolism upon activation, favorable gene expression pattern, and enhanced clonal diversity of Rapa- compared to untreated TCPs. Moreover, we show preferential expansion of T_CM_ in the presence of Rapamycin and partial conversion of other T-cell memory subsets to T_CM_-like cells. We demonstrate the feasibility of manufacturing autologous anti-CMV Rapa-TCPs from blood of ESRD patients and KTx recipients with distinct CMV reactivation history. We confirm increased CD4/CD8 ratios and T_CM_ proportions in Rapa- compared to untreated TCPs (37), which are associated with long-term clinical efficacy of adoptively transferred TCPs (29, 30, 40, 41). In addition, our results imply that generation of CMV-specific TCPs prior to transplantation is possible, however, not favorable. Moreover, Rapa-TCPs from patients contained more viable cells after freezing/thawing compared to untreated TCPs.

Although the cytokine combination IL-7/IL-15 was reported to preferentially promote T_CM_ (49), we did not reveal substantial differences compared to cultures expanded with IL-2/IL7, suggesting these combinations are equally suited for TCP expansion and do not alter cell intrinsic mTOR-dependent signaling programs. Antigenic re-challenge and concomitant elimination of antigen-loaded APCs decreased T-cell expansion and resulted in T-cell culture contraction. These cultures predominantly contained long-lived memory T-cells. In line with this, among CMV-specific CD4^+^ T-cells, we recorded significant decreases in T_EM_ and significant increases in T_SCM_ in KTx recipients with a record of CMV DNAemia.

Rapa-TCPs consistently contained more living T-cells than untreated TCPs, even after freezing/thawing. Hence, Rapa-TCPs may entail increased fitness following infusion, as TCPs are frozen until accomplishment of all quality controls in a clinical setting respecting GMP/GCP. As shown previously for B-cell lymphoma lines (51), Rapamycin increased Bcl-2 on protein level and moreover led to partial resistance to Fas-induced apoptosis. We did not identify altered gene expression of *BCL-2* on mRNA level, however, expression of *MYB*, exerting anti-apoptotic effects *via* Bcl-2 (63), was upregulated in Rapa-TCPs. Furthermore, the decreased sensitivity to apoptosis of Rapa-TCPs may also be conferred by additional proteins and pathways as we found many other anti-apoptotic genes, such as *BEX2* (64) and *SIX1* (65), to be overexpressed in Rapa-TCPs compared to untreated TCPs.

We recorded higher OCR/ECAR ratios in untreated TCPs, although inhibition of glycolysis is associated with a long-lived memory phenotype (68) and memory T-cells are reported to preferentially perform fatty acid oxidation (53). Increased glycolysis in Rapa-TCPs was supported by RNA sequencing data showing increased expression of *EPAS1* (66), however, also *CHDH*, a gene involved in fatty acid oxidation (67), showed increased expression in Rapa-TCPs. In fact, glycolysis is reported to allow immediate effector function (69), which is in line with the Rapa-TCPs' enhanced capacity for IFNγ production and increased expression of *MAP3K21* allowing a rapid switch from a rested to an activated state (70). Furthermore, the metabolism of Rapa-TCPs remained more stable upon CMV-specific activation. Memory cell self-renewal might occur at a comparable number to that of effector T-cell generation in Rapa-TCPs, whereas in untreated TCPs, the balance might be extremely skewed toward effector T-cells upon activation, leading to this significant decrease in OCR/ECAR ratio and lack of long-lived memory T-cells.

Intriguingly, 84% of genes differentially expressed between untreated and Rapa-TCPs, whose function we could allocate to impact TCPs, were regulated toward promotion of an effective and long-lived product. Our data imply a T_CM_-like transcriptome of long-lived poly-functional memory T-cells for Rapa-TCPs (54–56, 66, 71–79). The fact that Rapa-TCPs show increased clonal diversity may occur due to survival of low frequency clones. Especially, the preferential expansion of T_CM_ in Rapa-TCPs may contribute to increased clonal diversity, as this subset was shown to have a higher clonal diversity compared to further differentiated memory T-cells (80). In fact, this may also be the underlying mechanism, why we have more CD4^+^ T-cells in the Rapa-TCP, because, evident from our *ex vivo* data, proportions of CMV-specific T_CM_ are much higher among CD4^+^ compared to CD8^+^ T-cells.

When CMV-specific T-cells from different memory T-cell subsets were cultured individually, we revealed preferential expansion of T_CM_ with preserved T_CM_ phenotype, partially protected from differentiation in the presence of Rapamycin, but partially also conversion of T-cells from other memory T-cell subsets into T_CM_-like cells (81). Interestingly, patient 12 lacked CMV-specific early CD8^+^ memory T-cells and his CMV-specific T-cells consisted to 90.6% of T_EMRA_. However, the respective Rapa*-*TCP included a strikingly high proportion of CD8^+^ T_CM_ suggesting reprogramming of T_EM_/T_EMRA_ to T_CM_.

We did not record major differences in the characteristics of the TCPs irrespective of whether the TCPs were generated *post*-KTx or *pre*-KTx. Nonetheless, CD8^+^ T-cells comprised higher proportions of T_CM_ and cytokine producers in TCPs generated *pre*-KTx. Hence, there is no benefit to generate anti-CMV TCPs prior to KTx.

Investigating *ex vivo-*T-cell responses to CMV-specific stimuli, we found higher frequencies of CD8^+^ than of CD4^+^ CMV-specific T-cells as described previously (82). Frequencies of below 0.2% of CMV-reactive CD8^+^ T-cells were found in 2/19 patients compared to 6/13 HDs, which matches findings suggesting an increase of CD8^+^ CMV-reactive T-cells *post*-Tx (83). Interestingly, low frequencies of CMV-reactive CD8^+^ T-cells did not cause low TCP yields, underlining the feasibility of TCP generation. Intriguingly, a high proportion of CD8^+^ T_EMRA_ among CMV-responsive T-cells did neither cause low expansion/yield nor high CD4/CD8 ratio in the TCP, which stresses the applicability even to patients with a high degree of terminal T-cell differentiation.

The dosage for successful CMV-specific AVTT in the SOT setting is undetermined. Case reports suggest numbers between 30 and 245 million T-cells (19–21, 36). Importantly, our protocol for TCP generation achieves medians of IFNγ-producers of 20 and 50% in CD4^+^ and CD8^+^ T-cells, respectively, and median killing rates of 85%, while these frequencies are much lower in other published approaches reporting a maximum of 8% of CMV-reactive IFNγ-producers in the TCP and specific lysis of ≤50% at higher T-cell/target ratios (19, 20). Comparable values are only achieved in a recent study (21). Based on the superiority in function and phenotype of Rapa-TCPs, we assume that also smaller T-cell numbers would be efficient for long-term control of CMV in KTx patients. The patients in this study whose TCPs yielded < 5 million T-cells from 20 ml of peripheral blood, included the two patients with the lowest lymphocyte counts (Patients 6/10), suggesting an amendment of the amount of blood collected to the lymphocyte count. Patient 6, with no recorded CMV DNAemia, was diagnosed with acute rejection and treated with ATG before blood collection. ATG administration may be an indication to collect blood for TCP generation preventively, given the patients' risk of developing CMV disease (84, 85). Indeed, transient CMV DNAemia was recorded in this patient 54 days after blood collection for the study. Patient 10 had a record of recent CMV viremia, was seronegative *pre*-KTx, received a kidney from a seropositive donor and had extensive CMV-associated complications. Notably, he was the oldest patient included and correlation analysis revealed a negative correlation between age and yield of Rapa-TCPs. We also recorded a low yield of the Rapa-TCP of Patient 19, who had a history of CMV-DNAemia and was receiving Acyclovir-treatment at the time of blood collection, which was reported to diminish IFNγ-production in response to CMV_pp65_ peptides (50). In cases as described above, we suggest to first generate untreated TCPs for patients with acute CMV disease to diminish viral load (36) and then successively generate and infuse Rapa-TCPs for long-term control of the virus. The fact that we recorded significantly lower yields in patients with a recent CMV DNAemia compared to HDs also suggests to follow the proposed approach in these patients and motivates to investigate more than the four patients that we were able to recruit for this study. In fact, we also found a negative correlation of the number of records with viremia and the yield of Rapa-TCPs for the ten patients with detected CMV DNAemia. However, this analysis may be biased by a more thorough screening of problematic patients, as the tests for CMV DNAemia were not of equal frequency in all patients. Moreover, patient 12, for whom we could also only generate a Rapa-TCP with a yield of around 5 million cells, suffered from chronic hepatitis. These, of course limited data, indicate that problems may occur during manufacture in the case of different chronic infections. It has to be thoroughly overthought whether it is possible to begin with more blood as starting material in these patients with numerous reactivating infections or use first the conventional approach and then generate a Rapa-TCP for long-term protection.

Recently, Smith and colleagues published a study about the application of a comparable autologous CMV-specific TCP in SOT patients (21). However, their production process varies in many points, as they do not select for CMV-specific T-cells starting their culture with PBMCs, use G-Rex reactors instead of classical well-plates for expansion and have a different cytokine supplementation strategy using IL-21 and IL-2. They infuse multiple doses of TCPs at up to 6 different points in time. Of note, they demonstrate safety and clinical improvement in the majority of patients and could decrease or stop antiviral medication in many patients. Interestingly, compared to the time of infusion, they see an increase in viral load in 9 of 13 patients after infusion of TCPs (21). In 5 of the 9 monitored patients CD8^+^ CMV-specific T-cells were reduced by the end of monitoring (max. day 300) and in three of the cases this correlated with an increase in viral load. One of the patients died of CMV disease (21). These data demonstrate, that there is still room for optimization in the long-term outcome of SOT patients treated with autologous CMV-specific TCPs. Probably, Rapa-TCPs could improve long-term efficacy, however, the actual clinical performance of our TCP has to be demonstrated. Our preclinical data imply a long-lived TCP with beneficial properties maybe even allowing for a single infusion.

A variety of other putative strategies are reported to rejuvenate T-cells with beneficial characteristics for AVTT. These include among others interference with different signaling pathways (86–90), use of different cytokine supplementation strategies (91, 92), employment of certain microRNAs (93), modulation of metabolism (53, 68), inhibition of ion channels (94), and promotion of autophagy (95). However, most of these are far from being practicable under GMP conditions for contemporary application to a clinical setting. This is also the case for genetic manipulation of T-cells for optimization of AVTT. Proposed strategies for genetic engineering include induction of resistance to immunosuppressive medication (96, 97), introduction of suicide genes as safety switch (49, 98) and knock out of anti-inflammatory signaling components such as PD-1 and LAG-3 (99, 100). All these suggestions have to be adapted to realistic GMP-feasible conditions and then may be valuable upgrades for even more sophisticated AVTT approaches. In contrast to other approaches, whose translation is less progressed at the moment, our minimally manipulative next-generation anti-CMV AVTT may help many transplanted patients whose endogenous immune system is not capable of defying the virus. Furthermore, the beneficial properties of Rapa-TCPs may also be transferred to other approaches using antigen-specific T-cells, e.g., other viruses or cancer immunotherapy with known antigens (42).

In summary, our study revealed favorable phenotypic and functional properties of Rapa-TCPs as well as their applicability to a variety of ESRD/KTx patient samples. Ultimately, we seek for clinical confirmation of functionality and efficacy of Rapa*-*TCPs in a clinical proof-of-concept trial.

## Ethics Statement

The Charité Ethics Committee (IRB) approved the study protocol and all blood donors provided written informed consent.

## Author Contributions

LA, H-DV, PR, and MS-H conceptualized and designed the study. PR and NO provided patient samples and data. LA, TV, DW, and AJ performed experiments supervised by MS-H. LA acquired, analyzed, and interpreted data. LA and MS-H composed figures and manuscript. KJ performed bioinformatics analyses, created the respective graphs and made RNA seq data available at the GEO platform. TV, DW, SL-K, KJ, H-DV, and PR critically revised and all authors approved the final version of the manuscript.

### Conflict of Interest Statement

H-DV, PR, and MS-H own a patent on the manufacture of Rapa-TCPs. The remaining authors declare that the research was conducted in the absence of any commercial or financial relationships that could be construed as a potential conflict of interest.
