# Supplementary material for: Comprehensive Characterization of a Next-Generation Antiviral T-Cell Product and Feasibility for Application in Immunosuppressed Transplant Patients
Source: Front Immunol. 2019 May 28;10:1148. doi: 10.3389/fimmu.2019.01148 (PMC6546853; doi:10.3389/fimmu.2019.01148)
Supplement: Supplementary file 1 [file Presentation_1.pdf]

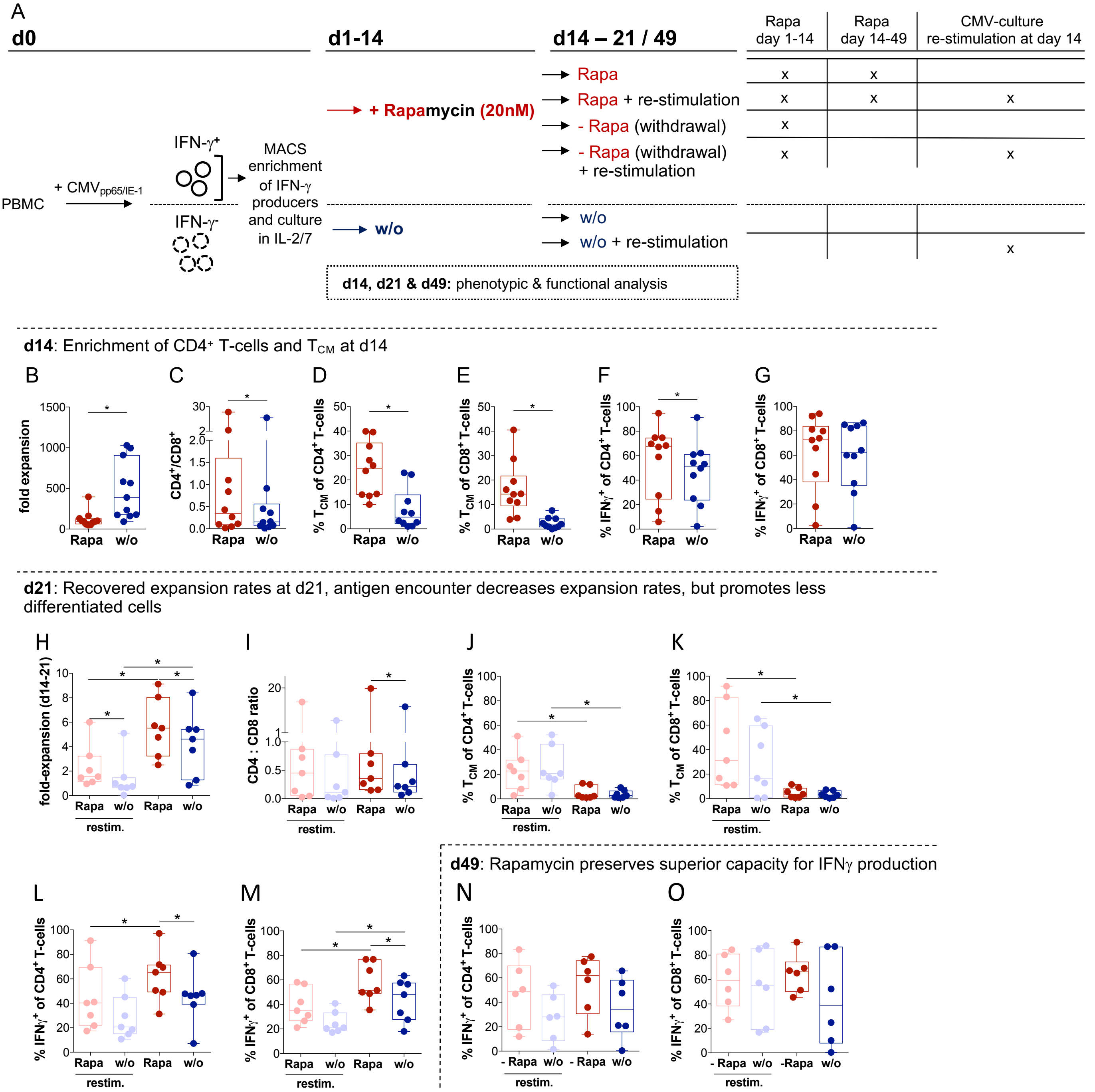

**Figure S1:** Effects of Rapamycin on IL-2/IL-7-expanded T-cell products: Expansion, phenotype and function

**A:** Schematic overview of experiments: T-cell products (TCPs) were generated from PBMCs isolated from venous blood of of healthy donors (HDs) by magnetically activated cell isolation sorting (MACS) of T-cells producing IFNγ in response to stimulation with $CMV_{IE-1/pp65}$ peptide pools and expanded in the presence of IL-2/IL-7 without (w/o; blue) or with addition of 20 nM of Rapamycin (Rapa; red). **B:** Expansion rates of IL-2/7-expanded Rapa-treated (Rapa-)TCPs (red) and untreated TCPs (blue) of n=10 healthy donors (HDs) calculated from yield at d14 divided by the number of seeded cells at d0. We gated flow cytometric data on lymphocytes → singlets → living $CD3^{+}$ T-cells. **C**: CD4/CD8 ratios in Rapa- (red) and untreated TCPs (blue) of n=10 HDs calculated from flow cytometry data as presented in Fig. 1C. **D-E**: Proportions of $CD4^{+}$ (**D**) and $CD8^{+}$ $T_{CM}$ (**E**) among Rapa- (red) and untreated TCPs (blue) of n=10 HDs determined from flow cytometric data as shown in Fig. 1E at d14. **F-G:** To detect CMV-specific cytokine producers, TCPs were stimulated with $CMV_{IE-1/pp65}$ peptide-loaded autologous LCLs at a ratio of 1:10 for 6 h and BFA was added after 1 h. Proportions of CMV-specific IFNγ-producers among $CD4^{+}$ (**F**) and $CD8^{+}$ T-cells (**G**) in Rapa- (red) and untreated TCPs (blue) of n=10 HDs determined from flow cytometric data as shown in Fig. 1G at d14. **H-O**: For restimulation on d14 of culture, thawed $CD3^{-}$ autologous PBMCs were loaded with $CMV_{IE-1/pp65}$ peptide pools and added at 1:5 ratio to T-cells. **H:** Expansion rates of IL-2/7-expanded restimulated (pastel colors) or non-restimulated (dark colors) Rapa- (red) and untreated TCPs (blue) of n=7 HDs calculated from yield at d21 divided by the number of cells at d14. **I**: CD4/CD8 ratios in Rapa- (red) and untreated TCPs (blue) of n=7 HDs calculated from flow cytometric data as presented in Fig. 1C at d21. **J-K**: Proportions of $CD4^{+}$ (**J**) and $CD8^{+}$ $T_{CM}$ (**K**) among Rapa- (red) and untreated TCPs (blue) of n = 7 HDs determined from flow cytometric data as shown in Fig. 1E at d21. **L-M**: To detect CMV-specific cytokine producers, TCPs were stimulated with $CMV_{IE-1/pp65}$ peptide-loaded autologous LCLs for 6 h and BFA was added after 1 h. Proportions of CMV-specific IFNγ-producers among $CD4^{+}$ (**L**) and $CD8^{+}$ T-cells (**M**) in Rapa- (red) and untreated TCPs (blue) of n=7 HDs determined from flow cytometric data as shown in Fig. 1G at d21. **N-O**: To mimic the situation after infusion, Rapa was withdrawn and TCPs were cultivated long-term until d49. Proportions of CMV-specific IFNγ-producers among $CD4^{+}$ (**N**) and $CD8^{+}$ T-cells (**O**) in TCPs withdrawn from Rapa (red) and untreated TCPs (blue) of n=6 HDs determined from flow cytometric data as shown in Fig. 1G at d49. For all graphs normal distribution of data points was tested with Kolmogorov-Smirnov test and paired t test was used to determine significance in normally distributed samples or Wilcoxon's matched-pairs signed rank test in not normally distributed samples, respectively. P-values below 0.05 are indicated by * and defined to be significant.

# **Table S1**: Differentially expressed genes.

Log2-fold describes log2-fold expression of the respective gene in untreated *vs*. Rapa-TCPs.

| | Ensemble ID | Gene | Function in T-cells | baseMean | log2Fold |
|---|---|---|---|---|---|
| **LONG-LIVED MEMORY** | | | | | |
| ***promoting*** | | | | | |
| | ENSG00000164530 | PI16 | unique subset of memory T helper $_{(H)}$ cells with hyperproliferative and proinflammatory properties (1) | 138.857891 | -4.52567 |
| | ENSG00000183813 | CCR4 | immature CD8 T-cell memory subset (2) | 3238.81399 | -3.97324 |
| | ENSG00000126353 | CCR7 | $T_{CM}$ marker (3) | 2184.22723 | -3.76273 |
| | ENSG00000056736 | IL17RB | highest expression in CD4$^+$ $T_{CM}$ expressing prostaglandin receptor D2 ($T_{H2}$) (4) | 3717.7612 | -3.196748 |
| | ENSG00000204252 | HLA-DOA | non-classical MHC class II (peptide loading), higher expression in CD8 CD28$^+$ T-cells from young than from old subjects (5) | 1385.33723 | -2.298921 |
| | ENSG00000073331 | ALPK1 | target of Tcf-1 (6) downstream of wnt (arresting memory CD8$^+$ T-cell differentiation [7]) | 404.02766 | -1.892146 |
| | ENSG00000135318 | NT5E | CD73 catalyzes the conversion of extracellular nucleotides to membrane-permeable nucleosides, CD73$^+$ mouse memory CD4$^+$ T-cells make IL-2, but less IFNg than CD73$^-$ (8) | 279.662059 | -2.320378 |
| | ENSG00000164362 | TERT | Telomerase reverse transcriptase, overexpression extends proliferative potential of human T-cells (9) | 27.7024907 | -3.430006 |
| | ENSG00000102096 | PIM2 | allows $T_{reg}$ expansion in Rapamycin (10), antiapoptotic/upregulated in $T_{CM}$ *vs.* $T_{EM}$ (11) | 7944.0909 | -1.735721 |
| | ENSG00000116016 | EPAS1 | accumulates upon early TCR signaling and hypoxia, accumulation increases glycolysis/GZB, expression diminished upon administration of IL-2, increased upon administration of IL-4, sustained effector function of CD8 CTLs (12) | 3957.09145 | -1.554453 |
| | ENSG00000168685 | IL7R | identifies the effector CD8 memory T-cell precursors that can persist and confer protective immunity (13) | 11182.7835 | -2.010491 |
| | ENSG00000132170 | PPARG | required for long-term persistence of CD8$^+$ T-cells in the skin, upregulated on $T_{RM}$ (14) | 391.15806 | -2.143364 |
| | ENSG00000072110 | ACTN1 | downregulated in effector/exhausted murine T-cells compared to memory T-cells (15) | 1155.07765 | -1.455754 |
| | ENSG00000143674 | MAP3K21 | bivalent methylation in CD8$^+$ $T_{CM}$ and $T_{EM}$ allows rapid switch from a resting to activated state, transcription increased upon activation (16) | 193.136063 | -1.700155 |
| | ENSG00000140443 | IGF1R | increased in autoimmunity, increased upon TCR stimulation, ligand binding enhances proliferation and survival of CD45RO$^+$ T-cells (17) | 797.424827 | -1.791784 |
| | ENSG00000115896 | PLCL1 | CpG methylation decreased in memory *vs.* naive CD4$^+$/upregulated upon activation with CD3/CD28 for 48 h (18), downregulated in SLE CD4$^+$ T-cells compared to controls (19) | 2484.05367 | -2.706713 |
| ***antagonizing*** | | | | | |
| | ENSG00000162490 | DRAXIN | antagonist of Wnt signaling (20) | 221.619933 | 1.841851 |
| | ENSG00000148053 | NTRK2 | downregulated on CD4$^+$ CD62L$^+$ T-cells compared to CD62L$^-$ (21) | 608.962631 | -2.751809 |
| | ENSG00000149295 | DRD2 | dopamin receptor, drives $T_{H1}$ differentiation *via* T-bet and IFNg production, decreases CD25 and CD69, advances T-cell differentiation, helps NFkB activation (22) | 30.4424689 | -3.125411 |
| **EFFECTOR FUNCTION** | | | | | |
| | ENSG00000173334 | TRIB1 | in transplant patients marker of ongoing immune activation and an antibody response (23), d0 higher expression in $T_{reg}$ than $T_{conv}$, but d7 of culture higher expression in $T_{conv}$ than $T_{reg}$ (24) | 550.825277 | -2.851495 |
| | ENSG00000137265 | IRF4 | induced by TCR signaling → upregulation of inhibitory receptor/impairs metabolism → T-cell exhaustion (25); induction dependent on the signal strength of TCR signaling → glycolysis/clonal expansion/maintenance of effector function (26) | 7024.41145 | -1.335874 |
| | ENSG00000189013 | KIR2DL4 | receptor for HLA-G, activation prevents cytolysis, HLA-G increases expression on CD4$^+$ T-cells (27), increased demethylation of promotor and expression in effector *vs.* memory T-cells (28) | 19.9418867 | 3.054789 |
| | ENSG00000124772 | CPNE5 | expression higher in effector than memory CD4$^+$ T-cells (29) | 60.6283057 | 1.900004 |
| | ENSG00000168229 | PTGDR | higher expression in $T_{EMRA}$ than other T-cells (30) | 371.569601 | 2.575663 |
| | ENSG00000165025 | SYK | switches TCR signaling from naive/memory to effector CD4$^+$ T-cells (31) | 131.519078 | 2.816866 |
| | ENSG00000170854 | RIOX2 | represses expression of IL-4 (32) | 1604.81806 | -1.44225 |
| | ENSG00000099985 | OSM | pro-inflammatory cytokine, attracts macrophages, induces antiviral response in epithelial cells (33; 34) | 1983.6564 | -1.986649 |
| | ENSG00000182568 | SATB1 | required for suppressive function of $T_{reg}$ and suppression of effector differentiation (35) | 11007.1072 | -1.273391 |
| ***cytotoxicity*** | | | | | |
| | ENSG00000108176 | DNAJC12 | chaperone, co-factor of Hsp70 (36), which induces cytotoxic CD4$^+$ T-cells (37) | 113.63151 | -3.063666 |
| | ENSG00000116016 | EPAS1 | accumulates upon early TCR signaling and hypoxia, accumulation increases glycolysis/GZB, expression diminished upon administration of IL-2, increased upon administration of IL-4, sustained effector function of CD8 CTLs (12) | 3957.09145 | -1.554453 |
| ***IFNg*** | | | | | |
| | ENSG00000169194 | IL13 | synergizes with IL-2 in regulating interferon-gamma synthesis (38) | 481.746448 | -2.918254 |
| | ENSG00000149295 | DRD2 | dopamin receptor, drives $T_{H1}$ differentiation *via* T-bet and IFNg production, decreases CD25 and CD69, advances T-cell differentiation, helps NFkB activation (22) | 30.4424689 | -3.125411 |
| | ENSG00000141655 | TNFRSF11A | RANK, induces T-cell survival upon binding of RANKL (39); RANKL increases IFNg secretion T-cells (40) | 1997.55921 | -2.813665 |
| **APOPTOSIS** | | | | | |
| ***anti-apoptotic*** | | | | | |
| | ENSG00000118513 | MYB | promotes memory T-cell development, antiapoptotic via Bcl-2 (41) | 1103.23321 | -3.78842 |
| | ENSG00000141655 | TNFRSF11A | RANK, induces T-cell survival upon binding of RANKL (39); RANKL increases IFNg secretion in effector and memory T-cells (40) | 1997.55921 | -2.813665 |
| | ENSG00000134278 | SPIRE1 | involved in DNA repair (42) | 295.111297 | -2.942319 |
| | ENSG00000134853 | PDGFRA | anti-apoptotic signaling via PI3K/migration (43) | 124.06083 | -4.193055 |
| | ENSG00000205755 | CRLF2 | thymic stromal lymphopoietin receptor, closely related to gamma chain, expression increased after TCR activation, signaling increases survival of T-cells and proliferation of activated CD4$^+$ T-cells (44) | 130.168039 | -1.904047 |
| | ENSG00000126778 | SIX1 | anti-apoptotic (45) | 95.8255104 | -3.407385 |
| | ENSG00000102096 | PIM2 | allows $T_{reg}$ expansion in Rapamycin (10), antiapoptotic/upregulated in $T_{CM}$ *vs.* $T_{EM}$ (11) | 7944.0909 | -1.735721 |
| | ENSG00000136026 | CKAP4 | p63, can suppress p73-dependent apoptosis (46) | 825.271346 | -2.023946 |
| | ENSG00000140443 | IGF1R | increased in autoimmunity, increased upon TCR stimulation, ligand binding enhances proliferation and survival of CD45RO$^+$ T-cells (17) | 797.424827 | -1.791784 |
| | ENSG00000180543 | TSPYL5 | decreases p53 levels (47) | 98.8277414 | -1.991591 |
| | ENSG00000133134 | BEX2 | protects against mitochondrial apoptosis, positively regulates Bcl-2/NFkB pathway and negatively regulates BAD, BAK1 and PUMA (48) | 199.28867 | -2.151393 |
| | ENSG00000011028 | MRC2 | Mannose receptor, extends survival of mouse thymocytes/maintains cell growth (49) | 268.370067 | -3.100144 |
| | ENSG00000171860 | C3AR1 | activation by C3a enhances naive CD4$^+$ T-cell activation, survival and proliferation *via* PI3K/Akt (50) | 200.019164 | 2.171075 |
| ***pro-apoptotic*** | | | | | |
| | ENSG00000101752 | MIB1 | E3 ubiquitin ligase, antagonizing anti-apoptotic effects of DAPK1 to promote TNF-induced apoptosis (51) | 7311.96339 | -1.07792 |
| | ENSG00000163568 | AIM2 | sensor of cytoplasmic DNA activating inflammasome and cells death (52) | 539.862663 | -1.605941 |
| **T-CELL ACTIVATION** | | | | | |
| | ENSG00000128322 | IGLL1 | downregulated within 48 - 96 h after T-cell activation (53) | 28.8060963 | -3.188403 |
| | ENSG00000114739 | ACVR2B | downregulated upon activation in naive T-cells (54) | 800.556947 | -1.03528 |
| | ENSG00000183395 | PMCH | expressed activation dependently in $T_{H2}$, but not $T_{H1}$ cells (55), upregulated in induced Treg (56) | 523.594842 | -1.970519 |
| ***enhancement*** | | | | | |
| | ENSG00000139946 | PELI2 | cooperates with Bcl-10 to activate NFkB in mice; can activate MAPK and AP-1/Elk-1 in humans (57) | 2995.12322 | -3.12598 |
| | ENSG00000134460 | IL2RA | CD25, sign for terminal differentiation in chronic viral response (58) | 6206.12175 | -2.47528 |
| | ENSG00000105246 | EBI3 | part of IL-27 and IL-35, expressed in CD4/CD8 T-cells upon activation with phytohemagglutinin and IL-27 (59) | -4.276035 | 7.89E-09 |
| | ENSG00000118263 | KLF7 | upregulated in activated CD8+ T-cells (60) | -2.276037 | 1.10E-08 |
| | ENSG00000147408 | CSGALNACT1 | associated with EAE (61) | -2.046745 | 8.22E-07 |
| | ENSG00000104427 | ZC2HC1A | variant increases susceptibility to MS (62) | -2.444141 | 9.39E-07 |
| | ENSG00000171860 | C3AR1 | activation by C3a enhances naive CD4$^+$ T-cell activation, survival and proliferation *via* PI3K/Akt (50) | 2.171075 | 5.64E-06 |
| | ENSG00000168811 | IL12A | required for development of EAE (63), upregulates IL12R → signaling induces T-bet (64) | -2.749976 | 1.79E-05 |
| | ENSG00000151748 | SAV1 | part of hippo signaling (induces a rather inflammatory T-cell phenotype [65]) | -1.437997 | 2.07E-05 |
| | ENSG00000214711 | CAPN14 | calpain, can generate constitutively active calcineurin (66) | -2.500567 | 3.49E-05 |
| | ENSG00000174136 | RGMB | ligand for PD-L2, not increased upon T-cell activation and absent in naive mice, sets down the threshold for activation and supports T-cell expansion (67) | -2.892547 | 0.000115 |
| | ENSG00000127863 | TNFRSF19 | increases proliferation and NFkB signaling (68) | -2.607645 | 0.000155 |
| | ENSG00000111252 | SH2B3 | LNK, involved in TCR signaling via PI3K/PLCg1/Ras (69) | 1.245567 | 0.000290 |
| ***negative regulation*** | | | | | |
| | ENSG00000188822 | CNR2 | cannabinoid receptor 2, inhibits inflammatory cytokines/proliferation of T-cells (70) | 264.665093 | 2.458243 |
| | ENSG00000133069 | TMCC2 | upregulated in the absence of Itk which is downstream of TCR signalling (71) | 139.965562 | -1.640783 |
| | ENSG00000154127 | UBASH3B | negatively regulates TCR signaling (72) | 880.828175 | 1.94863 |
| | ENSG00000107679 | PLEKHA1 | reduces activation of PI3K ⁄ Akt and STATs via PTPL1 (73) | 1089.23164 | 1.942193 |
| | ENSG00000071575 | TRIB2 | negative regulator of mitogen-activated protein kinases, differential expression in peripheral CD4$^+$ (higher) and CD8$^+$ T-cells (74) | 2082.21767 | 1.612003 |
| **TRANSPORTERS / ION CHANNELS / GAP JUNCTIONS** | | | | | |
| | ENSG00000092096 | SLC22A17 | iron transport, down-regulated in $T_{SCM}$ compared to $T_N$(75) | 52.2669151 | -4.621821 |
| | ENSG00000073150 | PANX2 | Structural component of the gap junctions and the hemichannels | 38.9963951 | -4.153572 |
| | ENSG00000136052 | SLC41A2 | Transport of glucose and other sugars, bile salts and organic acids, metal ions and amine compounds, induced by TNF (76) | 459.562097 | -2.864748 |
| | ENSG00000065923 | SLC9A7 | sodium and potassium/ proton antiporter, lower expression in non-human primate Ebola survivors than in non-survivors (77) | 1991.43475 | -1.612669 |
| | ENSG00000144935 | TRPC1 | needed for intracellular calcium increase by THC in T-cells via DAG (78) | 328.047316 | -2.158811 |
| | ENSG00000165474 | GJB2 | connexin 26, part of gap junctions | 36.2806533 | -3.677582 |
| | ENSG00000121742 | GJB6 | connexin, part of gap junction | 44.9584735 | -3.42367 |
| | ENSG00000075673 | ATP12A | $H^+/K^+$-ATPase | 231.196155 | -3.834959 |
| | ENSG00000185052 | SLC24A3 | Potassium-dependent sodium/calcium exchanger | 49.8386818 | 3.227841 |
| | ENSG00000122986 | HVCN1 | voltage-gated proton channel activity, downregulated in CD56$^+$ T-cells (able to kill CMV-infected cells), associated with metabolism (79) | 469.805016 | 0.929571 |
| | ENSG00000135750 | KCNK1 | contributes to passive transmembrane potassium transport | 36.3729301 | -2.730424 |
| | ENSG00000160190 | SLC37A1 | Pi-linked G6P antiporter (80) | 808.320349 | 1.500943 |
| | ENSG00000182747 | SLC35D3 | Solute Carrier | 12.9025324 | -2.956685 |
| | ENSG00000091137 | SLC26A4 | transmembrane transporter | 125.843343 | -2.113035 |
| | ENSG00000186187 | ZNRF1 | regulates the sodium/potassium pump (Na+/K+ATPase) with ZNRF2 (81), high expression in conventional T-cells in the lymph node, but not skin or fat (82) | 734.851294 | -1.470285 |
| | ENSG00000165449 | SLC16A9 | Proton-linked monocarboxylate transmembrane transporter | 14.7881211 | -2.481201 |

| | Ensemble ID | Gene | Function in T-cells | baseMean | log2Fold |
|---|---|---|---|---|---|
| **PROLIFERATION** | | | | | |
| | ENSG00000196507 | TCEAL3 | transcription elongation factor | 89.9871397 | -2.190882 |
| | ENSG00000175155 | YPEL2 | part of the mitotic apparatus required for cell division (83) | 1404.32806 | -1.179115 |
| | ENSG00000064886 | CHI3L2 | activation of ERK1/2 phosphorylation by CHI3L2 inhibits cell mitogenesis and proliferation (84) | 183.237313 | 1.318076 |
| | ENSG00000103202 | NME4 | involved in Pyrimidine metabolism and Metabolism | 325.668464 | -1.872898 |
| | ENSG00000174136 | RGMB | ligand for PD-L2, not increased upon T-cell activation and absent in naive mice, sets down the threshold for activation and supports T-cell expansion (67) | 181.825798 | -2.892547 |
| | ENSG00000127863 | TNFRSF19 | increases proliferation and NFkB signaling (68) | 52.2866821 | -2.607645 |
| | ENSG00000100867 | DHRS2 | cell cycle regulated NADPH-dependent dicarbonyl reductase enzyme (85) | 26.4152921 | -2.540096 |
| | ENSG00000174944 | P2RY14 | receptor for UDP-glucose, activation inhibits murine T-cell proliferation (86) | 83.7383867 | -2.181412 |
| | ENSG00000164362 | TERT | Telomerase Reverse Transcriptase, overexpression extends proliferative potential of human T-cells (9) | 27.7024907 | -3.430006 |
| | ENSG00000171860 | C3AR1 | activation by C3a enhances naive CD4$^+$ T-cell activation, survival and proliferation *via* PI3K/Akt (50) | 200.019164 | 2.171075 |
| **(CO-) STIMULATION** | | | | | |
| | ENSG00000101017 | CD40 | required for CD8 T-cells to receive help from CD4 T-cells and activation enhances CTL response (87) | 40.4430634 | -2.698722 |
| | ENSG00000114013 | CD86 | co-stimulation, upregulated on T-cells in Wegener's granulomatosis (generalized vasculitis) (88) | 1042.09351 | -2.500507 |
| | ENSG00000120949 | TNFRSF8 | CD30, upregulated in 15% of stimulated T-cells, enhanced by IL-2 (needs TCR stimulus + IL-2), CD45RO positive T-cells, cross-linking induces Ca2+ release (89) | 1077.91194 | -3.220663 |
| | ENSG00000197646 | PDCD1LG2 | PD1-L2, can confer co-stimulation (90) | 202.44762 | -1.595328 |
| | ENSG00000196372 | ASB13 | Class I MHC mediated antigen processing and presentation | 118.353047 | -2.247595 |
| | ENSG00000114455 | HHLA2 | undetectable on T-cells, binds to CD28H on T-cells (constitutively expressed on naive T-cells, lost in more differentiated memory T-cells) stimulates CD4 T-cell proliferation/increases cytokine production (91), inhibiting proliferation of TCR-stimulated T-cells/reduces cytokine production in T-cells (92) | 290.1457 | -2.415677 |
| | ENSG00000179583 | CIITA | induces expression of MHCII (93) | 1949.31925 | -0.996969 |
| **MIGRATION** | | | | | |
| | ENSG00000065534 | MYLK | myosin light chain kinase, role in ICAM-mediated cell migration (94) | 158.762531 | -2.480384 |
| | ENSG00000154928 | EPHB1 | recruits c-Src and p52Shc to activate MAPK/ERK and promote chemotaxis (95), ephrins stimulate T-cell chemotaxis (96) | 315.364149 | -2.743718 |
| | ENSG00000075391 | RASAL2 | activators of Ras superfamily of small GTPases, RET signaling and Cytoskeletal Signaling | 792.724493 | -2.702632 |
| | ENSG00000196526 | AFAP1 | modulator of actin filament integrity in response to cellular signals, upregulated in the absence of Itk which is downstream of TCR signalling (71) | 900.665659 | -1.728868 |
| | ENSG00000107719 | PALD1 | associated to actin,phosphorylation by Erk inhibits cell migration (97) | 43.9082093 | 2.758423 |
| | ENSG00000157827 | FMNL2 | involved in actin-dependenT-cell migration (98) | 143.44854 | -1.615625 |
| | ENSG00000079112 | CDH17 | calcium-dependenT-cell adhesion protein | 26.5219245 | 2.501369 |
| | ENSG00000172164 | SNTB1 | actin binding | 3080.4073 | 1.225121 |
| **ANERGY / EXHAUSTION** | | | | | |
| | ENSG00000111796 | KLRB1 | CD161, high expression marks anergy on CD8 T-cells failing to secrete cytokines, proliferate, kill (99) | 3183.52289 | 2.029115 |
| | ENSG00000137265 | IRF4 | induced by TCR signaling → upregulation of inhibitory receptor/impairs metabolism → T-cell exhaustion (25); induction dependent on the signal strength of TCR signaling → glycolysis/clonal expansion/maintenance of effector function (26) | 7024.41145 | -1.335874 |
| | ENSG00000204381 | LAYN | exhaustion marker (downregulates IFNg), reduced cancer-free survival, upregulated in CD8$^+$ T-cells and $T_{reg}$ upon CD3/28 stimulation (100); high expression in tumour infiltrating $T_{reg}$ correlates with poor prognosis; upregulated in $T_{RM}$ (101) | 87.4738512 | -2.672086 |
| | ENSG00000120708 | TGFBI | overexpressed in *post*-infusion *vs*. infusion of TCR engineered T-cells for melanoma (102) | 111.986209 | 2.881898 |
| | ENSG00000145246 | ATP10D | metabolism, upregulated in exhausted CAR T-cells (103) | 819.42255 | 1.292246 |
| **METABOLISM** | | | | | |
| | ENSG00000107518 | ATRNL1 | role in melanocortin signaling pathways that regulate energy homeostasis | 121.589335 | -3.432412 |
| | ENSG00000121039 | RDH10 | required for retinoic acid synthesis | 1368.99121 | -1.946465 |
| | ENSG00000143845 | ETNK2 | Metabolism and Glycerophospholipid biosynthesis | 115.851273 | -2.544206 |
| | ENSG00000164398 | ACSL6 | Fatty Acyl-CoA Biosynthesis | 628.820036 | 1.453343 |
| | ENSG00000016391 | CHDH | part of oxidative phosphorylation (104) | 946.804835 | -2.952135 |
| | ENSG00000103202 | NME4 | involved in Pyrimidine metabolism and Metabolism | 325.668464 | -1.872898 |
| | ENSG00000122986 | HVCN1 | voltage-gated proton channel activity, downregulated in CD56$^+$ T-cells (able to kill CMV-infected cells), associated with metabolism (79) | 469.805016 | 0.929571 |
| | ENSG00000116016 | EPAS1 | accumulates upon early TCR signaling and hypoxia, accumulation increases glycolysis/GZB, expression diminished upon administration of IL-2, increased upon administration of IL-4, sustained effector function of CD8 CTLs (12) | 3957.09145 | -1.554453 |
| **CELLULAR ORGANIZATION** | | | | | |
| | ENSG00000111052 | LIN7A | generating and maintaining the asymmetric distribution of channels and receptors at the cell membrane | 30.668353 | -3.962803 |
| | ENSG00000198932 | GPRASP1 | modulates lysosomal sorting and functional down-regulation of a variety of G-protein coupled receptors; variant linked to IPEX (105) | 311.376694 | -2.357607 |
| | ENSG00000168502 | MTCL1 | microtubule binding | 53.4993724 | 2.589775 |
| | ENSG00000161835 | GRASP | role in intracellular trafficking | 1140.15859 | -2.417493 |
| | ENSG00000128578 | STRIP2 | cell morphology and cytoskeletal organization | 778.705892 | -1.75502 |
| | ENSG00000080200 | CRYBG3 | mediates the subcellular compartmentation of protein kinase A | 1797.12246 | -1.542907 |
| | ENSG00000114745 | GORASP1 | establishes the stacked structure of the Golgi apparatus, upregulated in biopsies of acute cellular rejection with acute hepatitis (106) | 464.348531 | -1.190619 |
| | ENSG00000118200 | CAMSAP2 | microtubule binding | 77.1979364 | -2.558792 |
| **OTHERS** | | | | | |
| | ENSG00000104921 | FCER2 | B-cell specific antigen, and a low-affinity receptor for IgE | 70.5805133 | -5.282302 |
| | ENSG00000135919 | SERPINE2 | Thrombin, urokinase, plasmin and trypsin inhibitor | 304.039786 | 3.215362 |
| | ENSG00000123685 | BATF3 | on DCs needed for CD8 T-cell activation | 629.085812 | -3.290487 |
| | ENSG00000177383 | MAGEF1 | Enhances ubiquitin ligase activity of RING-type zinc finger-containing E3 ubiquitin ligases | 205.655947 | -1.802055 |
| | ENSG00000164742 | ADCY1 | regulated by calcium/calmodulin concentration, nucleotide binding and phosphorus-oxygen lyase activity | 154.296726 | -2.646613 |
| | ENSG00000205002 | AARD | Alanine And Arginine Rich Domain Containing Protein | 87.2130341 | -2.540349 |
| | ENSG00000281938 | CTB-127M13.1 | ??? | 599.808293 | 1.452842 |
| | ENSG00000166432 | ZMAT1 | target gene of p63 (107) | 171.866799 | -1.551626 |
| | ENSG00000020577 | SAMD4A | Acts as a translational repressor of SRE-containing messengers | 669.062337 | -1.860179 |
| | ENSG00000180044 | C3orf80 | ??? | 7.29341353 | -2.967343 |
| | ENSG00000183570 | PCBP3 | binds to RNA with a specificity for C-rich pyrimidine regions, post-transcriptional regulation (108), transcriptional repressor (109) | 206.493301 | -2.534946 |
| | ENSG00000043355 | ZIC2 | zinc-finger protein, regulates neurodevelopment | 56.9453982 | -3.618661 |
| | ENSG00000060566 | CREB3L3 | transcriptional regulator downstream of cAMP, acute phase response | 44.3122445 | -3.955702 |
| | ENSG00000169957 | ZNF768 | Zinc Finger | 187.373087 | -1.677774 |
| | ENSG00000102870 | ZNF629 | DNA binding transcription factor activity | 88.2902254 | -2.112764 |
| | ENSG00000173068 | BNC2 | transcription factor in Th22 cells (110) | 36.5735491 | 3.196431 |
| | ENSG00000166398 | KIAA0355 | ??? | 1864.69372 | -0.842476 |
| | ENSG00000115271 | GCA | calcium-binding on membrane and granules (111) | 189.326058 | -1.396011 |
| | ENSG00000087258 | GNAO1 | alpha subunit of the Go heterotrimeric G-protein signal-transducing complex | 180.918351 | 2.130565 |
| | ENSG00000147180 | ZNF711 | zinc finger protein | 74.2344179 | -2.445459 |
| | ENSG00000146416 | AIG1 | Androgen Induced 1 | 323.307154 | -1.18446 |
| | ENSG00000275342 | PRAG1 | tyrosine protein kinase | 192.233186 | 1.636761 |
| | ENSG00000111249 | CUX2 | RNA polymerase II regulatory region sequence-specific DNA binding | 28.5203944 | -2.487567 |
| | ENSG00000108239 | TBC1D12 | acts as a GTPase-activating protein for Rab family protein(s) | 83.3507848 | -2.131095 |
| | ENSG00000165138 | ANKS6 | ciliar protein | 336.962981 | -1.354375 |
| | ENSG00000111962 | UST | Uronyl 2-sulfotransferase | 847.243661 | -1.594802 |
| | ENSG00000112246 | SIM1 | DNA binding transcription factor activity and obsolete signal transducer activity | 219.600604 | -5.050657 |
| | ENSG00000145103 | ILDR1 | Ig-like receptor | 171.851598 | -4.569781 |

# References for Table 1


1. A. Mohandas, H. Zola, S. Barry and D. Krumbiegel: Peptidase inhibitor 16 identifies a unique subset of memory T helper cells with hyperproliferative and proinflammatory properties (IRC8P.477). *The Journal of Immunology*, 192(1 Supplement), 190.5 (2014)1. T.
2. Kondo and M. Takiguchi: Human memory CCR4+CD8+ T cell subset has the ability to produce multiple cytokines. *International Immunology*, 21(5), 523-532 (2009) doi:10.1093/intimm/dxp019
3. F. Sallusto, D. Lenig, R. Forster, M. Lipp and A. Lanzavecchia: Two subsets of memory T lymphocytes with distinct homing potentials and effector functions. *Nature*, 401(6754), 708-12 (1999) doi:10.1038/44385
4. Y. H. Wang, T. Ito, Y. H. Wang, B. Homey, N. Watanabe, R. Martin, C. J. Barnes, B. W. McIntyre, M. Gilliet, R. Kumar, Z. Yao and Y. J. Liu: Maintenance and polarization of human TH2 central memory T cells by thymic stromal lymphopoietin-activated dendritic cells. *Immunity*, 24(6), 827-38 (2006) doi:10.1016/j.immuni.2006.03.019
5. L. Lazuardi, D. Herndler-Brandstetter, S. Brunner, G. T. Laschober, G. Lepperdinger and B. Grubeck-Loebenstein: Microarray analysis reveals similarity between CD8+CD28− T cells from young and elderly persons, but not of CD8+CD28+ T cells. *Biogerontology*, 10(2), 191-202 (2009) doi:10.1007/s10522-008-9167-1
6. A. Railo, A. Pajunen, P. Itäranta, F. Naillat, J. Vuoristo, P. Kilpeläinen and S. Vainio: Genomic response to Wnt signalling is highly context-dependent — Evidence from DNA microarray and chromatin immunoprecipitation screens of Wnt/TCF targets. *Experimental Cell Research*, 315(16), 2690-2704 (2009) doi:https://doi.org/10.1016/j.yexcr.2009.06.021
7. L. Gattinoni, X. S. Zhong, D. C. Palmer, Y. Ji, C. S. Hinrichs, Z. Yu, C. Wrzesinski, A. Boni, L. Cassard, L. M. Garvin, C. M. Paulos, P. Muranski and N. P. Restifo: Wnt signaling arrests effector T cell differentiation and generates CD8+ memory stem cells. *Nat Med*, 15(7), 808-13 (2009) doi:10.1038/nm.1982
8. L. Yang, J. J. Kobie and T. R. Mosmann: CD73 and Ly-6A/E Distinguish In Vivo Primed but Uncommitted Mouse CD4 T Cells from Type 1 or Type 2 Effector Cells. *The Journal of Immunology*, 175(10), 6458 (2005)
9. N. Rufer, M. Migliaccio, J. Antonchuk, R. K. Humphries, E. Roosnek and P. M. Lansdorp: Transfer of the human telomerase reverse transcriptase (TERT) gene into T lymphocytes results in extension of replicative potential. *Blood*, 98(3), 597-603 (2001) doi:10.1182/blood.V98.3.597
10. S. Basu, T. Golovina, T. Mikheeva, C. H. June and J. L. Riley: Cutting Edge: Foxp3-Mediated Induction of Pim 2 Allows Human T Regulatory Cells to Preferentially Expand in Rapamycin. *The Journal of Immunology*, 180(9), 5794 (2008)
11. C. Riou, B. Yassine-Diab, J. Van grevenynghe, R. Somogyi, L. D. Greller, D. Gagnon, S. Gimmig, P. Wilkinson, Y. Shi, M. J. Cameron, R. Campos-Gonzalez, R. S. Balderas, D. Kelvin, R. P. Sekaly and E. K. Haddad: Convergence of TCR and cytokine signaling leads to FOXO3a phosphorylation and drives the survival of CD4+ central memory T cells. *J Exp Med*, 204(1), 79-91 (2007) doi:10.1084/jem.20061681
12. A. L. Doedens, A. T. Phan, M. H. Stradner, J. K. Fujimoto, J. V. Nguyen, E. Yang, R. S. Johnson and A. W. Goldrath: Hypoxia-inducible factors enhance the effector responses of CD8+ T cells to persistent antigen. *Nature Immunology*, 14, 1173 (2013) doi:10.1038/ni.2714
13. S. M. Kaech, J. T. Tan, E. J. Wherry, B. T. Konieczny, C. D. Surh and R. Ahmed: Selective expression of the interleukin 7 receptor identifies effector CD8 T cells that give rise to long-lived memory cells. *Nature Immunology*, 4, 1191 (2003) doi:10.1038/ni1009
14. Y. Pan, T. Tian, C. O. Park, S. Y. Lofftus, S. Mei, X. Liu, C. Luo, J. T. O'Malley, A. Gehad, J. E. Teague, S. J. Divito, R. Fuhlbrigge, P. Puigserver, J. G. Krueger, G. S. Hotamisligil, R. A. Clark and T. S. Kupper: Survival of tissue-resident memory T cells requires exogenous lipid uptake and metabolism. *Nature*, 543, 252 (2017) doi:10.1038/nature21
15. J. Wherry, S.-J. Ha, S. M. Kaech, W. N. Haining, S. Sarkar, V. Kalia, S. Subramaniam, J. N. Blattman, D. L. Barber and R. Ahmed: Molecular Signature of CD8+ T Cell Exhaustion during Chronic Viral Infection. *Immunity*, 27(4), 670-684 (2007) doi:https://doi.org/10.1016/j.immuni.2007.09.006
16. Y. Araki, Z. Wang, C. Zang, W. H. Wood, D. Schones, K. Cui, T.-Y. Roh, B. Lhotsky, R. P. Wersto, W. Peng, K. G. Becker, K. Zhao and N.-p. Weng: Genome-wide Analysis of Histone Methylation Reveals Chromatin State-Based Regulation of Gene Transcription and Function of Memory CD8+ T Cells. *Immunity*, 30(6), 912-925 (2009) doi:https://doi.org/10.1016/j.immuni.2009.05.006
17. R. S. Douglas, A. G. Gianoukakis, S. Kamat and T. J. Smith: Aberrant Expression of the Insulin-Like Growth Factor-1 Receptor by T Cells from Patients with Graves' Disease May Carry Functional Consequences for Disease Pathogenesis. *The Journal of Immunology*, 178(5), 3281 (2007)
18. H. K. Komori, T. Hart, S. A. LaMere, P. V. Chew and D. R. Salomon: Defining CD4 T Cell Memory by the Epigenetic Landscape of CpG DNA Methylation. *The Journal of Immunology* (2015)
19. M. Zhao, S. Liu, S. Luo, H. Wu, M. Tang, W. Cheng, Q. Zhang, P. Zhang, X. Yu, Y. Xia, N. Yi, F. Gao, L. Wang, S. Yung, T. M. Chan, A. H. Sawalha, B. Richardson, M. E. Gershwin, N. Li and Q. Lu: DNA methylation and mRNA and microRNA expression of SLE CD4+ T cells correlate with disease phenotype. *Journal of Autoimmunity*, 54, 127-136 (2014) doi:https://doi.org/10.1016/j.jaut.2014.07.002
20. A. Miyake, Y. Takahashi, H. Miwa, A. Shimada, M. Konishi and N. Itoh: Neucrin is a novel neural-specific secreted antagonist to canonical Wnt signaling. *Biochemical and Biophysical Research Communications*, 390(3), 1051-1055 (2009) doi:https://doi.org/10.1016/j.bbrc.2009.10.113
21. R. L. Hengel, V. Thaker, M. V. Pavlick, J. A. Metcalf, G. Dennis, J. Yang, R. A. Lempicki, I. Sereti and H. C. Lane: Cutting Edge: L-Selectin (CD62L) Expression Distinguishes Small Resting Memory CD4<sup>+</sup> T Cells That Preferentially Respond to Recall Antigen. *The Journal of Immunology*, 170(1), 28 (2003)
22. M.-L. Chen, T.-C. Tsai, Y.-Y. Lin, Y.-M. Tsai, L.-K. Wang, M.-C. Lee and F.-M. Tsai: Antipsychotic drugs suppress the AKT/NF-κB pathway and regulate the differentiation of T-cell subsets. *Immunology Letters*, 140(1), 81-91 (2011) doi:https://doi.org/10.1016/j.imlet.2011.06.011
23. J. Ashton-Chess, M. Giral, M. Mengel, K. Renaudin, Y. Foucher, W. Gwinner, C. Braud, E. Dugast, T. Quillard, P. Thebault, E. Chiffoleau, C. Braudeau, B. Charreau, J.-P. Soulillou and S. Brouard: Tribbles-1 as a Novel Biomarker of Chronic Antibody-Mediated Rejection. *Journal of the American Society of Nephrology*, 19(6), 1116-1127 (2008)
24. R. Bhairavabhotla, Y. C. Kim, D. D. Glass, T. M. Escobar, M. C. Patel, R. Zahr, C. K. Nguyen, G. K. Kilaru, S. A. Muljo and E. M. Shevach: Transcriptome profiling of human FoxP3+ regulatory T cells. *Human Immunology*, 77(2), 201-213 (2016) doi:https://doi.org/10.1016/j.humimm.2015.12.004
25. K. Man, M. Miasari, W. Shi, A. Xin, D. C. Henstridge, S. Preston, M. Pellegrini, G. T. Belz, G. K. Smyth, M. A. Febbraio, S. L. Nutt and A. Kallies: The transcription factor IRF4 is essential for TCR affinity–mediated metabolic programming and clonal expansion of T cells. *Nature Immunology*, 14, 1155 (2013) doi:10.1038/ni.2710 https://www.nature.com/articles/ni.2710#supplementary-information
26. K. Man, S. S. Gabriel, Y. Liao, R. Gloury, S. Preston, D. C. Henstridge, M. Pellegrini, D. Zehn, F. Berberich-Siebelt, M. A. Febbraio, W. Shi and A. Kallies: Transcription Factor IRF4 Promotes CD8(+) T Cell Exhaustion and Limits the Development of Memory-like T Cells during Chronic Infection. *Immunity*, 47(6), 1129-1141 e5 (2017) doi:10.1016/j.immuni.2017.11.021
27. J. LeMaoult, K. Zafaranloo, C. Le Danff and E. D. Carosella: HLA-G up-regulates ILT2, ILT3, ILT4, and KIR2DL4 in antigen presenting cells, NK cells, and T cells. *The FASEB Journal*, 19(6), 662-664 (2005) doi:10.1096/fj.04-1617fje
28. G. Li, C. M. Weyand and J. J. Goronzy: Epigenetic mechanisms of age-dependent KIR2DL4 expression in T cells. *Journal of Leukocyte Biology*, 84(3), 824-834 (2008) doi:10.1189/jlb.0807583
29. S.-i. Hashimoto, K. Ogoshi, A. Sasaki, J. Abe, W. Qu, Y. Nakatani, B. Ahsan, K. Oshima, F. H. W. Shand, A. Ametani, Y. Suzuki, S. Kaneko, T. Wada, M. Hattori, S. Sugano, S. Morishita and K. Matsushima: Coordinated Changes in DNA Methylation in Antigen-Specific Memory CD4 T Cells. *The Journal of Immunology* (2013)
30. M. Franceschetti, A. Pievani, G. Borleri, L. Vago, K. Fleischhauer, J. Golay and M. Introna: Cytokine-induced killer cells are terminallydifferentiated activated CD8 cytotoxic T-EMRA lymphocytes. *Experimental Hematology*, 37(5), 616-628.e2 (2009) doi:https://doi.org/10.1016/j.exphem.2009.01.010
31. S. Krishnan, V. G. Warke, M. P. Nambiar, G. C. Tsokos and D. L. Farber: The FcRγ Subunit and Syk Kinase Replace the CD3ζ-Chain and ZAP-70 Kinase in the TCR Signaling Complex of Human Effector CD4 T Cells. *The Journal of Immunology*, 170(8), 4189 (2003)
32. M. Okamoto, M. Van Stry, L. Chung, M. Koyanagi, X. Sun, Y. Suzuki, O. Ohara, H. Kitamura, A. Hijikata, M. Kubo and M. Bix: MINA, an IL-4 repressor controls T(H)2 bias. *Nature immunology*, 10(8), 872-879 (2009) doi:10.1038/ni.1747
33. E. Larrea, R. Aldabe, I. Gonzalez, V. Segura, P. Sarobe, I. Echeverria and J. Prieto: Oncostatin M Enhances the Antiviral Effects of Type I Interferon and Activates Immunostimulatory Functions in Liver Epithelial Cells. *Journal of Virology*, 83(7), 3298 (2009)
34. K. Boniface, C. Diveu, F. Morel, N. Pedretti, J. Froger, E. Ravon, M. Garcia, E. Venereau, L. Preisser, E. Guignouard, G. Guillet, G. Dagregorio, J. Pène, J.-P. Moles, H. Yssel, S. Chevalier, F.-X. Bernard, H. Gascan and J.-C. Lecron: Oncostatin M Secreted by Skin Infiltrating T Lymphocytes Is a Potent Keratinocyte Activator Involved in Skin Inflammation. *The Journal of Immunology*, 178(7), 4615 (2007)
35. M. Beyer, Y. Thabet, R.-U. Müller, T. Sadlon, S. Classen, K. Lahl, S. Basu, X. Zhou, S. L. Bailey-Bucktrout, W. Krebs, E. A. Schonfeld, J. Bottcher, T. Golovina, C. T. Mayer, A. Hofmann, D. Sommer, S. Debey-Pascher, E. Endl, A. Limmer, K. L. Hippen, B. R. Blazar, R. Balderas, T. Quast, A. Waha, G. Mayer, M. Famulok, P. A. Knolle, C. Wickenhauser, W. Kolanus, B. Schermer, J. A. Bluestone, S. C. Barry, T. Sparwasser, J. L. Riley and J. L. Schultze: Repression of the genome organizer SATB1 in regulatory T cells is required for suppressive function and inhibition of effector differentiation(1529-2916 (Electronic)) (2011)
36. J. Choi, S. Djebbar, A. Fournier and C. Labrie: The co-chaperone DNAJC12 binds to Hsc70 and is upregulated by endoplasmic reticulum stress. *Cell Stress and Chaperones*, 19(3), 439-446 (2014) doi:10.1007/s12192-013-0471-6
37. C. Figueiredo, M. Wittmann, D. Wang, R. Dressel, A. Seltsam, R. Blasczyk and B. Eiz-Vesper: Heat shock protein 70 (HSP70) induces cytotoxicity of T-helper cells. *Blood*, 113(13), 3008 (2009)
38. A. Minty, P. Chalon, J. M. Derocq, X. Dumont, J. C. Guillemot, M. Kaghad, C. Labit, P. Leplatois, P. Liauzun, B. Miloux, C. Minty, P. Casellas, G. Loison, J. Lupker, D. Shire, P. Ferrara and D. Caput: Interleukin-13 is a new human lymphokine regulating inflammatory and immune responses. *Nature*, 362, 248 (1993) doi:10.1038/362248a0
39. D. M. Anderson, E. Maraskovsky, W. L. Billingsley, W. C. Dougall, M. E. Tometsko, E. R. Roux, M. C. Teepe, R. F. DuBose, D. Cosman and L. Galibert: A homologue of the TNF receptor and its ligand enhance T-cell growth and dendritic-cell function. *Nature*, 390(6656), 175-9 (1997) doi:10.1038/36593
40. N.-J. Chen, M.-W. Huang and S.-L. Hsieh: Enhanced Secretion of IFN-γ by Activated Th1 Cells Occurs Via Reverse Signaling Through TNF-Related Activation-Induced Cytokine. *The Journal of Immunology*, 166(1), 270 (2001)
41. Z. Chen, E. Stelekati, M. Kurachi, S. Yu, Z. Cai, S. Manne, O. Khan, X. Yang and E. J. Wherry: miR-150 Regulates Memory CD8 T Cell Differentiation via c-Myb. *Cell Rep*, 20(11), 2584-2597 (2017) doi:10.1016/j.celrep.2017.08.060
42. B. J. Belin, T. Lee and R. D. Mullins: DNA damage induces nuclear actin filament assembly by Formin -2 and Spire-(1/2) that promotes efficient DNA repair. [corrected]. *Elife*, 4, e07735 (2015) doi:10.7554/eLife.07735
43. M. Vantler, M. Huntgeburth, E. Caglayan, H. Ten Freyhaus, P. Schnabel and S. Rosenkranz: PI3-kinase/Akt-dependent antiapoptotic signaling by the PDGF alpha receptor is negatively regulated by Src family kinases. *FEBS Lett*, 580(30), 6769-76 (2006) doi:10.1016/j.febslet.2006.11.034
44. Y. Rochman, R. Spolski and W. J. Leonard: New insights into the regulation of T cells by γc family cytokines. *Nature Reviews Immunology*, 9, 480 (2009) doi:10.1038/nri2580
45. K. Behbakht, L. Qamar, C. S. Aldridge, R. D. Coletta, S. A. Davidson, A. Thorburn and H. L. Ford: Six1 Overexpression in Ovarian Carcinoma Causes Resistance to TRAIL-Mediated Apoptosis and Is Associated with Poor Survival. *Cancer Research*, 67(7), 3036 (2007)
46. J. W. Rocco, C.-O. Leong, N. Kuperwasser, M. P. DeYoung and L. W. Ellisen: p63 mediates survival in squamous cell carcinoma by suppression of p73-dependent apoptosis. *Cancer Cell*, 9(1), 45-56 (2006) doi:https://doi.org/10.1016/j.ccr.2005.12.013
47. M. T. Epping, L. A. T. Meijer, O. Krijgsman, J. L. Bos, P. P. Pandolfi and R. Bernards: TSPYL5 suppresses p53 levels and function by physical interaction with USP7. *Nature Cell Biology*, 13, 102 (2010) doi:10.1038/ncb2142 https://www.nature.com/articles/ncb2142#supplementary-information
48. A. Naderi, J. Liu and I. C. Bennett: BEX2 regulates mitochondrial apoptosis and G1 cell cycle in breast cancer. *International Journal of Cancer*, 126(7), 1596-1610 (2009) doi:10.1002/ijc.24866
49. K.-H. Kim, Y.-W. Kim, H. B. Kim, B. J. Lee and D. S. Lee: Anti-apoptotic Activity of Laminarin Polysaccharides and their Enzymatically Hydrolyzed Oligosaccharides from Laminaria japonica. *Biotechnology Letters*, 28(6), 439-446 (2006) doi:10.1007/s10529-005-6177-9
50. M. G. Strainic, J. Liu, D. Huang, F. An, P. N. Lalli, N. Muqim, V. S. Shapiro, George R. Dubyak, P. S. Heeger and M. E. Medof: Locally Produced Complement Fragments C5a and C3a Provide Both Costimulatory and Survival Signals to Naive CD4+ T Cells. *Immunity*, 28(3), 425-435 (2008) doi:https://doi.org/10.1016/j.immuni.2008.02.001
51. Y. Jin, E. K. Blue and P. J. Gallagher: Control of death-associated protein kinase (DAPK) activity by phosphorylation and proteasomal degradation. *J Biol Chem*, 281(51), 39033-40 (2006) doi:10.1074/jbc.M605097200
52. T. Fernandes-Alnemri, J.-W. Yu, P. Datta, J. Wu and E. S. Alnemri: AIM2 activates the inflammasome and cell death in response to cytoplasmic DNA. *Nature*, 458, 509 (2009) doi:10.1038/nature07710 https://www.nature.com/articles/nature07710#supplementary-information
53. M. Wang, D. Windgassen and E. T. Papoutsakis: Comparative analysis of transcriptional profiling of CD3+, CD4+ and CD8+ T cells identifies novel immune response players in T-Cell activation. *BMC Genomics*, 9(1), 225 (2008) doi:10.1186/1471-2164-9-225
54. M. Locci, J. E. Wu, F. Arumemi, Z. Mikulski, C. Dahlberg, A. T. Miller and S. Crotty: Activin A programs the differentiation of human TFH cells. *Nature Immunology*, 17, 976 (2016) doi:10.1038/ni.3494 https://www.nature.com/articles/ni.3494#supplementary-information
55. H. Sandig, J. McDonald, J. Gilmour, M. Arno, T. H. Lee and D. J. Cousins: Human Th2 cells selectively express the orexigenic peptide, pro-melanin-concentrating hormone. *Proceedings of the National Academy of Sciences*, 104(30), 12440 (2007)
56. I. Prots, A. Skapenko, P. E. Lipsky and H. Schulze-Koops: Analysis of the Transcriptional Program of Developing Induced Regulatory T Cells. *PLOS ONE*, 6(2), e16913 (2011) doi:10.1371/journal.pone.0016913
57. Y. Liu, W. Dong, L. Chen, R. Xiang, H. Xiao, G. De, Z. Wang and Y. Qi: BCL10 mediates lipopolysaccharide/toll-like receptor-4 signaling through interaction with Pellino2. *J Biol Chem*, 279(36), 37436-44 (2004) doi:10.1074/jbc.M400241200
58. V. Kalia, S. Sarkar, S. Subramaniam, W. N. Haining, K. A. Smith and R. Ahmed: Prolonged Interleukin-2Rα Expression on Virus-Specific CD8+ T Cells Favors Terminal-Effector Differentiation In Vivo. *Immunity*, 32(1), 91-103 (2010) doi:https://doi.org/10.1016/j.immuni.2009.11.010
59. F. Larousserie, E. Bardel, S. Pflanz, B. Arnulf, C. Lome-Maldonado, O. Hermine, L. Brégeaud, M. Perennec, N. Brousse, R. Kastelein and O. Devergne: Analysis of Interleukin-27 (EBI3/p28) Expression in Epstein-Barr Virus- and Human T-Cell Leukemia Virus Type 1-Associated Lymphomas. *The American Journal of Pathology*, 166(4), 1217-1228 (2005) doi:10.1016/s0002-9440(10)62340-1
60. J. A. Best, D. A. Blair, J. Knell, E. Yang, V. Mayya, A. Doedens, M. L. Dustin, A. W. Goldrath, C. The Immunological Genome Project, P. Monach, S. A. Shinton, R. R. Hardy, R. Jianu, D. Koller, J. Collins, R. Gazit, B. S. Garrison, D. J. Rossi, K. Narayan, K. Sylvia, J. Kang, A. Fletcher, K. Elpek, A. Bellemare-Pelletier, D. Malhotra, S. Turley, J. A. Best, J. Knell, A. W. Goldrath, V. Jojic, D. Koller, T. Shay, A. Regev, N. Cohen, P. Brennan, M. Brenner, T. Kreslavsky, N. A. Bezman, J. C. Sun, C. C. Kim, L. L. Lanier, J. Miller, B. Brown, M. Merad, E. L. Gautier, C. Jakubzick, G. J. Randolph, F. Kim, T. N. Rao, A. Wagers, T. Heng, M. Painter, J. Ericson, S. Davis, A. Ergun, M. Mingueneau, D. Mathis and C. Benoist: Transcriptional insights into the CD8+ T cell response to infection and memory T cell formation. *Nature Immunology*, 14, 404 (2013) doi:10.1038/ni.2536 https://www.nature.com/articles/ni.2536#supplementary-information
61. M. Thessen Hedreul, S. Möller, P. Stridh, Y. Gupta, A. Gillett, A. Daniel Beyeen, J. Öckinger, S. Flytzani, M. Diez, T. Olsson and M. Jagodic: Combining genetic mapping with genome-wide expression in experimental autoimmune encephalomyelitis highlights a gene network enriched for T cell functions and candidate genes regulating autoimmunity. *Human Molecular Genetics*, 22(24), 4952-4966 (2013) doi:10.1093/hmg/ddt343
62. International Multiple Sclerosis Genetics Consortium, A. H. Beecham, N. A. Patsopoulos, D. K. Xifara, M. F. Davis, A. Kemppinen, C. Cotsapas, T. S. Shah, C. Spencer, D. Booth, A. Goris, A. Oturai, J. Saarela, B. Fontaine, B. Hemmer, C. Martin, F. Zipp, S. D'Alfonso, F. Martinelli-Boneschi, B. Taylor, H. F. Harbo, I. Kockum, J. Hillert, T. Olsson, M. Ban, J. R. Oksenberg, R. Hintzen, L. F. Barcellos, C. Agliardi, L. Alfredsson, M. Alizadeh, C. Anderson, R. Andrews, H. B. Søndergaard, A. Baker, G. Band, S. E. Baranzini, N. Barizzone, J. Barrett, C. Bellenguez, L. Bergamaschi, L. Bernardinelli, A. Berthele, V. Biberacher, T. M. C. Binder, H. Blackburn, I. L. Bomfim, P. Brambilla, S. Broadley, B. Brochet, L. Brundin, D. Buck, H. Butzkueven, S. J. Caillier, W. Camu, W. Carpentier, P. Cavalla, E. G. Celius, I. Coman, G. Comi, L. Corrado, L. Cosemans, I. Cournu-Rebeix, B. A. C. Cree, D. Cusi, V. Damotte, G. Defer, S. R. Delgado, P. Deloukas, A. di Sapio, A. T. Dilthey, P. Donnelly, B. Dubois, M. Duddy, S. Edkins, I. Elovaara, F. Esposito, N. Evangelou, B. Fiddes, J. Field, A. Franke, C. Freeman, I. Y. Frohlich, D. Galimberti, C. Gieger, P.-A. Gourraud, C. Graetz, A. Graham, V. Grummel, C. Guaschino, A. Hadjixenofontos, H. Hakonarson, C. Halfpenny, G. Hall, P. Hall, A. Hamsten, J. Harley, T. Harrower, C. Hawkins, G. Hellenthal, C. Hillier, J. Hobart, M. Hoshi, S. E. Hunt, M. Jagodic, I. Jelčić, A. Jochim, B. Kendall, A. Kermode, T. Kilpatrick, K. Koivisto, I. Konidari, T. Korn, H. Kronsbein, C. Langford, M. Larsson, M. Lathrop, C. Lebrun-Frenay, J. Lechner-Scott, M. H. Lee, M. A. Leone, V. Leppä, G. Liberatore, B. A. Lie, C. M. Lill, M. Lindén, J. Link, F. Luessi, J. Lycke, F. Macciardi, S. Männistö, C. P. Manrique, R. Martin, V. Martinelli, D. Mason, G. Mazibrada, C. McCabe, I.-L. Mero, J. Mescheriakova, L. Moutsianas, K.-M. Myhr, G. Nagels, R. Nicholas, P. Nilsson, F. Piehl, M. Pirinen, S. E. Price, H. Quach, M. Reunanen, W. Robberecht, N. P. Robertson, M. Rodegher, D. Rog, M. Salvetti, N. C. Schnetz-Boutaud, F. Sellebjerg, R. C. Selter, C. Schaefer, S. Shaunak, L. Shen, S. Shields, V. Siffrin, M. Slee, P. S. Sorensen, M. Sorosina, M. Sospedra, A. Spurkland, A. Strange, E. Sundqvist, V. Thijs, J. Thorpe, A. Ticca, P. Tienari, C. van Duijn, E. M. Visser, S. Vucic, H. Westerlind, J. S. Wiley, A. Wilkins, J. F. Wilson, J. Winkelmann, J. Zajicek, E. Zindler, J. L. Haines, M. A. Pericak-Vance, A. J. Ivinson, G. Stewart, D. Hafler, S. L. Hauser, A. Compston, G. McVean, P. De Jager, S. J. Sawcer and J. L. McCauley: Analysis of immune-related loci identifies 48 new susceptibility variants for multiple sclerosis. *Nature Genetics*, 45, 1353 (2013) doi:10.1038/ng.2770 https://www.nature.com/articles/ng.2770#supplementary-information
63. J. T. Chang, E. M. Shevach and B. M. Segal: Regulation of Interleukin (IL)-12 Receptor β2 Subunit Expression by Endogenous IL-12: A Critical Step in the Differentiation of Pathogenic Autoreactive T Cells. *The Journal of Experimental Medicine*, 189(6), 969 (1999)
64. N. Takemoto, A. M. Intlekofer, J. T. Northrup, E. J. Wherry and S. L. Reiner: Cutting Edge: IL-12 inversely regulates T-bet and eomesodermin expression during pathogen-induced CD8+ T cell differentiation(0022-1767 (Print)) (2006)
65. J. Geng, S. Yu, H. Zhao, X. Sun, X. Li, P. Wang, X. Xiong, L. Hong, C. Xie, J. Gao, Y. Shi, J. Peng, R. L. Johnson, N. Xiao, L. Lu, J. Han, D. Zhou and L. Chen: The transcriptional coactivator TAZ regulates reciprocal differentiation of TH17 cells and Treg cells. *Nature Immunology*, 18, 800 (2017) doi:10.1038/ni.3748 https://www.nature.com/articles/ni.3748#supplementary-information
66. N. Shioda, S. Moriguchi, Y. Shirasaki and K. Fukunaga: Generation of constitutively active calcineurin by calpain contributes to delayed neuronal death following mouse brain ischemia. *J Neurochem*, 98(1), 310-20 (2006) doi:10.1111/j.1471-4159.2006.03874.x
67. Y. Xiao, S. Yu, B. Zhu, D. Bedoret, X. Bu, L. M. Francisco, P. Hua, J. S. Duke-Cohan, D. T. Umetsu, A. H. Sharpe, R. H. DeKruyff and G. J. Freeman: RGMb is a novel binding partner for PD-L2 and its engagement with PD-L2 promotes respiratory tolerance. *The Journal of Experimental Medicine*, 211(5), 943 (2014)
68. U. Gaur and B. B. Aggarwal: Regulation of proliferation, survival and apoptosis by members of the TNF superfamily. *Biochemical Pharmacology*, 66(8), 1403-1408 (2003) doi:https://doi.org/10.1016/S0006-2952(03)00490-8
69. X. Huang, Y. Li, K. Tanaka, K. G. Moore and J. I. Hayashi: Cloning and characterization of Lnk, a signal transduction protein that links T-cell receptor activation signal to phospholipase C gamma 1, Grb2, and phosphatidylinositol 3-kinase. *Proceedings of the National Academy of Sciences*, 92(25), 11618 (1995)
70. M. T. Cencioni, V. Chiurchiu, G. Catanzaro, G. Borsellino, G. Bernardi, L. Battistini and M. Maccarrone: Anandamide suppresses proliferation and cytokine release from primary human T-lymphocytes mainly via CB2 receptors. *PLoS One*, 5(1), e8688 (2010) doi:10.1371/journal.pone.0008688
71. K. E. M. Blomberg, N. Boucheron, J. M. Lindvall, L. Yu, J. Raberger, A. Berglöf, W. Ellmeier and C. I. E. Smith: Transcriptional signatures of Itk-deficient CD3+, CD4+ and CD8+ T-cells. *BMC Genomics*, 10(1), 233 (2009) doi:10.1186/1471-2164-10-233
72. A. Mikhailik, B. Ford, J. Keller, Y. Chen, N. Nassar and N. Carpino: A Phosphatase Activity of Sts-1 Contributes to the Suppression of TCR Signaling. *Molecular Cell*, 27(3), 486-497 (2007) doi:https://doi.org/10.1016/j.molcel.2007.06.015
73. T. T. Zhang, H. Li, S. M. Cheung, J. L. Costantini, S. Hou, M. Al-Alwan and A. J. Marshall: Phosphoinositide 3-kinase-regulated adapters in lymphocyte activation. *Immunol Rev*, 232(1), 255-72 (2009) doi:10.1111/j.1600-065X.2009.00838.x
74. M. Mingueneau, T. Kreslavsky, D. Gray, T. Heng, R. Cruse, J. Ericson, S. Bendall, M. H. Spitzer, G. P. Nolan, K. Kobayashi, H. von Boehmer, D. Mathis, C. Benoist, C. the Immunological Genome, A. J. Best, J. Knell, A. Goldrath, V. Jojic, D. Koller, T. Shay, A. Regev, N. Cohen, P. Brennan, M. Brenner, F. Kim, T. N. Rao, A. Wagers, T. Heng, J. Ericson, K. Rothamel, A. Ortiz-Lopez, D. Mathis, C. Benoist, N. A. Bezman, J. C. Sun, G. Min-Oo, C. C. Kim, L. L. Lanier, J. Miller, B. Brown, M. Merad, E. L. Gautier, C. Jakubzick, G. J. Randolph, P. Monach, D. A. Blair, M. L. Dustin, S. A. Shinton, R. R. Hardy, D. Laidlaw, J. Collins, R. Gazit, D. J. Rossi, N. Malhotra, K. Sylvia, J. Kang, T. Kreslavsky, A. Fletcher, K. Elpek, A. Bellemare-Pelletier, D. Malhotra and S. Turley: The transcriptional landscape of αβ T cell differentiation. *Nature Immunology*, 14, 619 (2013) doi:10.1038/ni.2590 https://www.nature.com/articles/ni.2590#supplementary-information
75. G. Scholz, C. Jandus, L. Zhang, C. Grandclement, I. C. Lopez-Mejia, C. Soneson, M. Delorenzi, L. Fajas, W. Held, O. Dormond and P. Romero: Modulation of mTOR Signalling Triggers the Formation of Stem Cell-like Memory T Cells. *EBioMedicine*, 4, 50-61 (2016) doi:10.1016/j.ebiom.2016.01.019
76. M. Nagar, J. Jacob-Hirsch, H. Vernitsky, Y. Berkun, S. Ben-Horin, N. Amariglio, I. Bank, Y. Kloog, G. Rechavi and I. Goldstein: TNF activates a NF-kappaB-regulated cellular program in human CD45RA- regulatory T cells that modulates their suppressive function. *J Immunol*, 184(7), 3570-81 (2010) doi:10.4049/jimmunol.0902070
77. S. Garamszegi, J. Y. Yen, A. N. Honko, J. B. Geisbert, K. H. Rubins, T. W. Geisbert, Y. Xia, L. E. Hensley and J. H. Connor: Transcriptional correlates of disease outcome in anticoagulant-treated non-human primates infected with ebolavirus. *PLoS Negl Trop Dis*, 8(7), e3061 (2014) doi:10.1371/journal.pntd.0003061
78. G. K. Rao and N. E. Kaminski: Induction of intracellular calcium elevation by Δ9-tetrahydrocannabinol in T cells involves TRPC1 channels. *Journal of Leukocyte Biology*, 79(1), 202-213 (2005) doi:10.1189/jlb.0505274
79. W. K. Chan, P. Rujkijyanont, G. Neale, J. Yang, R. Bari, N. Das Gupta, M. Holladay, B. Rooney and W. Leung: Multiplex and Genome-Wide Analyses Reveal Distinctive Properties of KIR<sup>+</sup> and CD56<sup>+</sup> T Cells in Human Blood. *The Journal of Immunology* (2013)
80. J. Y. Chou, H. Sik Jun and B. C. Mansfield: The SLC37 family of phosphate-linked sugar phosphate antiporters. *Molecular Aspects of Medicine*, 34(2), 601-611 (2013) doi:https://doi.org/10.1016/j.mam.2012.05.010
81. G. Hoxhaj, A. Najafov, R. Toth, D. G. Campbell, A. R. Prescott and C. MacKintosh: ZNRF2 is released from membranes by growth factors and with ZNRF1 regulates the Na<sup>+</sup>/K<sup>+</sup>ATPase. *Journal of Cell Science* (2012)
82. M. Delacher, C. D. Imbusch, D. Weichenhan, A. Breiling, A. Hotz-Wagenblatt, U. Träger, A.-C. Hofer, D. Kägebein, Q. Wang, F. Frauhammer, J.-P. Mallm, K. Bauer, C. Herrmann, P. A. Lang, B. Brors, C. Plass and M. Feuerer: Genome-wide DNA-methylation landscape defines specialization of regulatory T cells in tissues. *Nature Immunology*, 18, 1160 (2017) doi:10.1038/ni.3799 https://www.nature.com/articles/ni.3799#supplementary-information
83. K. Hosono, T. Sasaki, S. Minoshima and N. Shimizu: Identification and characterization of a novel gene family YPEL in a wide spectrum of eukaryotic species. *Gene*, 340(1), 31-43 (2004) doi:https://doi.org/10.1016/j.gene.2004.06.014
84. P. O. Areshkov, S. S. Avdieiev, O. V. Balynska, D. LeRoith and V. M. Kavsan: Two Closely Related Human Members of Chitinase-like Family, CHI3L1 and CHI3L2, Activate ERK1/2 in 293 and U373 Cells but Have the Different Influence on Cell Proliferation. *International Journal of Biological Sciences*, 8(1), 39-48 (2012) doi:10.7150/ijbs.8.39
85. F. Gabrielli and S. Tofanelli: Molecular and functional evolution of human DHRS2 and DHRS4 duplicated genes. *Gene*, 511(2), 461-469 (2012) doi:https://doi.org/10.1016/j.gene.2012.09.013
86. M. Scrivens and J. M. Dickenson: Functional expression of the P2Y(14) receptor in murine T-lymphocytes. *British Journal of Pharmacology*, 146(3), 435-444 (2005) doi:10.1038/sj.bjp.0706322
87. M. A. Ostrowski, S. J. Justement, L. Ehler, S. B. Mizell, S. Lui, J. Mican, B. D. Walker, E. K. Thomas, R. Seder and A. S. Fauci: The Role of CD4+ T Cell Help and CD40 Ligand in the In Vitro Expansion of HIV-1-Specific Memory Cytotoxic CD8+ T Cell Responses. *The Journal of Immunology*, 165(11), 6133 (2000)
88. F. Moosig, E. Csernok, G. Wang and W. L. Gross: Costimulatory molecules in Wegener's granulomatosis (WG): lack of expression of CD28 and preferential up-regulation of its ligands B7-1 (CD80) and B7-2 (CD86) on T cells. *Clinical and Experimental Immunology*, 114(1), 113-118 (1998) doi:10.1046/j.1365-2249.1998.00695.x
89. T. M. Ellis, P. E. Simms, D. J. Slivnick, H. M. Jäck and R. I. Fisher: CD30 is a signal-transducing molecule that defines a subset of human activated CD45RO+ T cells. *The Journal of Immunology*, 151(5), 2380 (1993)
90. S. Wang, J. Bajorath, D. B. Flies, H. Dong, T. Honjo and L. Chen: Molecular Modeling and Functional Mapping of B7-H1 and B7-DC Uncouple Costimulatory Function from PD-1 Interaction. *The Journal of Experimental Medicine*, 197(9), 1083 (2003)
91. Y. Zhu, S. Yao, B. P. Iliopoulou, X. Han, M. M. Augustine, H. Xu, R. T. Phennicie, S. J. Flies, M. Broadwater, W. Ruff, J. M. Taube, L. Zheng, L. Luo, G. Zhu, J. Chen and L. Chen: B7-H5 costimulates human T cells via CD28H. *Nature communications*, 4, 2043-2043 (2013) doi:10.1038/ncomms3043
92. R. Zhao, J. M. Chinai, S. Buhl, L. Scandiuzzi, A. Ray, H. Jeon, K. C. Ohaegbulam, K. Ghosh, A. Zhao, M. D. Scharff and X. Zang: HHLA2 is a member of the B7 family and inhibits human CD4 and CD8 T-cell function. *Proceedings of the National Academy of Sciences* (2013)
93. C.-H. Chang, S.-C. Hong, C. C. W. Hughes, J. C. A. Janeway and R. A. Flavell: CIITA activates the expression of MHC class II genes in mouse T cells. *International Immunology*, 7(9), 1515-1518 (1995) doi:10.1093/intimm/7.9.1515
94. A. Smith, M. Bracke, B. Leitinger, J. C. Porter and N. Hogg: LFA-1-induced T cell migration on ICAM-1 involves regulation of MLCK-mediated attachment and ROCK-dependent detachment. *Journal of Cell Science*, 116(15), 3123 (2003)
95. C. Vindis, D. P. Cerretti, T. O. Daniel and U. Huynh-Do: EphB1 recruits c-Src and p52(Shc) to activate MAPK/ERK and promote chemotaxis. *The Journal of Cell Biology*, 162(4), 661-671 (2003) doi:10.1083/jcb.200302073
96. N. Sharfe, A. Freywald, A. Toro, H. Dadi and C. Roifman: Ephrin stimulation modulates T cell chemotaxis. *European Journal of Immunology*, 32(12), 3745-3755 (2002) doi:10.1002/1521-4141(200212)32:12<3745::AID-IMMU3745>3.0.CO;2-M
97. E. Asano, M. Maeda, H. Hasegawa, S. Ito, T. Hyodo, H. Yuan, M. Takahashi, M. Hamaguchi and T. Senga: Role of Palladin Phosphorylation by Extracellular Signal-Regulated Kinase in Cell Migration. *PLOS ONE*, 6(12), e29338 (2011) doi:10.1371/journal.pone.0029338
98. J. Block, D. Breitsprecher, S. Kühn, M. Winterhoff, F. Kage, R. Geffers, P. Duwe, Jennifer L. Rohn, B. Baum, C. Brakebusch, M. Geyer, Theresia E. B. Stradal, J. Faix and K. Rottner: FMNL2 Drives Actin-Based Protrusion and Migration Downstream of Cdc42. *Current Biology*, 22(11), 1005-1012 (2012) doi:https://doi.org/10.1016/j.cub.2012.03.064
99. T. Takahashi, S. Dejbakhsh-Jones and S. Strober: Expression of CD161 (NKR-P1A) Defines Subsets of Human CD4 and CD8 T Cells with Different Functional Activities. *The Journal of Immunology*, 176(1), 211 (2006)
100. C. Zheng, L. Zheng, J. K. Yoo, H. Guo, Y. Zhang, X. Guo, B. Kang, R. Hu, J. Y. Huang, Q. Zhang, Z. Liu, M. Dong, X. Hu, W. Ouyang, J. Peng and Z. Zhang: Landscape of Infiltrating T Cells in Liver Cancer Revealed by Single-Cell Sequencing. *Cell*, 169(7), 1342-1356 e16 (2017) doi:10.1016/j.cell.2017.05.035
101. P. Savas, B. Virassamy, C. Ye, A. Salim, C. P. Mintoff, F. Caramia, R. Salgado, D. J. Byrne, Z. L. Teo, S. Dushyanthen, A. Byrne, L. Wein, S. J. Luen, C. Poliness, S. S. Nightingale, A. S. Skandarajah, D. E. Gyorki, C. M. Thornton, P. A. Beavis, S. B. Fox, P. K. Darcy, T. P. Speed, L. K. Mackay, P. J. Neeson, S. Loi and C. Kathleen Cuningham Foundation Consortium for Research into Familial Breast: Single-cell profiling of breast cancer T cells reveals a tissue-resident memory subset associated with improved prognosis. *Nature Medicine*, 24(7), 986-993 (2018) doi:10.1038/s41591-018-0078-7
102. D. Abate-Daga, K.-i. Hanada, J. L. Davis, J. C. Yang, S. A. Rosenberg and R. A. Morgan: Expression profiling of TCR-engineered T cells demonstrates over-expression of multiple inhibitory receptors in persisting lymphocytes. *Blood* (2013)
103. A. H. Long, W. M. Haso, J. F. Shern, K. M. Wanhainen, M. Murgai, M. Ingaramo, J. P. Smith, A. J. Walker, M. E. Kohler, V. R. Venkateshwara, R. N. Kaplan, G. H. Patterson, T. J. Fry, R. J. Orentas and C. L. Mackall: 4-1BB costimulation ameliorates T cell exhaustion induced by tonic signaling of chimeric antigen receptors. *Nature Medicine*, 21, 581 (2015) doi:10.1038/nm.3838 https://www.nature.com/articles/nm.3838#supplementary-information
104. S. B. Vafai and V. K. Mootha: Mitochondrial disorders as windows into an ancient organelle. *Nature*, 491, 374 (2012) doi:10.1038/nature11707
105. L.-M. Charbonnier, E. Janssen, J. Chou, T. K. Ohsumi, S. Keles, J. T. Hsu, M. J. Massaad, M. Garcia-Lloret, R. Hanna-Wakim, G. Dbaibo, A. A. Alangari, A. Alsultan, D. Al-Zahrani, R. S. Geha and T. A. Chatila: Regulatory T-cell deficiency and immune dysregulation, polyendocrinopathy, enteropathy, X-linked–like disorder caused by loss-of-function mutations in LRBA. *Journal of Allergy and Clinical Immunology*, 135(1), 217-227.e9 (2015) doi:https://doi.org/10.1016/j.jaci.2014.10.019
106. T. Asaoka, T. Kato, S. Marubashi, K. Dono, N. Hama, H. Takahashi, S. Kobayashi, Y. Takeda, I. Takemasa, H. Nagano, H. Yoshida, P. Ruiz, A. G. Tzakis, K. Matsubara, M. Monden, Y. Doki and M. Mori: Differential transcriptome patterns for acute cellular rejection in recipients with recurrent hepatitis C after liver transplantation. *Liver Transplantation*, 15(12), 1738-1749 (2009) doi:10.1002/lt.21883
107. J. Zhang, C. Zhang, J. Cui, J. Ou, J. Han, Y. Qin, F. Zhi and R.-F. Wang: TRIM45 functions as a tumor suppressor in the brain via its E3 ligase activity by stabilizing p53 through K63-linked ubiquitination. *Cell Death &Amp; Disease*, 8, e2831 (2017) doi:10.1038/cddis.2017.149 https://www.nature.com/articles/cddis2017149#supplementary-information
108. H. S. Choi, C. K. Hwang, K. Y. Song, P.-Y. Law, L.-N. Wei and H. H. Loh: Poly(C)-binding proteins as transcriptional regulators of gene expression. *Biochemical and Biophysical Research Communications*, 380(3), 431-436 (2009) doi:https://doi.org/10.1016/j.bbrc.2009.01.136
109. H. S. Choi, C. S. Kim, C. K. Hwang, K. Y. Song, P.-Y. Law, L.-N. Wei and H. H. Loh: Novel function of the poly(C)-binding protein αCP3 as a transcriptional repressor of the mu opioid receptor gene. *The FASEB Journal*, 21(14), 3963-3973 (2007) doi:10.1096/fj.07-8561com
110. S. Eyerich, K. Eyerich, D. Pennino, T. Carbone, F. Nasorri, S. Pallotta, F. Cianfarani, T. Odorisio, C. Traidl-Hoffmann, H. Behrendt, S. R. Durham, C. B. Schmidt-Weber and A. Cavani: Th22 cells represent a distinct human T cell subset involved in epidermal immunity and remodeling. *The Journal of Clinical Investigation*, 119(12), 3573-3585 (2009) doi:10.1172/JCI40202
111. A. Boyhan, C. M. Casimir, J. K. French, C. G. Teahan and A. W. Segal: Molecular cloning and characterization of grancalcin, a novel EF-hand calcium-binding protein abundant in neutrophils and monocytes. *J Biol Chem*, 267(5), 2928-33 (1992)

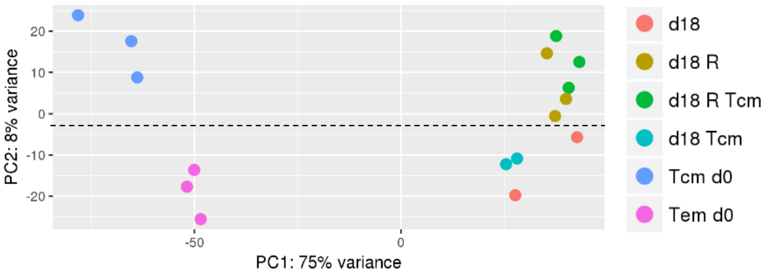


**Figure S2:** Rapamycin-treated cells are closer to *ex vivo* sorted $T_{CM}$ and non-treated cultures resemble $T_{EM}$ in dimension PC2.

Principle component (PC) analysis of RNA sequencing data of $T_{CM}$ and $T_{EM}$ sorted on d0 out of PBMCs derived from buffy coats of n = 3 HDs and untreated as well as Rapa-TCPs (R) and $T_{CM}$-like cells sorted on d18 of culture from the indicated TCP derived from the same n = 3 buffy coat donors. Lacking data points of d18 and d18 $T_{CM}$ occurred due to failure at the quality control level.

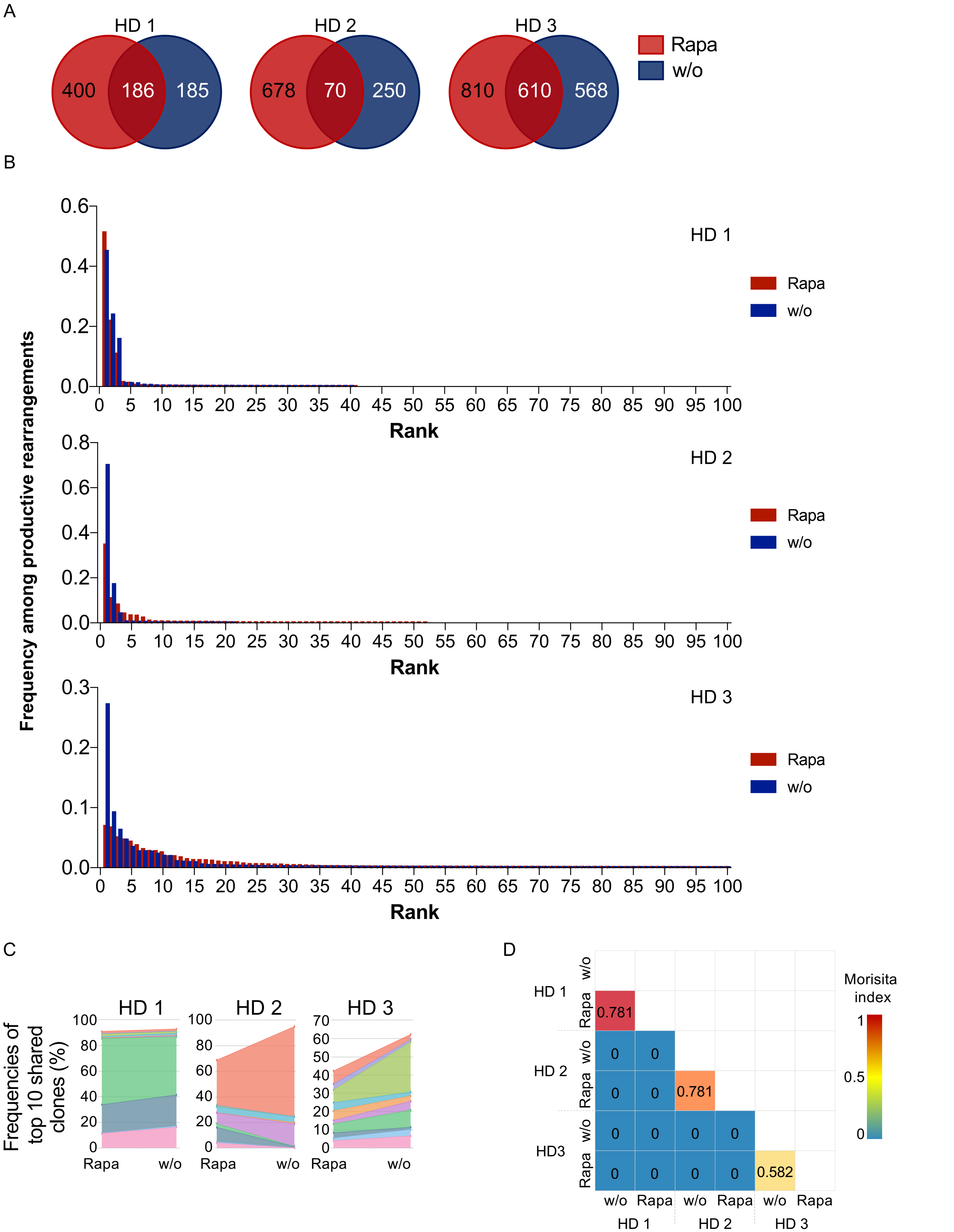


**Figure S3:** Distribution of T-cell clones among Rapa- and untreated TCPs and different donors.

**A**: Venn diagrams of all clones of Rapa- (red) and untreated TCPs (blue) of 3 HDs demonstrating individual and shared clones of each sample. **B**: Frequencies among productive rearrangements of the top100 clones of Rapa- (red) and untreated TCPs (blue) of 3 HDs. **C**: Stacked view of individual frequencies of the top 10 most represented shared clones in Rapa- and untreated TCPs (w/o) of 3 HDs. Shared clones were ranked based upon the maximum frequency achieved in either Rapa- or untreated TCPs." **D**: Morisita index matrix showing sample overlap between all samples, which is based on the presence of unique clones, individual frequencies of clones and the probability of a common origin of two samples. All data were calculated in ImmunoSEQ-Analyzer 3.0 based on TCRβ sequencing data.

**Table S2**: Patient details.

Pred. = Prednisolone; M-pred. = Methyl-prednisolone; Tac. = Tacrolimus; MMF = Mycophenolate mofetil; MPS = Mycophenolate sodium; CsA = Cyclosporine A; VGCV = Valganciclovir; ACV = Acyclovir; conc. = concentration; imcomp. = incompatible

Patients with so far no recorded CMV viremia

| No. | 1 | 2 | 3 | 4 | 5 | 6 | 7 | 8 | 9 |
|---|---|---|---|---|---|---|---|---|---|
| Age | 58 | 45 | 42 | 53 | 29 | 45 | 51 | 44 | 46 |
| Gender | M | F | F | F | M | M | F | M | M |
| time from KTx [d] | 264 | 294 | 161 | 19 | 21 | 21 | 13 | 21 | 34 |
| Time from last CMV viremia [d] | - | - | - | - | - | - | - | - | - |
| Tac. blood conc. [ng/ml] | 8.5 | 6.4 | 7.2 | 11.3 | 10 | 8.35 | 5.2 | 9.7 | 9.8 |
| Immuno-suppression at d0 | Tac. (1 mg) MMF (1,5 g) | M-pred. (4 mg) Tac. (10,5 mg) MMF (2 g) | Tac. (3 mg) MMF (1,5 g) | Pred. (20 mg) Tac. (15 mg) MMF (2 g) | M-pred. (16 mg) Tac. (18 mg) MMF (2 g) | M-pred. (4 mg) Tac. (6 mg) MMF (2 g) | M-pred. (24 mg) Tac. (8 mg) MMF (2 g) | M-pred.(4 mg) Tac. (16 mg) MMF (2 g) | M-pred. (4 mg) Tac. (6,5 mg) MPS (2 g) |
| Rejection [from d0] | acute cellular rejection (-245) | no | no | no | no | suspected acute (ATG -17 to -19) | no | no | no |
| Antiviral therapy [from d0] | none | none | none | none | none | none | none | none | none |
| Other detected CMV viremia [from d0] | none | none | none | none | none | +54 | none | +112; +7 | none |
| Lymphocyte count [/nl] | 1.04 | 1.49 | 1.48 | 2.34 | 1.23 | 0.09 | 2.34 | 2.32 | 1.2 |
| Donor | CMV-; living | CMV+; living | CMV-; living | CMV+, living | CMV+; living | CMV-; living | CMV+; living | CMV+; living | CMV-; living |
| CMV before KTx | + | + | + | + | + | + | + | + | + |
| Other | | | | | | AB0 incomp. donation, delayed graft function | | | |
| Underlying disease | IgA nephritis | hypertensive nephropathy | polycystic kidney disease | IgA nephritis | IgA nephritis | seronegative Lupus erythematosus | polycystic kidney disease | left nephrectomy after relapsed pyelonephritis; obstructive nephropathy | IgA nephritis |

Patients with recent CMV viremia

| No. | 10 | 11 | 12 | 13 |
|---|---|---|---|---|
| Age | 78 | 56 | 52 | 46 |
| Gender | M | M | M | M |
| time from KTx [d] | 694 | 27 | 2499 | 28 |
| Time from last CMV viremia [d] | 0 | 0 | 13 | 9 |
| Tac. blood conc. [ng/ml] | 6.5 | 11.5 | | |
| Immuno-suppression at d0 | M-pred. (4 mg) Tac. (3 mg) MMF (1,5 g) | M-pred. (4 mg) Tac. (10 mg) MMFl (1,5 g) | M-pred. (4 mg) CsA (130 mg) MMF (2 g) | M-pred. (4 mg) CsA (250 mg) MPS (2 g) |
| Rejection [from d0] | | | | |
| Antiviral therapy [from d0] | VGCV 900 mg (-468 to -420); 450 mg (+263 to +336); 225 mg (-668 to -599; +336 to +374) | VGCV 450 mg (19.01.2017 - 14.02.2017); 900 mg (23.11.2016 - 19.01.2017) | VGCV 450 mg (-2451 to -2423) | VGCV 900 mg (-7 to +5) |
| Other detected CMV viremia [from d0] | +400; +263; +261; +168; +119; +78; +49; +35; +16; -125; -292; -312; -348; -391; -412; -420; -475; -491; -496 | +266; -7; -11; -14; -21 | +468; +272; +97; -13; -22; -78; -267; -637; -813; -978; -1182; -1569; -2470; -2489 | -18; -25 |
| Lymphocyte count [/nl] | 0.28 | 0.47 | 0.71 | 1.21 |
| Donor | CMV+; deceased | CMV+; living | CMV+; living | CMV+; living |
| CMV before KTx | - | + | + | + |
| Other | CMV colitis (-486 to -496-, +239 to + 275); VGCV resistance excluded | | chronic hepatitis B | |
| Underlying disease | hypertensive nephropathy | IgA nephritis | unilateral renal agenesis and possibly obstructive nephropathy | Renal hypertensive angiopathy |

Patients with history of CMV viremia

| No. | 14 | 15 | 16 | 17 | 18 | 19 |
|---|---|---|---|---|---|---|
| Age | 63 | 70 | 66 | 67 | 34 | 60 |
| Gender | M | M | F | F | F | F |
| time from KTx [d] | 1809 | 296 | 380 | 563 | 665 | 7418 |
| Time from last CMV viremia [d] | 1728 | 119 | 140 | 112 | 645 | 40 |
| Tac. blood conc. [ng/ml] | 5.9 | 5.5 | 8.5 | 11.3 | 4.1 | 4 |
| Immuno-suppression at d0 | M-pred. (4 mg) Tac. (4,5 mg) | M-pred. (4 mg) Tac. (7 mg) MMF (500 ng) | M-pred. (4 mg) Tac. (3,5 mg) MMF (1,5 g) | M-pred. (4 mg) Tac. (2,5 mg) MMF (2 g) | M-pred. (4 mg) Tac. (7 mg) | Pred. (5 mg) Tac. (4,5 mg) MPS (1,08 g) |
| Rejection [from d0] | | | | | | |
| Antiviral therapy [from d0] | none | VGCV 450 mg (-1267 to -1218; -1372 to -1311) | VGCV 225 mg (-355 to -327) | none | none | ACV 200 mg (since -753) |
| Other detected CMV viremia [from d0] | none | +325; +252; -524; -708; -888; -1255; -1269 | +328; -253 | -487; -526; -535 | none | +297; +129; -207; -613; -641; -972; -2324 |
| Lymphocyte count [/nl] | 1.6 | 0.98 | 0.88 | 1.22 | 1.26 | - |
| Donor | CMV+; deceased | CMV+; deceased | CMV+; deceased | CMV-; deceased | CMV+; living | CMV+; deceased |
| CMV before KTx | + | - | + | + | + | + |
| Other | B cell lymphoma | | | | missed abortion (-1) | anal carcinoma (diagnosed +289) |
| Underlying disease | Pauci immun-glumerulo-nephritis | unclear | Diabetic nephropathy | hypertensive nephropathy | IgA nephropathy | Diabetic nephropathy |

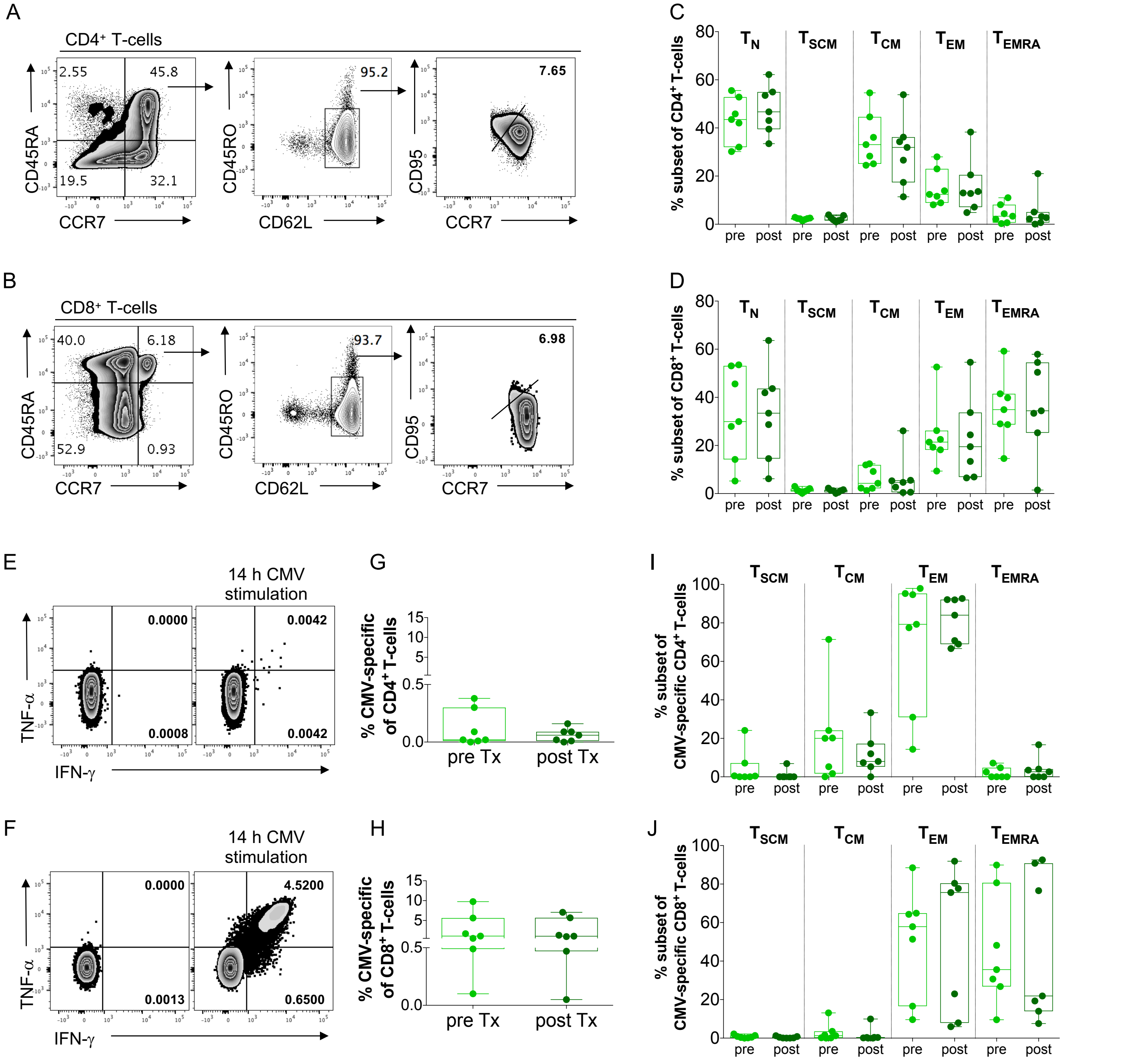


**Figure S4**: CMV-specific *ex vivo* response shifts to $T_{EM}$ on the expense of $T_{CM}$ within a few weeks after transplantation while the global subset composition remains stable.

N = 7 paired samples from the same patients before (pre; pastel green) and a few weeks after KTx (post; dark green). Gated on lymphocytes (FSC-A vs. SSC-A), single cells (FSC-H vs. FSC-A), living T-cells (CD3 vs. life/dead-discriminating dye) and as indicated on $CD4^+$ or $CD8^+$ T-cells, respectively. Gating strategy for discrimination of T-cell memory subsets for $CD4^+$ (**A**) and $CD8^+$ (**B**) subsets: $T_N$: $CD45RA^+$ $CCR7^+$ $CD95^-$; $T_{SCM}$: $CD45RA^+$ $CCR7^+$ $CD45RO^-$ $CD62L^+$ $CD95^+$; $T_{CM}$: $CD45RA^-$ $CCR7^+$; $T_{EM}$: $CD45RA^-$ $CCR7^-$; $T_{EMRA}$: $CD45RA^+$ $CCR7^-$. Global T-cell memory subset distribution of $CD4^+$ (**C**) and $CD8^+$ (**D**) T-cells from peripheral blood collected *pre*- and *post*-Tx. Subsets gated as shown in **A**/**B**. IFNγ and TNFα were stained intracellularly after 14 h stimulation of fresh PBMCs with $CMV_{IE-1/pp65}$ peptide pools and addition of BFA after 1 h. Exemplary dot plots and gating strategy for $CD4^+$ (**E**) and $CD8^+$ (**F**) T-cells. Summary of CMV-specific $CD4^+$ (**G**) and $CD8^+$ (**H**) T-cells identified as $IFN\gamma^+TNF\alpha^+$ detected *ex vivo* in PBMCs of KTx patients based on the gating strategy shown in **E**/**F**. Proportions of subsets among CMV-responsive $CD4^+$ (**I**) and $CD8^+$ T-cells (**J**) determined by characteristic expression of CCR7, CD45RA and CD95. Gates were applied from gates set for global T-cell subset distribution (**A**/**B**).

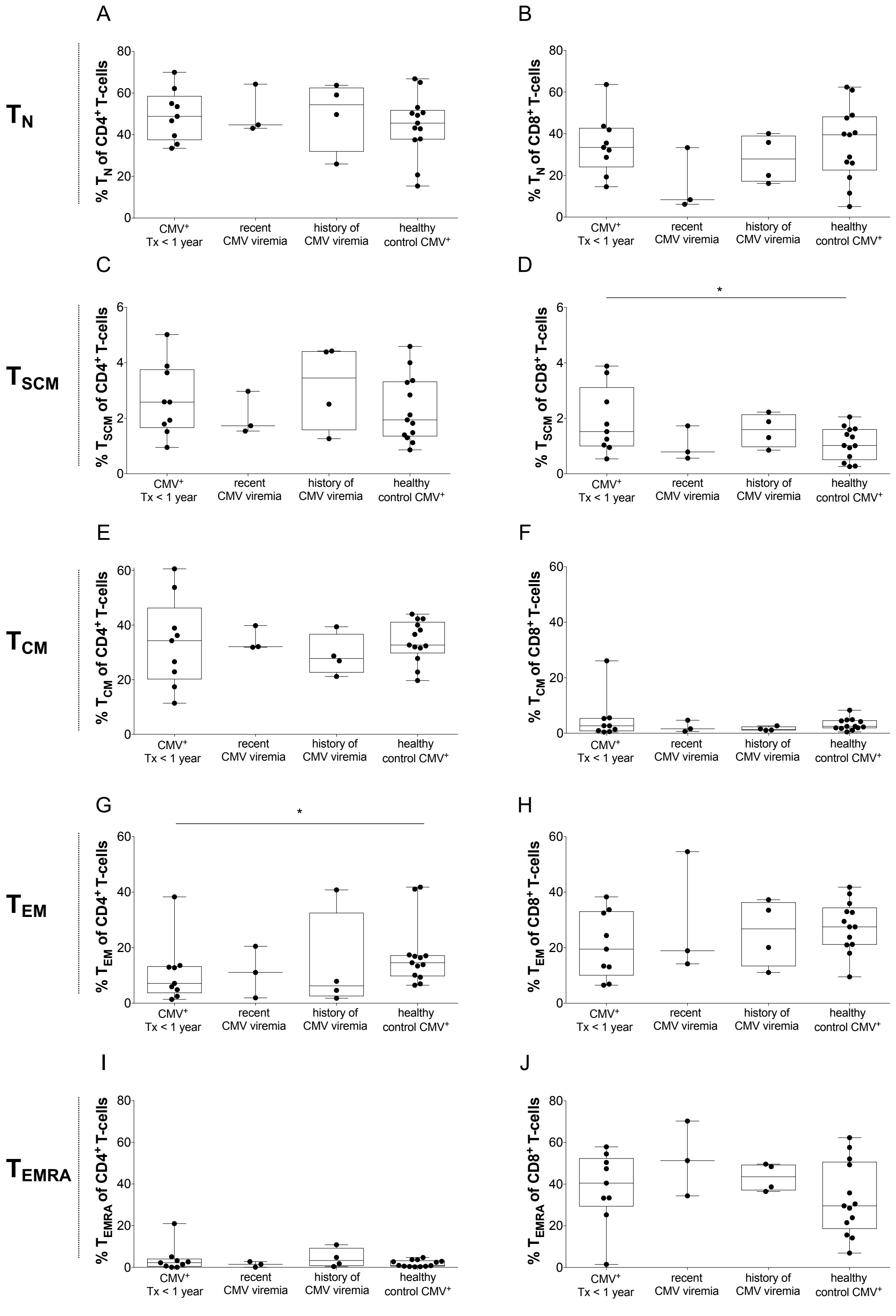


**Figure S5:** CD8$^+$ $T_{SCM}$ are globally increased and CD4$^+$ $T_{EM}$ globally decreased in the blood of patients without record of CMV viremia compared to the blood of healthy donors.

T-cell subset distribution of n = 19 patients (9 with so far no recorded CMV viremia; 4 with recent CMV viremia and 6 with a history of CMV viremia)/13 HDs. Global proportions of different CD4$^+$ (left panel) and CD8$^+$ T-cell (right panel) subsets determined by characteristic expression of CCR7, CD45RA, CD62L, CD45RO and CD95. N=19 patients (9 with so far no recorded CMV viremia; 4 with recent CMV viremia and 6 with a history of CMV viremia)/13 HDs. Gating strategy is shown in Fig. S4A/B. CD4$^+$ (**A**) and CD8$^+$ (**B**) CD45RA$^+$ CCR7$^+$ CD95$^-$ $T_N$. CD4$^+$ (**C**) and CD8$^+$ (**D**) CD45RA$^+$ CCR7$^+$ CD62L$^+$ CD45RO$^-$ CD95$^+$ $T_{SCM}$. CD4$^+$ (**E**) and CD8$^+$ (**F**) CD45RA$^-$ CCR7$^+$ $T_{CM}$. CD4$^+$ (**G**) and CD8$^+$ (**H**) CD45RA$^-$ CCR7$^-$ $T_{EM}$. CD4$^+$ (**I**) and CD8$^+$ (**J**) CD45RA$^+$ CCR7$^-$ $T_{EMRA}$. All data tested for normal distribution of data points with Kolmogorov-Smirnov test; significance determined with paired t test if normally distributed or Wilcoxon's matched-pairs signed rank test for not normally distributed samples. P-values below 0.05 are indicated by * and defined to be significant.

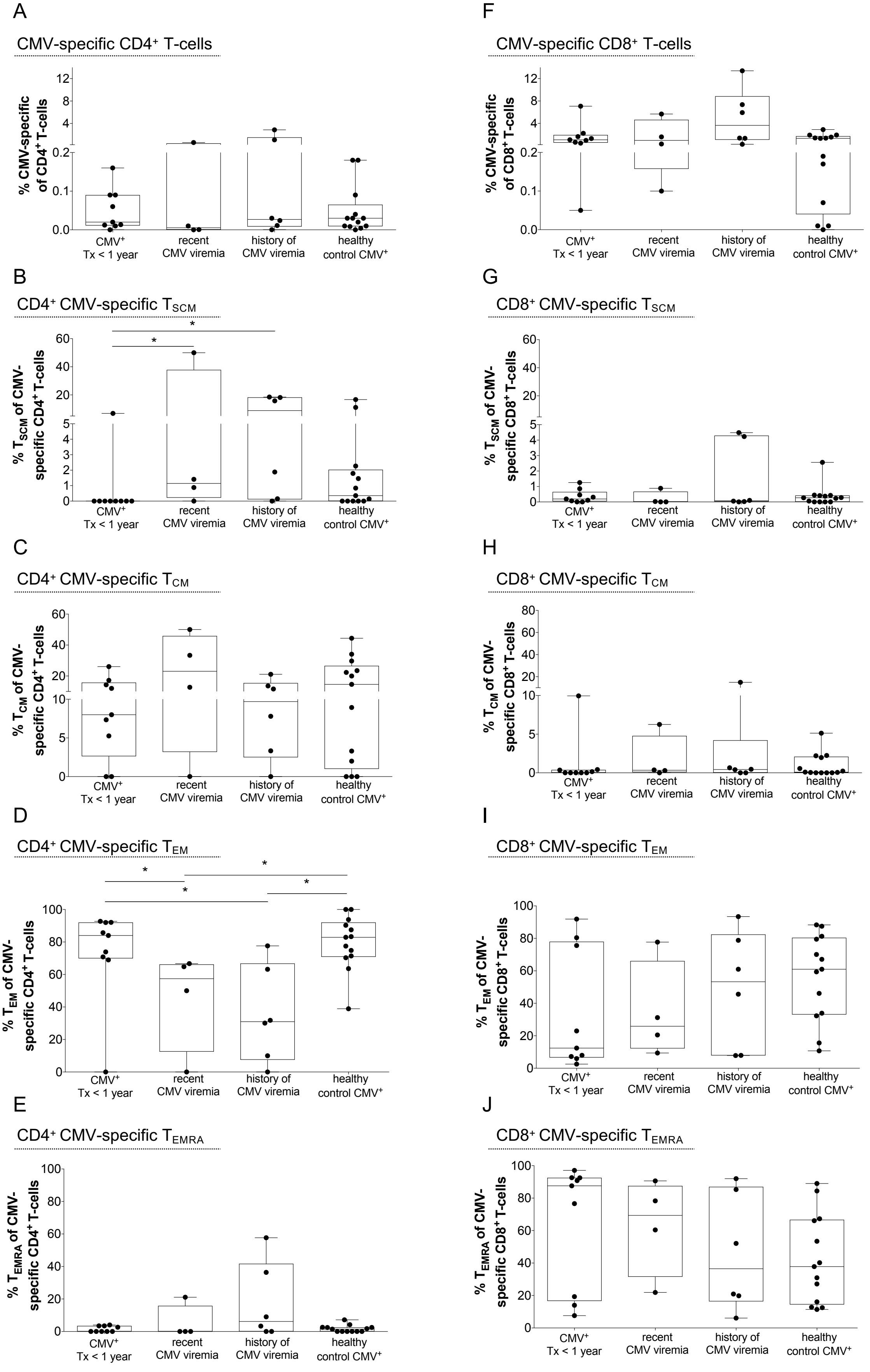


**Figure S6**: CD4⁺ CMV-specific $T_{SCM}$ accumulate upon CMV reactivation and $T_{EM}$ diminish after a history of CMV viremia in renal transplant recipients.

N=19 patients (9 with so far no recorded CMV viremia; 4 with recent CMV viremia and 6 with a history of CMV viremia)/13 healthy controls. Proportions of CMV-responsive among $CD4^+$ (**A**) and $CD8^+$ (**F**) T-cells detected by double positive intracellular staining for IFNγ and TNFα after 14 h stimulation of fresh PBMCs with $CMV_{IE-1/pp65}$ peptide pools and addition of BFA after 1 h (see dot plots Fig. 4E-F). T-cell memory subset distribution of CMV-reactive $CD4^+$ (**B**-**E**) and $CD8^+$ (**G**-**J**) T-cells following the gating strategy and subset definitions presented in Fig. S4A/B, applying gates for global subset distribution from Fig.S4. CMV-reactive $CD4^+$ (**B**) and $CD8^+$ (**G**) $CD45RA^+$ $CCR7^+$ $CD62L^+$ $CD45RO^-$ $CD95^+$ $T_{SCM}$. CMV-reactive $CD4^+$ (**C**) and $CD8^+$ (**H**) $CD45RA^-$ $CCR7^+$ $T_{CM}$. CMV-reactive $CD4^+$ (**D**) and $CD8^+$ (**I**)) $CD45RA^-$ $CCR7^-$ $T_{EM}$. CMV-reactive $CD4^+$ (**E**) and $CD8^+$ (**J**) $CD45RA^+$ $CCR7^-$ $T_{EMRA}$. All data tested for normal distribution of data points with Kolmogorov-Smirnov test; significance determined with paired t test if normally distributed or Wilcoxon's matched-pairs signed rank test for not normally distributed samples. P-values below 0.05 are indicated by * and defined to be significant.

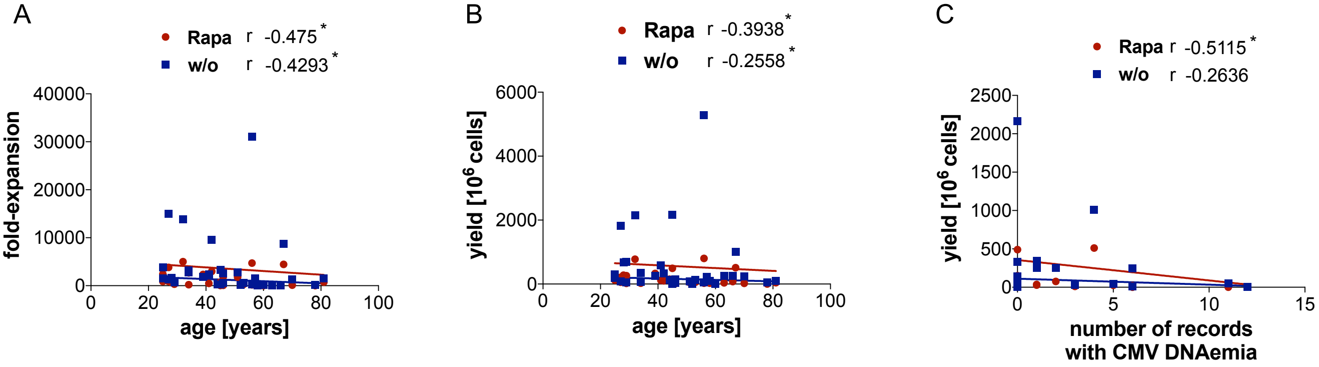


**Figure S7:** Decreased fold-expansion and yield correlate with increasing age and numbers of recorded reactivations.

Correlations of age and fold-expansion (**A**)/yield (**B**) as well as records of reactivations determined by viremia and yield (**C**) in untreated (blue) and Rapa (treated with 20 nM Rapamycin; red)-TCPs of 19 patients and 13 HDs; distributions tested for normality with Kolmogorov-Smirnov test, correlations of normally distributed data calculated with Pearson's correlation coefficient and of not normally distributed data with Spearman's rank correlation.

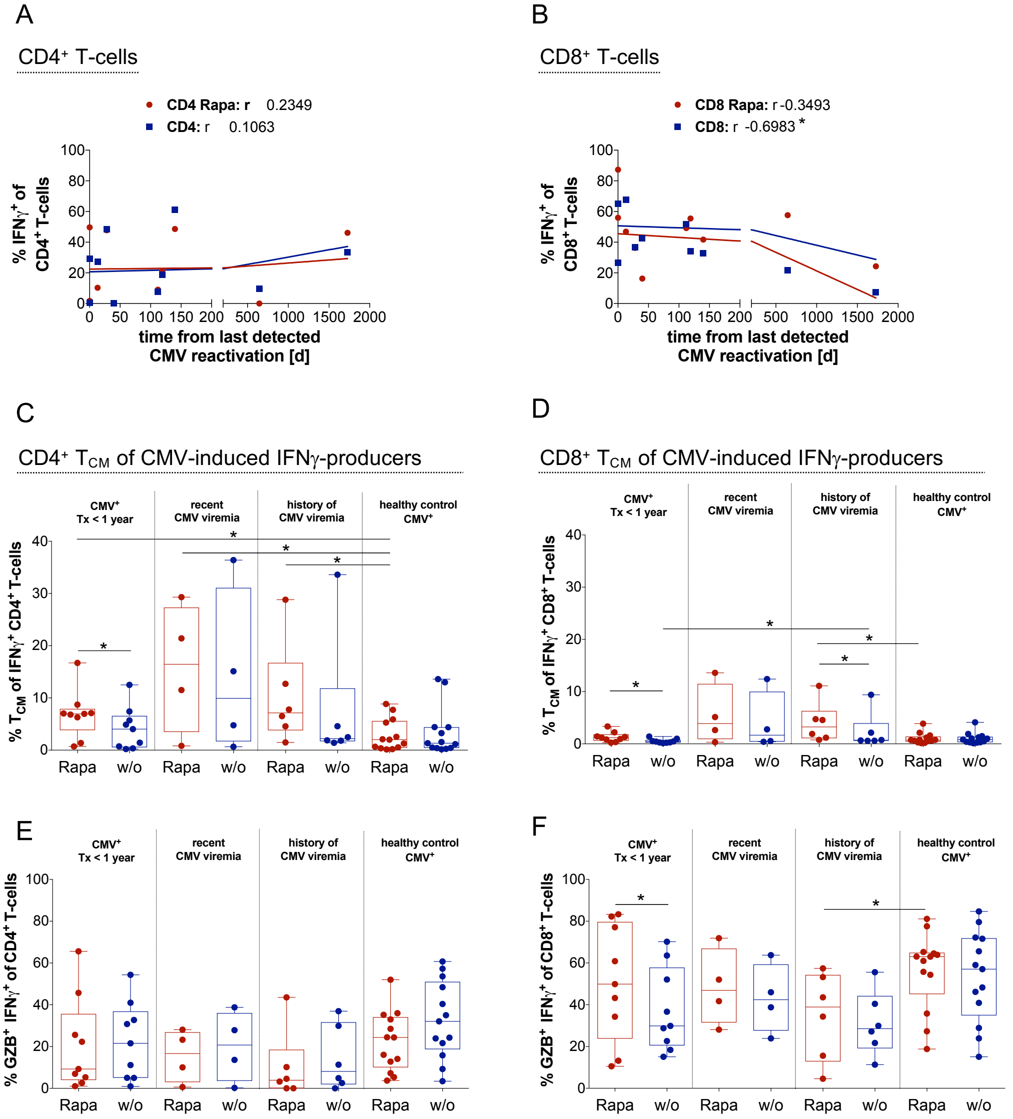


**Figure S8**: The proportion of IFNγ-producing CD8$^+$ T-cells negatively correlates with the time from reactivation in *Rapa-TCP*s, among CMV-stimulated IFNγ responders the frequency of $T_{CM}$ is increased in TCPs from patients with recorded CMV viremia and a substantial amount of cytotoxic CD4$^+$ T-cells is abundant in TCPs.

Correlations of proportions of IFNγ-producing CD4$^+$ (**A**) and CD8$^+$ (**B**) T-cells from untreated (w/o, blue) and Rapa (red)-TCPs to time from last recorded CMV DNAemia from n = 10 patients (for whom a record of CMV DNAemia was available; Table S2); distributions tested for normality with Kolmogorov-Smirnov test, correlations of normally distributed data calculated with Pearson's correlation coefficient and of not normally distributed data with Spearman's rank correlation. Proportions of CD45RA$^-$ CCR7$^+$ $T_{CM}$ among IFNγ producing CD4$^+$ (**C**) and CD8$^+$ (**D**) T-cells. Gates were applied from gates set for global T cell subset distribution (Fig.S4A-B). Proportions of CD4$^+$ (**E**) and CD8$^+$ (**F**) GZB-IFNγ-double-producers detected by intracellular staining after 6 h stimulation with autologous LCLs loaded $CMV_{IE-1/pp65}$ peptide pools and addition of BFA after 1 h on d21.

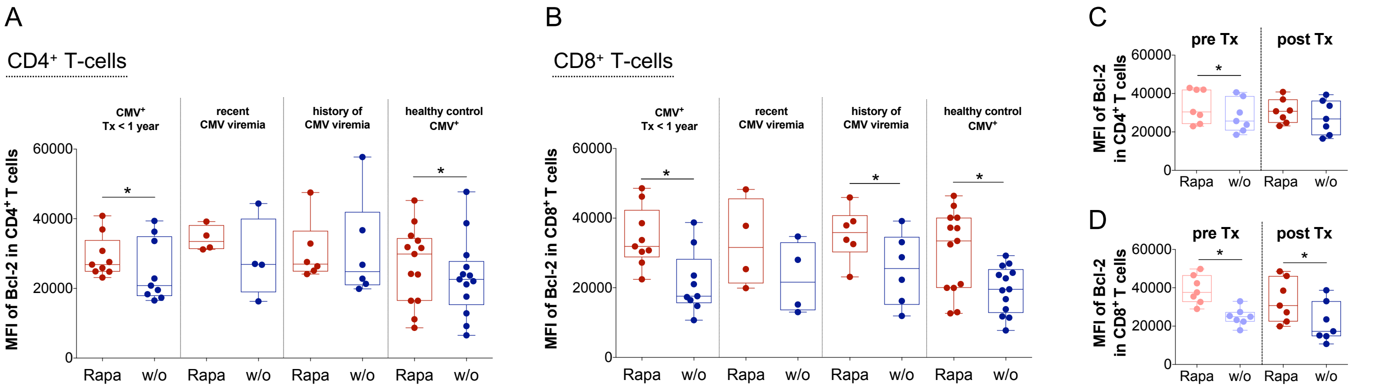


**Figure S9:** Bcl-2 is increased also in patient TCPs, especially in CD8⁺ T-cells.

MFI of Bcl-2 in CD4⁺ (**A**) and CD8⁺ (**B**) T-cells of untreated (w/o, blue) and Rapa (red)-TCPs of n = 19 patients (9 with so far no recorded CMV viremia; 4 with recent CMV viremia and 6 with a history of CMV viremia)/13 healthy controls. MFI of Bcl-2 in CD4⁺ (**C**) and CD8⁺ (**D**) T-cells of untreated (w/o, blue) and Rapa (red)-TCPs of n = 7 patients for which TCPs were generated before and a few weeks after KTx.
